# Deep phenotyping of patients with MASLD upon high-intensity interval training

**DOI:** 10.1016/j.jhepr.2024.101289

**Published:** 2024-12-16

**Authors:** Veera Houttu, Ulrika Boulund, Marian Troelstra, Susanne Csader, Daniela Stols-Gonçalves, Anne Linde Mak, Anne-Marieke van Dijk, Julia Bouts, Maaike Winkelmeijer, Xanthe Verdoes, Sandra van den Berg-Faay, Donne Lek, Ted Ronteltap, Ferdinand de Haan, Harald Jorstad, Ville Männistö, Kai Savonen, Heikki Pentikäinen, Kati Hanhineva, Ambrin Farizah Babu, Gianni Panagiotou, Otto van Delden, Joanne Verheij, Michial Doukas, Aart Nederveen, Ursula Schwab, Aldo Grefhorst, Max Nieuwdorp, Adriaan Georgius Holleboom

**Affiliations:** 1 Department of Vascular Medicine, Amsterdam UMC, University of Amsterdam, Amsterdam, The Netherlands; 2 Experimental Vascular Medicine, Amsterdam UMC, University of Amsterdam, Amsterdam, The Netherlands; 3 Amsterdam Cardiovascular Sciences Institute, Amsterdam UMC, University of Amsterdam, Amsterdam, The Netherlands; 4 Amsterdam Gastroenterology Endocrinology Metabolism Institute, Amsterdam UMC, University of Amsterdam, Amsterdam, The Netherlands; 5 Department of Radiology, and Nuclear Medicine, Amsterdam UMC, University of Amsterdam, Amsterdam, The Netherlands; 6 School of Medicine, Institute of Public Health, and Clinical Nutrition, University of Eastern Finland, Kuopio, Finland; 7 Polifysiek, Amsterdam University of Applied Science, Amsterdam, The Netherlands; 8 Department of Cardiology, Amsterdam Movement Sciences, Amsterdam Cardiovascular Sciences, Amsterdam UMC, University of Amsterdam, Amsterdam, The Netherlands; 9 Department of Medicine, University of Eastern Finland, and Kuopio University Hospital, Kuopio, Finland; 10 Kuopio Research Institute of Exercise Medicine, Kuopio, Finland; 11 Afekta Technologies Ltd., Kuopio, Finland; 12 Department of Life Technologies, Food Chemistry, and Food Development Unit, University of Turku, Turku, Finland; 13 Department of Microbiome Dynamics, Leibniz Institute for Natural Product Research, and Infection Biology, Hans Knöll Institute (HKI), Jena, Germany; 14 Faculty of Medicine, The University of Hong Kong, Hong Kong, China; 15 Faculty of Biological Sciences, Friedrich Schiller University, Jena, Germany; 16 Jena University Hospital, Friedrich Schiller University, Jena, Germany; 17 Department of Interventional Radiology, Amsterdam UMC, Amsterdam, The Netherlands; 18 Department of Pathology, Amsterdam UMC, Amsterdam, The Netherlands; 19 Department of Pathology, Erasmus MC Cancer Institute, University Medical Center Rotterdam, Rotterdam, The Netherlands

**Keywords:** Multi-omic, Gut microbiota, Metabolome, Transcriptomics, Fibrosis, Steatohepatitis, Steatosis, Histology

## Abstract

**Background & Aims:**

Exercise is a key component of lifestyle management in patients with metabolic dysfunction-associated steatotic liver disease (MASLD), but neither its therapeutic effect on the active stage of the disease, that is metabolic dysfunction-associated steatohepatitis (MASH) nor the mediating mechanisms have been characterized. Therefore, we performed multi-omic phenotyping of patients with MASLD-MASH on an exercise program.

**Methods:**

Fifteen patients with MASLD conducted high-intensity interval training (HIIT) combined with home-based training for 12 weeks. MASLD was evaluated using histology, transient elastography, and multiparametric magnetic resonance imaging (MRI) before and after the intervention. Change in maximal oxygen consumption (VO_2max_) and MRI-determined liver fat were compared with a control group of patients with MASLD (n = 22). RNA sequencing was performed on liver, muscle, and fat biopsies of patients in the exercise group. Stool was analyzed by shotgun metagenomics and untargeted metabolomics was performed on plasma, urine, adipose, and stool.

**Results:**

HIIT increased VO_2max_ by 10.1% and improved mitochondrial metabolism in skeletal muscle, indicating improved cardiorespiratory fitness and adherence. VO_2max_ increased significantly in the exercise group compared with controls. Histologically, no reduction in steatosis, MASH, or liver fibrosis was observed; however, transient elastography tended to improve. MRI-determined liver fat did not change in the exercise group compared with controls. HIIT induced changes in mRNA expression of genes related to beiging of adipose tissue and fibrogenesis in liver. In addition, specific gut microbial taxa and metabolites changed.

**Conclusions:**

HIIT increased cardiorespiratory fitness and induced beneficial gene expression changes in muscle, adipose tissue, and liver, but without translation into histological improvement of MASLD. Longer exercise intervention trials are warranted to validate or refute current recommendations for exercise as a cornerstone treatment for MASLD-MASH.

**Impact and implications:**

Despite exercise being considered as a key component of lifestyle management for steatotic liver disease, neither the clinical effects nor the mechanisms involved are completely understood. We show that a high-intensity interval training (HIIT) program in 15 patients with metabolic dysfunction-associated steatotic liver disease (MASLD) improved cardiorespiratory fitness, compared with 22 control patients with MASLD who did not participate in an exercise program, however, it did not improve MASLD. HIIT induced a positive effect on fat tissue and muscle metabolism which was accompanied with changes in certain gut bacteria and metabolites in blood and urine. These findings improve our understanding of the effects of exercise on the whole-body metabolism in relation to steatotic liver disease. As such, this study provides a basis for future exercise interventions in patients with MASLD, required to thoroughly test current guideline advice for exercise as a cornerstone treatment for MASLD of all stages.

**Clinical trial registry:**

Dutch Trial Register (registration number NL7932).

## Highlights

• HIIT improves cardiorespiratory fitness and muscle metabolism of patients with MASLD.
• The 12-week HIIT did not improve MASLD based on histology, MRI, or liver function tests.
• HIIT influenced gene expression related to adipose tissue beiging and liver fibrogenesis.
• Patients with MASLD have heterogeneous gut microbiota, but HIIT induced changes in several taxa.

## Graphical abstract

**Figure:**
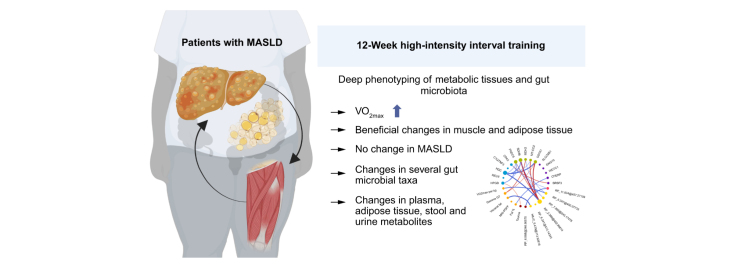
Created in BioRender. Houttu, V. (2024) https://BioRender.com/v72q218.

## Introduction

In co-occurrence with obesity and type 2 diabetes mellitus (T2DM), metabolic dysfunction-associated steatotic liver disease (MASLD)^1^ is now the primary cause of chronic liver disease, affecting 25–33% of the global population, and 55–70% of patients with T2DM.^2^^,^^3^ The spectrum of MASLD ranges from isolated steatosis to metabolic dysfunction-associated steatohepatitis (MASH) and fibrosis, which can culminate in cirrhosis and hepatocellular carcinoma. Additionally, MASLD doubles the risk of atherosclerotic cardiovascular disease.^4^ The fibrotic stages of MASLD are associated with increased liver-related and all-cause mortality.^5^

The pathophysiology of MASLD is multifactorial, occurring in the setting of obesity, insulin resistance, and a sedentary lifestyle^6^ with unhealthy dietary factors.^7^ Lifestyle modulation remains the cornerstone to prevent and treat MASLD, especially since no approved pharmacotherapy for MASLD has yet arrived.^8^ Studies report that exercise might alleviate hepatic steatosis, even in the absence of weight loss, and guidelines therefore recommend it for patients with any MASLD stage.^9^ However, the effect of exercise on the active and progressive stages of MASLD, that is MASH and fibrosis, has been poorly characterized.^10^ This is mainly because of lack of studies that combine in-depth tissue sampling with multiple methods to assess MASLD, MASH, and fibrosis, such as multiparametric magnetic resonance imaging (MRI), transient electrography, and histological analysis of liver biopsies.

Gut microbiota dysbiosis, small intestinal bacterial overgrowth, reduced gut barrier function and specific microbiome-derived metabolites such as ethanol have all been implicated to affect MASLD-MASH.[11], [12], [13] Lifestyle interventions with exercise can modulate the gut microbiome, including gut microbiota associated metabolites.^14^^,^^15^ However, the metabolic changes occurring in patients with MASLD on the level of the liver, muscle, and subcutaneous adipose tissue upon exercise, in relation to each other and to gut microbes, remain to be discerned.

We performed deep phenotyping of patients with advanced stages of MASLD during an exercise intervention to (a) examine whether exercise *per se*, that is without weight loss, can improve MASLD assessed by imaging and histology and (b) to uncover the potentially mediating metabolic processes involved.

## Materials and methods

### Study design

We conducted a single-arm 12-week exercise intervention study without altering body weight in patients with histologically characterized MASLD in Amsterdam University Medical Center, Amsterdam, The Netherlands, in compliance with the ethical guidelines of the 1975 Declaration of Helsinki as reflected in *a priori* approval by the appropriate institutional review committee. The study was approved (7/2019) by the Medical Ethics Committee of AUMC, location AMC, and registered in the Dutch Trial Register (registration number NL7932). Each participant gave informed consent in writing before the study. The study process is detailed in the CONSORT flow diagram^16^ (Fig. S1) and in Fig. S2.

### Patient inclusion

Fifteen adult participants were recruited from the local MASLD clinic. To be eligible, participants had to be aged 18–70 years, have a diagnosis of MASLD (based on liver histology, vibration-controlled transient elastography, conventional ultrasound or MRI) and a BMI <40 kg/m^2^. The main exclusion criteria were acute or chronic inflammatory or infectious diseases; excessive alcohol use (>20 units/week for men, >14 units/week for women); diagnosis of other liver diseases (such as hepatitis B, hepatitis C, autoimmune hepatitis, cirrhosis, and hepatocellular carcinoma); cardiorespiratory, neurological or musculoskeletal diseases; type 1 diabetes mellitus; inadequately controlled hypothyroidism, lipodystrophy, depression, or any mental illness rendering the patients unable to understand the purpose and procedures of the study; bleeding disorder or use of anticoagulants; contraindications for MRI scan; participation in any exercise and/or diet program more than twice a week in the 3 months before recruitment, or use of insulin or glucagon-like peptide 1 receptor agonists.

A control group of 22 patients with MASLD was included from an identical exercise intervention study conducted in the University of Eastern Finland, Kuopio, Finland.^15^ The 22 patients did not participate in the high-intensity interval training (HIIT) intervention. The design, recruitment, and inclusion criteria have been previously described.^15^ Briefly, the patients were recruited from the Kuopio University Hospital (KUH), Kuopio Health Care Centre, and Occupational Health Care. Patients with a diagnosis of MASLD based on ultrasound, MRI or computed tomography, aged 18–70 years and BMI >35 kg/m^2^ were eligible. The primary outcome was MRI-determined liver fat.

### Exercise intervention

The intervention was a 12-week HIIT program with two monitored sessions per week on a cycle ergometer (Monark ergomedic 839e, Vansbro, Sweden) (details in the Supplementary Materials and Methods). In total, participants performed 3 h of exercise per week including HIIT and home-based low-to moderate-intensity aerobic exercise, in line with international guidelines.^17^ HIIT program intensity and efficacy of the exercise intervention were determined by cardiopulmonary exercise testing (Cosmed Quark, Cosmed Omnia 2.0, Rome, Italy) on the cycle ergometer.

### Physical activity, and dietary intake monitoring

Compliance to the home-based exercise was monitored using a monitor device and questionnaires. Participants were instructed to maintain their habitual dietary intake during the study period and it was monitored by dietary food diaries. See Supplementary material for details.

### MRI of the liver and abdomen

Multiparametric MRI of liver and abdomen of the exercise group were conducted in an overnight fasted state using a clinical 3.0 T MRI unit (Ingenia; Philips, Best, The Netherlands). Liver fat content of the control patients was measured by MRI-PDFF via Siemens Avanto fit, NUMARIS/4 (1.5T); Syngo MR E11 and Siemens Aera, NUMARIS/4 machines at Kuopio University Hospital (KUH).

### Biopsies of liver, skeletal muscle, and adipose tissue

Percutaneous ultrasound-guided liver biopsies were performed in the exercise group by an interventional radiologist at the Amsterdam UMC according to standard procedures after an overnight fast. One of the participants declined follow-up liver biopsy. For each biopsy, liver samples were distributed to the clinical pathology laboratory at AMC for histology (see details in the Supplementary material). Ultrasound-guided muscle biopsies of the vastus lateralis were performed in the exercise group using a 14G biopsy needle under local anesthesia (10–20 ml of 20 mg/ml lidocaine) by an interventional radiologist according to local standard procedures after an overnight fast. Percutaneous subcutaneous adipose tissue biopsies were taken from the abdominal region by vacuum-liposuction. All tissue samples were snap-frozen in liquid nitrogen and stored at -80 °C.

### Statistical analysis

#### Clinical outcomes

Clinical outcomes and the outcomes of the cardiopulmonary exercise test were analyzed in R version 3.6.1 (R Foundation for Statistical Computing, Vienna, Austria) and IBM SPSS, version 28 (IBM, Chicago, IL, USA). See details in the Supplementary material.

#### Bioinformatic analyses

All analyses were performed in R version 4.1.3. FDR adjusted *p* values <0.05 were considered significant. See details in the Supplementary material.

## Results

### Patient baseline characteristics

Fifteen adult patients with MASLD, of whom seven had MASH, were included in the exercise intervention, and a group of 22 control patients with MASLD without exercise intervention completed the study protocol (see Table 1 for baseline characteristics). Compared with the control group, patients in the exercise group were younger (44.4 ± 13.1 *vs.* 56.7 ± 10.7 years of age, *p* <0.01) and had higher BMI (35.0 ± 4.2 *vs.* 29.5 ± 4.3 kg/m^2^, *p* <0.01) and HOMA-IR (23.8 [14.8, 43.7] *vs.* 5.9 [2.7, 7.4], *p* <0.01). Weight-adjusted cardiorespiratory fitness (VO_2max,_ ml/min/kg) at baseline was not different between the groups (24.8 ± 4.9 *vs.* 25.1 ± 5.2 ml/min/kg, *p* = 0.62), nor was MRI-determined liver fat content (19.2 ± 7.0 *vs.* 15.1 ± 11.0, *p* = 0.13).

**Table 1:** Baseline characteristics of the exercise and the control group.

	Exercise group (n = 15)	Control group (n = 22)	*p* value
Age (years)	44.4 (13.1)	56.7 (10.7)	**<0.01**
Female (n, %)	10 (66.6)	12 (48.0)	0.33
T2DM (n, %)	2 (13.3)	4 (16.0)	1.00
VO_2max_ (L/min)	2.7 (0.8)	2.2 (0.5)	**0.01**
VO_2max_ (ml/min/kg)	24.8 (4.7)	25.1 (5.2)	0.62
Power (W)	218.0 (65.5)	165.6 (57.5)	**0.02**
Weight (kg)	110.7 (20.4)	86.5 (16.0)	**<0.01**
BMI (kg/m^2^)	35.0 (4.2)	29.5 (4.3)	**<0.01**
Visceral fat (volume)	263 (108)		
Subcutaneous fat (volume)	375 (149)		
Waist circumference (cm)	112.9 (12.3)	101.6 (12.1)	**0.01**
WHR	0.99 (0.01)		
Fat mass (%)	38.5 (7.8)	30.0 (9.0)	0.10
Lean mass (kg)	64.3 (13.8)	31.8 (6.8)	**<0.01**
REE (kcal/day)	2,058 (456)	1,582 (270)	**<0.01**
Fasting glucose (mmol/L)	5.9 [5.4, 6.3]	6.0 [5.5, 6.6]	0.70
Insulin (mmol/L)	113.9 (67.0)	20.1 (10.4)	**<0.01**
HbA1c (mmol/mol)	44 [36, 46]	38 [35.5, 40.0]	0.11
HOMA-IR	23.8 [14.8, 43.7]	5.9 [2.7, 7.4]	**<0.01**
TC (mmol/L)	5.3 [4.4, 5.9]	4.8 [4.3, 5.4]	0.31
LDL-C (mmol/L)	3.5 [2.4, 2.9]	3.0 [2.7, 3.6]	0.58
HDL-C (mmol/L)	1.13 [1.0, 1.3]	1.3 [1.1, 1.6]	0.06
Triglycerides (mmol/L)	2.1 [1.1, 2.6]	1.4 [1.1, 2.0]	0.60
ALT (IU/L)	59.0 [39.5, 78.0]	49.0 [32.5, 68.5]	0.41
AST (IU/L)	38 [30.0, 65.0]	36.0 [30.5, 43.5]	0.32
γGT (IU/L)	42 [35.5, 76.5]	46.0 [25.5, 46.0]	0.93

**Elastography and MRI**			
CAP (dB/m)	341.5 (45.8)		
LSM (kPa)	9.6 [7.1, 13.4]		
Liver fat (MRI-PDFF, %)	19.2 (7.0)	15.1 (11.0)	0.13
Liver fat (MRS, %)	19.2 (6.6)		
Liver fat (3-point Dixon, %)	15.8 (7.0)		
Liver stiffness (MRE, kPa)	1.8 [1.6, 1.9]		

**Liver histology**			
Steatosis grade (0/1/2/3)	0/5/6/4		
MASLD activity score	3.7 (7.3)		
Lobular inflammation score (0/1/2/3)	1/13/1/0		
Hepatocyte ballooning score (0/1/2)	7/6/2		
Fibrosis stage (0/1/2/3/4)	1/1/9/4/0		

**Dietary intake**			
Energy intake (kcal/day)	1,895 (691)	2,272 (533)	**0.04**
Carbohydrates (g/day)	204.5 (78.5)	236.5 (57.6)	0.08
Carbohydrates (E-%)	43.4 (6.7)	41.8 (4.5)	0.20
Protein (g/day)	81.5 (32.0)	97.5 (28.3)	0.06
Protein (E-%)	17.2 (3.4)	17.1 (2.3)	0.50
Fat (g/day)	75.3 (31.0)	94.6 (26.9)	**0.03**
Fat (E-%)	36.0 (4.7)	37.3 (5.0)	0.14
Fiber (g/day)	18.3 (7.0)	26.4 (9.7)	**0.04**

Data presented for normally distributed variables are means ± standard deviation (SD) (independent *t* test), and for non-normally distributed variables are median with IQR (Mann–Whitney *U* test). Empty column entries indicate that the parameter was not collected. ALT, alanine aminotransferase; AST, aspartate-aminotransferase; BMI, body mass index; CAP, controlled attenuation parameter; dB/m, decibel per meter; E-%, energy percentage; γGT, gamma-glutamyltransferase; HbA1c, hemoglobin A1c; HDL-C, high-density lipoprotein cholesterol; HOMA-IR, homeostatic model for insulin resistance; IU/L, international unit per liter; LSM, liver stiffness measurement; MRE, magnetic resonance elastography; MRI-PDFF, multiparametic MRI; MRS, magnetic resonance spectroscopy; MASLD, non-alcoholic fatty liver disease; n, number of participants; REE, resting energy expenditure; TC, total cholesterol; T2DM, type 2 diabetes mellitus; VO_2max_, maximal oxygen consumption; W, watts; WHR, waist-to-hip ratio.

### Exercise improved cardiorespiratory fitness and affected skeletal muscle gene expression

**Fig. 1:**
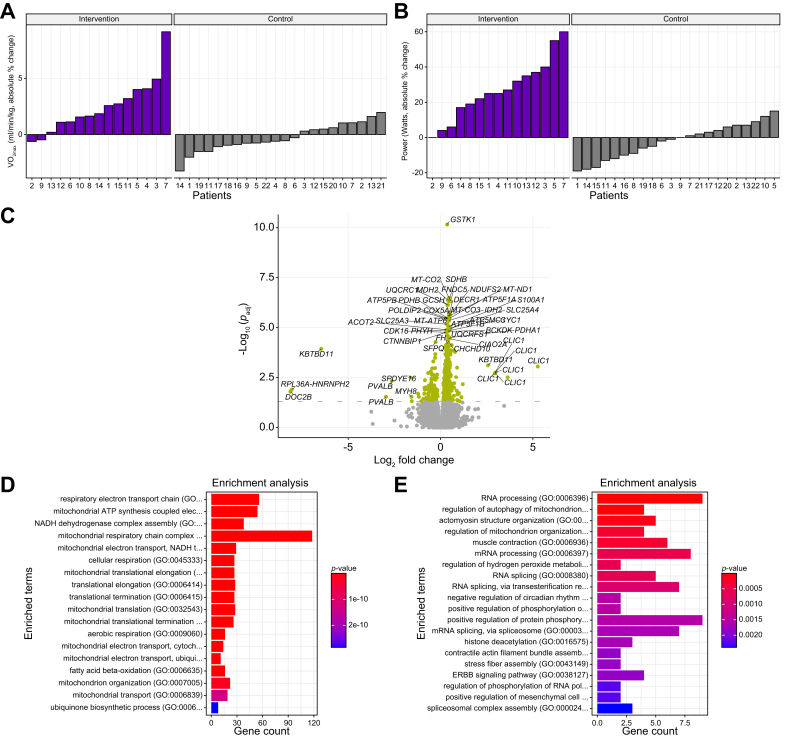
Cardiorespiratory capacity, and muscle mitochondrial metabolism increase in response to the exercise intervention. (A) Relative individual changes in VO_2max_ (ml/min/kg) upon the exercise program (exercise group n = 15) and intervention (control group n = 22). (B) Relative individual changes in power (W) upon exercise program (n = 15) and intervention (control group n = 22). (C) Volcano plot of changes in skeletal muscle gene expression upon the end of the exercise program (n = 14). Levels of significance: FDR adjusted *p* value <0.05; (Wald test, parametric fit). (D) Enrichment plot of significantly upregulated skeletal muscle genes in Gene Ontology Biological processes (2018). (E) Enrichment plot of the significantly downregulated skeletal muscle genes in Gene Ontology Biological processes (2018). FDR, false discovery rate; VO_2__max_, maximal oxygen consumption.

Fifteen patients with MASLD completed the 12-week HIIT program. Table 2 shows within-group and between-group comparisons in respect to change before and after intervention. In response to the exercise intervention, the maximum oxygen consumption (VO_2max_) increased significantly from 24.7 ± 4.7 to 27.2 ± 5.6 ml/min/kg (*p* <0.01) (Fig. 1A), indicating improved cardiorespiratory fitness and underscoring the efficacy of, and good compliance to the intervention. In line, power (218.0 ± 65.5 *vs.* 244.9 ± 72.7 W, *p* <0.01) increased significantly (Fig. 1B). In the control group, VO_2max_ did not increase (baseline 25.1 ± 5.2 *vs.* end 24.9 ± 4.8 ml/min/kg, *p* = 0.43), nor did power (baseline 86.5 ± 16.0 *vs.* end 86.6 ± 15.9 W, *p* = 0.41), rendering significant differences in VO_2max_ and power between the study groups, (*p* <0.01 for both) (Table 2, Fig. 1A and B). Body weight (as per study design), BMI, and waist-to-hip ratio did not change in the exercise or in the control group. However, in the exercise group MRI-determined visceral fat volume decreased by 5.8% despite unchanged dietary energy intake and macronutrient composition.

**Table 2:** Change in clinical outcomes upon the exercise protocol in comparison with the control group.

	Exercise group estimates (n = 15)	Exercise group *p* value	Control group estimates (n = 22)	Control group *p* value	Exercise *vs.* control estimate	Exercise *vs.* control *p* value
VO_2max_ (L/min)	**0.32 (0.08)**	**1.01e-03**	-0.03 (0.03)	4.06e-01	**0.35 (0.08)**	**4.43e-05**
VO_2max_ (mL/min/kg)	**0.44 (0.11)**	**1.15e-03**	-0.04 (0.05)	4.29e-01	**0.49 (0.11)**	**1.00e-04**
Power (W)	**0.38 (0.06)**	**2.86e-05**	-0.03 (0.03)	3.32e-01	**0.41 (0.06)**	**1.59e-07**
Weight (kg)	0.00 (0.03)	9.06e-01	0 .00 (0.02)	8.36e-01	0.00 (0.04)	9.98e-01
BMI (kg/m^2^)	0.01 (0.05)	9.06e-01	0.01 (0.03)	8.36e-01	0.00 (0.05)	9.98e-01
Visceral fat (volume)	**-0.14 (0.06)**	**3.76e-02**				
Subcutaneous fat (volume)	0 (0.09)	9.87e-01				
Waist circumference (cm)	0.06 (0.12)	6.09e-01	**0.70 (0.03)**	**1.99e-02**	-0.01 (0.10)	9.60e-01
WHR	0.16 (0.27)	5.46e-01				
Fat mass (%)	**0.12 (0.05)**	**3.81e-02**	0.06 (0.04)	2.20e-01	0.06 (0.07)	3.87e-01
Lean mass (kg)	-0.03 (0.02)	1.84e-01	-0.02 (0.01)	2.51e-01	-0.01 (0.03)	5.74e-01
REE (kcal/day)	0.06 (0.11)	5.74e-01	0.00 (0.05)	9.77e-01	0.06 (0.11)	5.54e-01
Fasting glucose (mmol/L)	0.03 (0.35)	9.23e-01	0.10 (0.05)	1.34e-01	-0.07 (0.30)	8.16e-01
Insulin (mmol/L)	-0.36 (0.22)	1.35e-01	0.06 (0.05)	2.51e-01	**-0.42 (0.19)**	**3.43e-02**
HbA1c (mmol/mol)	-0.07 (0.14)	6.24e-01	0.11 (0.07)	1.22e-01	-0.18 (0.14)	2.14e-01
HOMA-IR	-0.33 (0.23)	1.73e-01	0.08 (0.06)	1.87e-01	**-0.41 (0.20)**	**4.65e-02**
TC (mmol/L)	-0.06 (0.12)	5.90e-01	-0.11 (0.09)	2.24e-01	0.05 (0.14)	7.47e-01
LDL-C (mmol/L)	-0.36 (0.38)	3.62e-01	-0.13 (0.09)	1.47e-01	-0.23 (0.33)	4.95e-01
HDL-C (mmol/L)	0.14 (0.1)	2.02e-01	-0.1 (0.07)	1.90e-01	0.24 (0.12)	6.21e-02
Triglycerides (mmol/L)	0.05 (0.19)	8.11e-01	0.01 (0.1)	9.60e-01	0.04 (0.2)	8.41e-01
ALT (IU/L)	-0.21 (0.15)	1.76e-01	-0.05 (0.11)	6.67e-01	-0.17 (0.18)	3.62e-01
AST (IU/L)	-0.2 (0.18)	2.97e-01	-0.06 (0.14)	6.83e-01	-0.13 (0.23)	5.76e-01
γGT (IU/L)	-0.14 (0.07)	7.17e-02	-0.05 (0.06)	4.59e-01	-0.10 (0.10)	3.33e-01

**Elastography and MRI**	**Exercise group estimates (n = 15)**	**Exercise group *p* value**	**Control group estimates (n = 22)**	**Control group *p* value**	**Exercise *vs.* control estimate**	**Exercise *vs.* Control *p* value**
CAP (dB/m)	0.14 (0.27)	6.17e-01				
LSM (kPa)	-0.22 (0.11)	6.84e-02				
Liver fat (MRI-PDFF, %)	-0.09 (0.15)	5.69e-01	-0.07 (0.08)	4.36e-01	-0.02 (0.16)	8.86e-01
Liver fat (MRS, %)	-0.41 (0.27)	1.44e-01				
Liver fat (3-point Dixon, %)	-0.12 (0.25)	6.30e-01				
Liver stiffness (MRE, kPa)	-0.11 (0.12)	3.54e-01				

**Liver histology**	**Exercise group, before *vs.* after HIIT (n = 15)**	**Within group *p*** **value**				
MASLD activity score	-0.48 (0.91)	5.99e−01				
Steatosis grade (0/1/2/3)	0/5/6/4 *vs.* 0/6/4/4	0.32				
Lobular inflammation score (0/1/2/3)	1/13/1/0 *vs.* 0/12/2/0	0.16				
Hepatocyte ballooning score (0/1/2)	7/6/2 *vs.* 8/3/3	0.66				
Fibrosis state (0/1/2/3/4)	1/1/9/4/0 *vs.* 0/4/6/4/0	0.66				

**Dietary intake**	**Exercise group estimates (n = 15)**	**Exercise group *p* value**	**Control group estimates (n = 22)**	**Control group *p* value**	**Exercise *vs.* control estimate**	**Exercise *vs.* control *p* value**
Energy intake (kcal/day)	-0.13 (0.15)	3.96e-01	-0.28 (0.15)	7.54e-02	0.15 (0.22)	5.04e-01
Carbohydrates (g/day)	-0.11 (0.16)	4.91e-01	**-0.28 (0.12)**	**2.97e-02**	0.17 (0.2)	3.87e-01
Carbohydrates (E-%)	-0.28 (0.51)	5.86e-01	-0.02 (0.13)	8.95e-01	-0.26 (0.45)	5.66e-01
Protein (g/day)	0.29 (0.34)	4.08e-01	-0.22 (0.15)	1.53e-01	0.51 (0.33)	1.33e-01
Protein (E-%)	0.69 (0.46)	1.47e-01	-0.05 (0.17)	7.94e-01	0.73 (0.45)	1.12e-01
Fat (g/day)	-0.21 (0.18)	2.46e-01	-0.24 (0.19)	2.28e-01	0.03 (0.28)	9.24e-01
Fat (E-%)	-0.3 (0.28)	2.94e-01	-0.01 (0.21)	9.78e-01	-0.3 (0.34)	3.94e-01
Fiber (g/day)	0.17 (0.11)	1.55e-01	-0.19 (0.13)	1.56e-01	0.36 (0.18)	5.64e-02

Group data presented are mean difference with standard errors. For the within-group and between-group comparisons, a linear mixed-effects model was used. For ordinal outcomes a Wilcoxon test was used. Empty column entries indicate that the parameter was not collected. ALT, alanine aminotransferase; AST, aspartate-aminotransferase; BMI, body mass index; CAP, controlled attenuation parameter; dB/m, decibel per meter; E-%, energy percentage; γGT, gamma-glutamyltransferase; HbA1c, hemoglobin A1c; HDL-C, high-density lipoprotein cholesterol; HOMA-IR, homeostatic model for insulin resistance; IU/L, international unit per liter; LSM, liver stiffness measurement; MRE, magnetic resonance elastography; MRI-PDFF, multiparametic MRI; MRS, magnetic resonance spectroscopy; MASLD, non-alcoholic fatty liver disease; n, number of participants; REE, resting energy expenditure; TC, total cholesterol; VO_2max_, maximal oxygen consumption; W, watts; WHR, waist-to-hip ratio.

In line with improved cardiorespiratory fitness, exercise had strong effects on muscle mRNA expression with an upregulation of 468 and downregulation of 133 genes (Wald test, FDR adjusted *p* <0.05) (Fig. 1C–E, Table S1). Most upregulated genes relate to mitochondrial electron transport and Krebs cycle, for example mitochondrial encoded cytochrome C oxidase II (*MT-CO2*), malate dehydrogenase 2 (*MDHD2*), and succinate dehydrogenase complex iron sulfur subunit B (*SDHB*). Fibronectin type III domain containing 5 (*FNDC5*) encoding the myokine irisin,^18^ was among the strongest upregulated genes, whereas *MSTN* encoding the myokine myostatin was downregulated. Double C2 domain (*DOCB2*), a gene related to skeletal muscle insulin sensitivity,^19^ was significantly downregulated. The expression of the gene encoding insulin growth factor binding protein 2 (*IGFBP2*), a factor related to glucose metabolism,^20^ was upregulated.

Delta VO_2max_ correlated with changes in muscle mRNA expression of genes such as those encoding capping actin protein of muscle Z-line subunit beta (*CAPZD*), branched-chain keto acid dehydrogenase kinase (*BCKDK*), fumarate hydratase (*FH*), and mitochondrially encoded NADH:Ubiquinone oxidoreductase core subunit 1 (*MT-ND1*) (Table S2).

### The exercise program affected adipose tissue gene expression

**Fig. 2:**
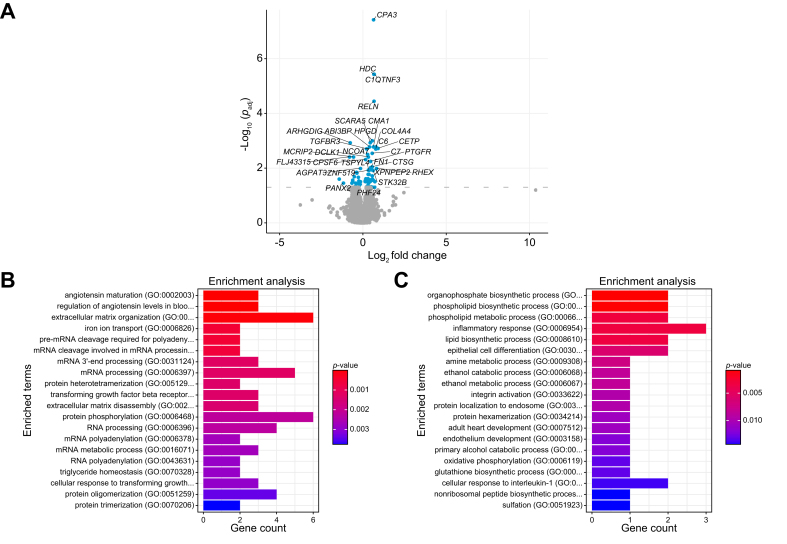
Adipose tissue transcriptomics in response to the exercise intervention. (A) Volcano plot of changes in adipose tissue gene expression upon the exercise program (n = 15). Levels of significance: FDR adjusted-value *p* <0.05; (Wald test, parametric fit). (B) Enrichment plot of the significantly upregulated adipose tissue genes in Gene Ontology Biological processes (2018). (C) Enrichment plot of the significantly downregulated adipose tissue genes in Gene Ontology Biological processes (2018). FDR, false discovery rate.

Exercise in the intervention group resulted in the upregulation of the adipose tissue mRNA expression of 57 genes whereas it reduced mRNA expression of 24 genes (Fig. 2A; Table S1). Among the upregulated genes were those encoding carboxypeptidase A3 (CPA3), which is involved in the beiging of adipose tissue,^21^ and C1QTNF3, an adiponectin homologue associated with healthier, less insulin resistant adipose tissue.^22^ In line, HOMA-IR decreased in the exercise group and was significantly different between the exercise and control group (Table 2). Biological processes of upregulated genes were related to extracellular matrix organization, angiotensin regulation, and maturation, whereas the downregulated genes were part of amine and ethanol metabolic processes (Fig. 2B and C).

### HIIT did not result in histological improvement of MASLD, but did affect fibrosis genes

**Fig. 3:**
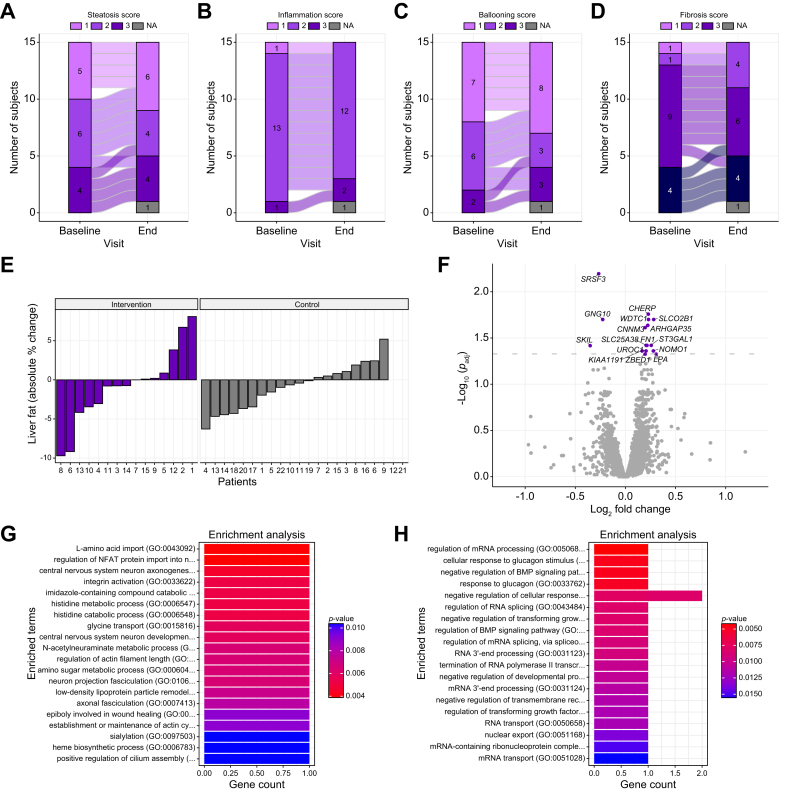
Exercise has no effect on steatosis, inflammation, ballooning or fibrosis. (A) Steatosis score, (B) inflammation score, (C) ballooning score, and (D) fibrosis score of livers at baseline and the endpoint of the exercise program. (E) Relative individual changes in MRI-PDFF-determined liver fat upon the exercise program (exercise group n = 15) and intervention (control group n = 22). (F) Volcano plot of changes in hepatic gene expression upon the exercise program (n = 13). Levels of significance: FDR adjusted *p* value <0.05; (Wald test, parametric fit). (G) Enrichment plot of the significantly upregulated liver genes from Gene Ontology Biological processes (2018). (H) Enrichment plot of the significantly downregulated liver genes in Gene Ontology Biological processes (2018). FDR, false discovery rate.

On average, the 12-week HIIT in the exercise group did not affect histological steatosis (Z = -1.0, *p* = 0.32), inflammation (Z = -1.4, *p* = 0.16), hepatocyte ballooning (Z = -0.45, *p* = 0.66) or fibrosis (Z = 0.45, *p* = 0.66). In line, NAFLD activity score (NAS) did not change (3.7 ± 7.3 *vs.* 3.8 ± 9.0, *p* = 0.74). Large interindividual differences in the hepatic response to HIIT were observed (Fig. 3A–D). Steatosis assessed with MRI-proton density fat fraction (PDFF), 3-point Dixon, and magnetic resonance spectroscopy (MRS) also did not improve in the exercise group, or in the control group (Table 2). Interindividual variability in changes on MRI-PDFF were seen in both groups, ranging from 10% reductions to a 10% increase in the exercise group, and from 7% reductions to 6% increase in the control group (Fig. 3E). Although the FibroScan® (Echosens, Paris, France) controlled attenuation parameter for steatosis did not improve, liver stiffness measurement tended to be lower after exercise (Table 1).

HIIT increased the hepatic mRNA expression of 13 genes and decreased the expression of three genes (Fig. 3F and Table S1). Of interest, four of the altered genes relate to liver fibro-inflammation, *SKIL,* encoding SKI like proto-oncogene,^23^ *SRSF3,* splicing factor 3b subunit 3, *FN,1* encoding fibronectin 1,^24^ and *ARHGAP35,* Rho GTPase activating protein 35.^25^ Among the upregulated biological processes that these upregulated genes relate to were L-amino acid import, nuclear factor of activated T-cells (NFAT) protein regulation, and integrin activation (Fig. 3G). Downregulated biological processes were related to cellular response to glucagon, signaling pathway of bone morphogenetic protein (BMP), and regulation of mRNA processing (Fig. 3H).

### Highly variable individual fecal microbiota composition

**Fig. 4:**
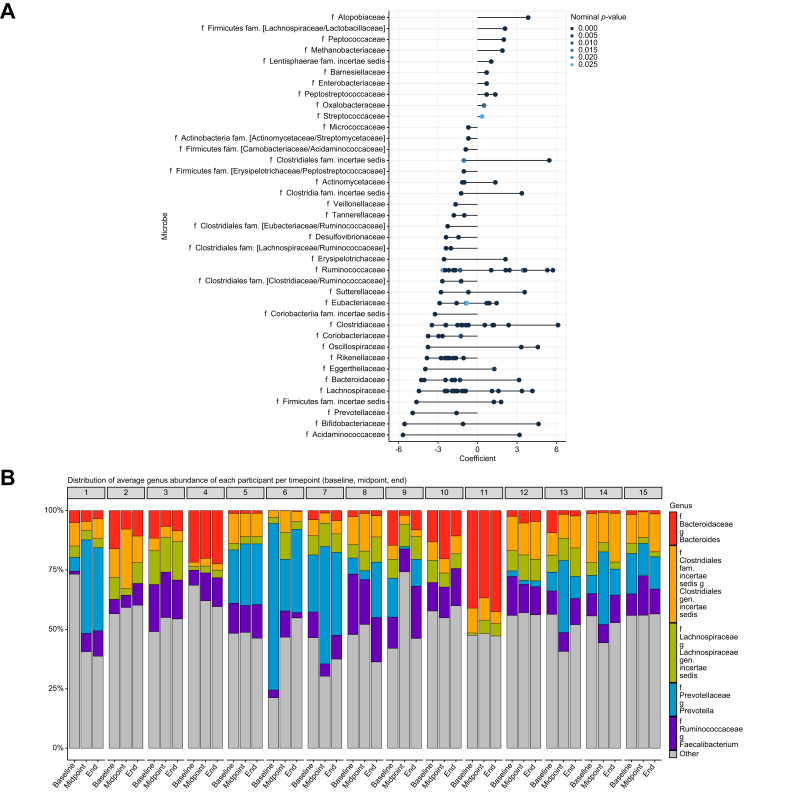
Gut microbiota composition upon exercise intervention. (A) Linear mixed model regression coefficient of the fecal microbial species in response to the exercise intervention, visualized on family level, the color represents the nominal *p* value >0.05 (n = 15). (B) Enterotypes of patients (n = 15) in different time points of the exercise intervention.

Next, we used metagenomic analysis of stool samples of the exercise group at the baseline, midpoint, and endpoint of the intervention. There was no significant difference in α-diversity (Shannon index, and evenness), richness, and β-diversity (Bray-Curtis) between these time points (Fig. S4, Table S5). However, the abundance of specific microbial species was affected. Specifically, members of the phyla Euryarcheaota and Lentisphaera, the latter previously linked to steatosis grade in MASLD.^26^ increased in relative abundance, whereas members of Proteobacteria, Bacteroidetes, Actinobacteria, and Firmicutes exhibited either an increase or decrease in abundance (Fig. 4A). Enterotype analysis revealed a great microbial heterogeneity among the patients already before the intervention (Fig. 4B). The abundance of microbial pathways upon exercise showed a nominally significant change in six pathways, but none passed adjustment for multiple testing (Table S6).

### Metabolomics of stool, plasma, urine, and adipose tissue in response to the exercise program

**Fig. 5:**
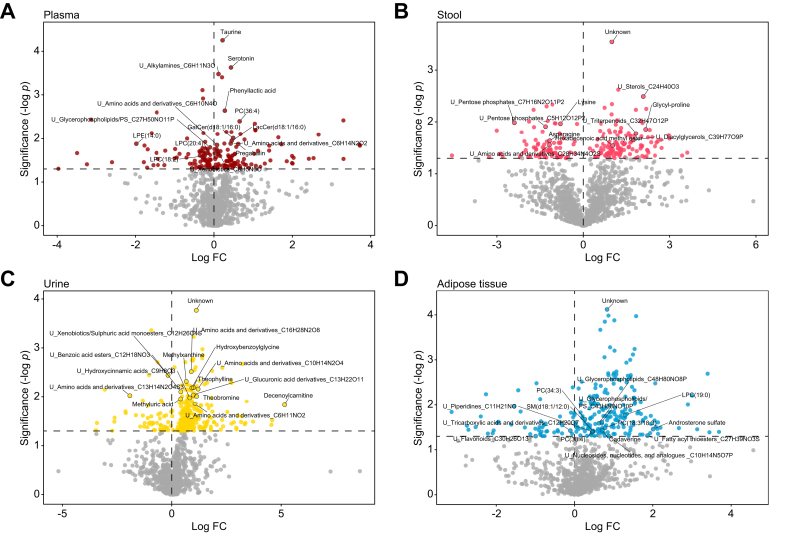
Effect of the exercise intervention on the metabolome of plasma, stool, urine, and adipose tissue. Volcano plots of results of a linear mixed effect model, the x-axis represents the log_2_ fold change of metabolite abundance between baseline and endpoint, the y-axis represents the negative log_2_ of the nominal *p* value >0.05. U refers to unknown feature, with known compound class, and formula (A) of plasma metabolome changes upon the intervention (n = 14); (B) stool metabolome (n = 15); (C) urine metabolome (n = 15); (D) adipose tissue metabolome (n = 14).

Untargeted metabolomic profiling of plasma, stool, urine, and adipose tissue at baseline and the end in the exercise group revealed significantly altered abundance of five metabolites in urine, two in plasma, one in adipose tissue, and one in stool (Fig. 5A–D, Table S7). Exercise decreased the abundance glycerophospholipids in plasma but increased their abundance in adipose tissue (Table S6, Fig. S5A–D). Taurine abundance increased upon exercise.

### Crosstalk between liver, muscle, and adipose tissue upon exercise

**Fig. 6:**
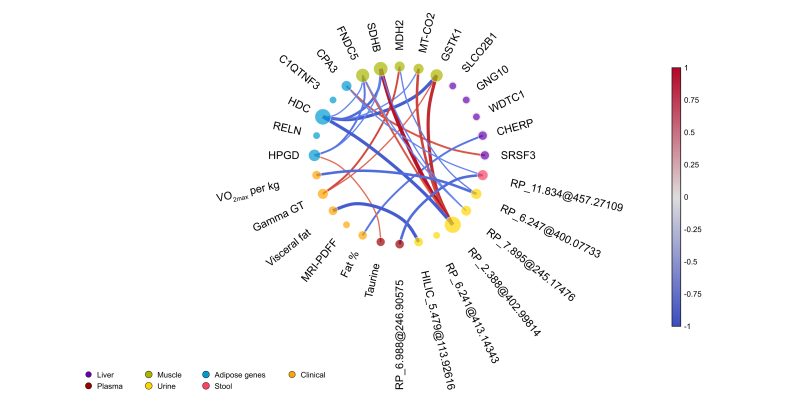
Associations of relative changes across different tissues and clinical outcomes upon the exercise intervention. The top five of the most significant FDR-corrected variables are included per data type. The significantly altered clinical outcomes, as well as the primary outcome (MRI-determined liver fat, MRI-PDFF) are included. Spearman correlations (Rho >0.6, *p* <0.05) are illustrated by red (positive) or blue (negative) lines. Circle size reflects the number of correlations, and line size reflects correlation coefficient. For transcriptomics, purple color represents liver genes, green skeletal muscle, blue adipose tissue; for metabolomics red represents plasma, pink stool, yellow urine, and blue adipose tissue; orange color represents clinical outcomes. CPA3, Carboxypeptidase A3; FDR, false discovery rate; FNDC5, Fibronectin type III domain containing 5; GSTK1, glutathione S-transferase kappa 1; HDC, Histidine decarboxylase; HPGD, 15-hydroxyprostaglandin dehydrogenase; MDH2, malate dehydrogenase 2; MRI, magnetic resonance imaging; MT-CO2, Mitochondrial encoded cytochrome C oxidase II; PDFF, proton density fat fraction; SDHB, Succinate dehydrogenase complex iron sulfur subunit B; SRSF3, Splicing factor 3b subunit 3; VO_2__max_, maximal oxygen consumption.

Inter-relations of exercise-induced changes of clinical outcomes, tissue gene expression, and metabolomic features (Fig. 6, Table S9A and B) revealed that changes in hepatic *SRFS3* mRNA expression correlated positively with changes in adipose tissue *CPA3* mRNA expression in the exercise group. Moreover, changes in muscle *FNDC5* mRNA expression correlated with adipose tissue mRNA expression of 15-hydroxyprostaglandin dehydrogenase (*HPGD*) and histidine decarboxylase (*HDC).* The unknown urinary metabolite Rpneg_2.388@402.998 correlated with muscle mRNA expression of *FNDC5*, *SDHB, MT-CO2*, and glutathione S-transferase (*GST1),* but also with adipose tissue *HDC* mRNA expression. Finally, changes in adipose tissue *HPGD* mRNA expression correlated with changes in plasma taurine abundance. Additionally, a Procrustes analysis demonstrated that the liver and adipose transcriptome were significantly correlated after the intervention (Table S11, *p* = 0.003, correlation coefficient = 0.76) and that the adipose and stool metabolome were significantly correlated with each other after the intervention (Table S10, *p* = 0.038, correlation coefficient = 0.55).

## Discussion

This study provides a comprehensive characterization of the effects of a 12-week HIIT intervention without weight loss in patients with MASLD-MASH. The intervention improved cardiorespiratory fitness induced expression of genes involved in beiging of subcutaneous adipose tissue, altered expression of genes involved in liver fibrosis, and tended to lower liver stiffness. However, despite the improved cardiorespiratory fitness and beneficial changes at the gene expression level, liver histology and MRI outcomes did not show improvement of MASLD in this study. The modest reduction in visceral fat in absence of a reduction of MASLD corroborates another study.^27^ This may indicate that visceral fat is more sensitive to exercise-induced metabolic improvement than hepatic fat.

Large interindividual differences in the hepatic response to HIIT were observed, as exemplified by the MRI-PDFF data assessing liver steatosis. A responder/non-responder pattern is also commonly observed in pharmacological interventions.^28^ Of note, we also observed interindividual differences in MRI-PDFF change in the control group, potentially reflecting the waxing- and waning nature of MASLD.^29^ The fact that the changes in VO_2max_ in the exercise group did not translate into an effect on MASLD suggests that increased cardiorespiratory fitness alone is not sufficient to induce a histological effect on MASLD in the timeframe studied.

With respect to the magnitude of cardiorespiratory improvement, it is interesting to compare the patients with MASLD in our study with a recent study in 48 healthy individuals with an average BMI of 23 kg/m^2^.^30^ This study reports an increase in VO_2max_ of almost 16% upon comparable HIIT exercise intervention, whereas we only observed an increase of 10.1%. Of note, exercise effort was comparable between the studies, with a power increase of 13% in Rodriguez-Garcia *et al.*^30^ and 12% in our study. This may raise the hypothesis that patients with MASLD and their related cardiometabolic comorbidities may have reduced capability of training effect compared with patients who were non-MASLD, which could bear relevance for clinical practice.

When comparing clinical parameters upon exercise intervention with the multi-omics data, we did observe significant inter-tissue correlations in the patients with MASLD, indicative of modulation of various metabolic tissues by the exercise. The improved cardiorespiratory fitness upon the exercise program was reflected by changes in muscle mRNA expression. Most of the affected genes encode proteins involved in energy metabolism, indicative of an enhanced aerobic capacity of skeletal muscle.^31^ More specifically, muscle fat oxidation may have increased relatively more strongly than carbohydrate oxidation, as reflected by increased mRNA expression of *ACOT2* encoding acyl-CoA thioesterase 2 that hydrolyzes CoA esters, and of *DECR1* encoding a protein that participates in β-oxidation.^32^^,^^33^ This might indicate that the HIIT improved the efficiency of muscular energy substrates usage. Exercise also upregulated muscle mRNA expression of *FNDC5* that encodes a precursor of irisin, a myokine secreted in response to exercise.^34^ Irisin might promote browning of white adipose tissue and thereby increase adipocyte fatty acid oxidation and lipolysis^35^ resulting in increased energy expenditure.^36^ We also observed upregulation of *CPA3* in adipose tissue, as a potential sign of browning. Irisin-induced browning of adipose tissue^36^ may reduce hepatopetal lipid fluxes and inflammation*,* important steps in the induction of MASLD.^18^ Interestingly, changes in *FNDC5* mRNA expression correlated with changes in the adipose tissue mRNA expression of, for example *HPGD* and *HDC*. Exercise reduced the muscle mRNA expression of *MSTN* encoding the myokine myostatin that is associated with muscular atrophy and insulin resistance.^37^ This is in line with another study in which exercise decreased plasma myostatin concentrations in MASLD.^38^

Current guidelines endorse exercise for all patients with MASLD, regardless of the severity of MASLD.^9^ These guidelines are based on studies in which the steatotic component of MASLD on imaging data. The few exercise studies that do use histological analysis to score MASH and fibrosis show contradictory results. While O’Gorman *et al.*^39^ report that HIIT regressed hepatocyte ballooning and fibrosis, Eckhard *et al.*^40^ found no histological improvements upon HIIT. These contradictory effects might be attributable to established limitations of using liver biopsies, such as sampling variability.^41^ We therefore assessed MASLD non-invasively with multiparametric MRI and transient elastography and found concordant results of MRI with histological outcomes.

The lack of improvement of MASLD assessed by MRI is discordant with a previous exercise study.^10^ Our study has comparable intervention and duration as the previous study but was without weight loss and included advanced MASLD stages. Together, this suggests that although our study demonstrates significant improvements of respiratory fitness and a clear change in metabolically relevant genes in muscle and adipose tissue, weight loss,^42^ or a longer intervention duration^40^ are likely needed to achieve improvement in MASLD.

Most studies exploring the effects of exercise on gut microbiomes are conducted in athletes rather than in patients.^43^ We are among the first to report the effect of exercise on gut microbes in MASLD and show that the relative abundance of members of the phyla Euryarcheaota and Lentisphaera increased with exercise.

Our multi-omics analyses showed that the exercise-induced changes in plasma taurine correlated negatively with adipose tissue mRNA expression of *HPGD* encoding a protein involved in hydroxylated fatty acid oxidation.^44^ Taurine has been shown to alleviate MASH,^45^ and a recent study showed provoked mitochondrial metabolism in the adipose tissue of patients with obesity when exercise was combined with taurine supplementation,^46^ underscoring the potential clinical relevance of taurine in correcting metabolic diseases.

Our study has several strengths. Firstly, this study is among the few that investigate the effect of exercise on MASLD with histological endpoints alongside MRI and transient elastography. Hence, our study can serve as a blueprint to design controlled exercise intervention studies in MASLD. Secondly, our study is unique as it focused on the effect of exercise on MASLD, and patients therefore continued their habitual diet to maintain their body weight. This exemplifies that the present results are a result of the direct effect of exercise rather than calorie restriction and/or weight loss. Finally, we conducted the most deeply phenotyped study of exercise in MASLD to date using multiple omics layers across several biologically relevant compartments, raising new mechanistic hypotheses that warrant further research, for example the potential rapid response to exercise of fibrogenic gene transcription. These strengths counterbalance the main limitation of our study, the modest sample size owing to COVID-19 pandemic restrictions on inclusions.

In conclusion, in patients with MASLD, an exercise intervention without effect on body weight improved cardiorespiratory fitness, however to a lesser degree than reported for those without liver disease,^30^ and did not ameliorate MASLD. This may indicate that MASLD is best treated with combined lifestyle interventions resulting in weight loss, potentially even combined with weight loss medication as published recently.^47^ This most deeply characterized multi-omics HIIT intervention uniquely unravels the crosstalk between multiple metabolic tissues and contributes greatly to the biological understanding of the mechanistic effects of exercise in MASLD. Further exercise interventions in severe stages of MASLD are needed to validate guideline recommendations for exercise as a cornerstone treatment.

## Abbreviations

ARHGAP35, ARHGAP35 rho GTPase activating protein 35; BCKDK, Branched-chain keto acid dehydrogenase kinase; BNP, Bone morphogenetic protein; CAPZD, Capping actin protein of muscle Z-line subunit beta; CPA3, Carboxypeptidase A3; DOCB2, Double C2 domain; FH, Fumarate hydratase; FNDC5, Fibronectin type III domain containing 5; FN1, Fibronectin 1; GST1, Glutathione S-transferase; HDC, Histidine decarboxylase; HIIT, High intensity interval training; HPGD, 15-hydroxyprostaglandin dehydrogenase; IGFBP2, Insulin growth factor binding protein 2; MASH, Metabolic dysfunction-associated steatohepatitis; MASLD, Metabolic dysfunction-associated steatotic liver disease; MDHD2, malate dehydrogenase 2; MRI, magnetic resonance imaging; MRE, magnetic resonance elastography; MRS, Magnetic resonance spectroscopy; MT-CO2, Mitochondrial encoded cytochrome C oxidase II; MT-ND1, Mitochondrially encoded NADH:Ubiquinone oxidoreductase core subunit 1; MSTN, Myostatin; NFAT, Nuclear factor of activated T-cells; PDFF, Proton density fat fraction; SDHB, Succinate dehydrogenase complex iron sulfur subunit B; SKIL, SKI like proto oncogene; SRSF3, Splicing factor 3b subunit 3; VO_2__max_, Maximal oxygen consumption.

## Financial support

This project has received funding from the European Union's Horizon 2020 research, and innovation program under the Marie Skłodowska-Curie grant agreement No 813781. MN is supported by a personal ZONMW-VICI grant 2020 (09150182010020) and an ERC-Advanced grant 2023 (101141346). AGH is supported by the Amsterdam UMC Fellowship grant, the Amsterdam UMC Innovation grant, the Dutch Gastroenterology Foundation MLDS, Holland∼Health TKI-PPP and Horizon Europe GRIP on MASH. KH is supported by ERA-Net NEURON (grant no 334814), and Academy of Finland (grant no 321716).

## Authors’ contributions

Study concept and design: VH, UB, SC, VM, GP, US, AG, MN, AGH. Acquisition of data: VH, SC, DS-G, JB, DL, TR, FDH, US. Statistical analysis: VH, UB. Computational analysis: UB. Interpretation of data: VH, UB, AG, AGH. Technical and material support: MT, DS-G, ALM, A-MD, MW, XV, SB-F, DL, TR, HJ, KS, HP, OD, AN, MN. Technical and data analytical support on metabolomics analysis: KH, AFB, AGH. Imaging data analysis: MT, SB-F, AN. Liver histology analysis: JV, MD. Administrative support: MN, AGH. Study supervision MN, AGH. Obtained funding: GP, MN. Drafting of the manuscript: VH. Contributed to the intellectual content of the manuscript, edited, and approved the final draft of the manuscript: all authors.

## Data availability statement

Data are available upon request.

## Conflicts of interest

MN is scientific advisor of Caelus Health, however this is not relevant for the content of the current paper.

Please refer to the accompanying ICMJE disclosure forms for further details.
