# Supplementary material for: Deep phenotyping of patients with MASLD upon high-intensity interval training
Source: JHEP Rep. 2024 Dec 16;7(3):101289. doi: 10.1016/j.jhepr.2024.101289 (PMC11883402; doi:10.1016/j.jhepr.2024.101289)
Supplement: Multimedia component 1 [file mmc1.pdf]

Deep phenotyping of patients with MASLD upon high-intensity interval training

Veera Houttu, Ulrika Boulund, Marian Troelstra, Susanne Csader, Daniela Stols-Gonçalves, Anne Linde Mak, Anne-Marieke van Dijk, Julia Bouts, Maaike Winkelmeijer, Xanthe Verdoes, Sandra van den Berg-Faay, Donne Lek, Ted Ronteltap, Ferdinand de Haan, Harald Jorstad, Ville Männistö, Kai Savonen, Heikki Pentikäinen, Kati Hanhineva, Ambrin Farizah Babu, Gianni Panagiotou, Otto van Delden, Joanne Verheij, Michial Doukas, Aart Nederveen, Ursula Schwab, Aldo Grefhorst, Max Nieuwdorp, Adriaan Georgius Holleboom

Table of contents

Supplementary methods 2

Supplementary statistical analysis 7

Supplementary figures 11

Supplementary table legends........ 16

Supplementary references 17

Supplementary methods

High-intensity interval training program

Each HIIT session involved 10-minute warming-up (at 30 % of intensity), repeated bouts of 2–4 minutes high intensity intervals (85% of intensity) interspersed by three minutes of active recovery intervals (10 % of intensity), and followed by 5-minute cooling-down (at 20 % of intensity) where the intensities were based on the baseline CPET. Intensity was determined as Wmax4 referring to workload that is sustainable for four minutes(1). The session was repeated twice per week on non-consecutive days for 12 weeks during which each high intensity interval of each session was increased by two seconds. At the end of the intervention period, sessions were approximately 50-minute long with 4-minute high intervals. Sessions were conducted in a group of 1-3 participants closely supervised by the researchers and/or the exercise physiologist (V.H., J.B., D.L.) at the Polifysiek, Hogeschool van Amsterdam, Amsterdam, the Netherlands.

Physical activity, and dietary intake monitoring

An objective physical activity monitoring device (Polar Active, Kempele, Finland) was used to monitor the compliance. Leisure time physical activity was assessed by the modified Minnesota Leisure-Time Physical Activity Questionnaire (2,3). This was monitored on 4–5 consecutive days (at least 1 weekend day) at the baseline, midway and at the end of the intervention using online dietary intake lists (exercise group: dietary record application https://mijn.voedingscentrum.nl/nl/eetmeter/; control group: 4-day food diaries from nutrient intakes were calculated using AivoDiet sofware (version 2.2.0.0, Aivo Finland Oy, Turku, Finland) at the baseline and week 11). Both dietary intake and physical activity diaries were checked and assessed at the time of the collection by the researchers and a clinical nutritionist.

Magnetic resonance imaging of the liver and abdomen

Magnetic resonance spectroscopy (MRS), magnitude-based MRI (MRI-M) proton density fat fraction (PDFF), and three-point Dixon were performed to assess liver fat content. The images of MRS, MRI-PDFF, and three-point Dixon were analyzed in a blinded fashion by a single analyst. Magnetic resonance elastography for liver fibro-inflammation and volumes of visceral, and subcutaneous abdominal fat were quantified blinded by a single analyst. Liver fat content of the control patients analysis was performed blinded at Amsterdam UMC as described (4).

Vibration controlled transient elastography

Vibration controlled transient elastography (VCTE, FibroScan) was performed in the exercise group under fasted conditions using a FibroScan® 530 Compact (Echosens, France) with either M- or XL-probe per protocol to assess steatosis with controlled attenuation parameter and liver fibrosis with liver stiffness.

Liver histology

Hematoxylin & eosin and Sirius Red staining of the liver slices were performed according to standard protocols after which they were scored blinded by two pathologists in tandem. The NASH Clinical Research Network was applied to score fibrosis stages (5). The steatosis, activity, and fibrosis score was used to score steatosis; lobular inflammation; and hepatocellular ballooning (6). This score classified the cases into MASH if ballooning with lobular inflammation was present, and when scored fibrosis stage ≥ F2, cases were classified fibrotic MASH.

Body composition, and resting energy expenditure

Waist circumference was determined at the midpoint between the lateral iliac and the lowest rib. Hip circumference was determined at the widest position of the buttocks. Calf circumference was determined at the widest position of the calf when knee kept in 90°. Body composition was determined by bioelectrical impedance analysis (exercise group: Tanita DC-430 MA, Tokyo, Japan; control group: Inbody 720 body composition analyzer, Inbody, USA) in standing position. Resting energy expenditure via measuring oxygen consumption and carbon dioxide production was determined by indirect calorimetry using a computerized flow-through canopy gas-analyzer system (exercise group: Vmax Encore 29; SensorMedics, Anaheim, CA, USA; control group: Cosmed Quark, RMR, Italy).

Biochemistry

Plasma was isolated from overnight fasted blood samples and stored at –80 until biomarker concentrations were measured with routine clinical analytical biochemistry.

Urine samples

Participants collected twenty-four-hour urine using a plastic 3L container. Participants were asked to keep the container refrigerated during the collection period. Samples from the container were stored at –80°C.

Fecal sample collection

Participants collected morning fecal samples using collection tubes with screw-in spoon lid at baseline, mid-, and endpoint, and were stored at –80 °C. If morning fecal sampling was not possible, participants provided a sample from the previous evening.

Tissue RNA extraction, and sequencing

The RNA extraction of liver, muscle, and adipose tissue was done using an RNA isolation protocol optimized for small tissue biopsies. Samples were mixed with 300 µl of TriPure Isolation Reagent (Roche, Basel, Switzerland) and homogenized on ice using a sterile RNA free pestle. After 60 µl of chloroform was added, the samples were placed in Heavy Phase Lock gel tubes (Quanta Bio, Beverly, USA) and centrifuged for 15 minutes, at 4˚C, 12000xg. The aqueous phase was mixed with 70% ethanol and placed on RNeasy MinElute spin columns (QIAgen, Tegelen, the Netherlands). RNA was washed according to the protocol of the manufacturer and eluted in 14 µl of RNAse free water. The concentration of RNA was determined using Biotek Synergy H1 (Agilent Tech, Santa Clara, USA). One liver sample had low RNA yield and was therefore discarded (in total 28 liver samples were further processed). Furthermore, the RIN scores were obtained using TapeStation (Agilent Tech, Santa Clara, USA). T-oligo attached magnetic beads were used to purify messenger RNA from total RNA. The mRNA was fragmented and cDNA was synthesized using random hexamer primers. Thereafter end repair, A-tailing, adapter ligation, size selection amplification and purification was performed. The RNA sequencing of 28 liver samples was conducted using HiSeq 4000 (Illumina) with 150-bp paired-end reads, and 7.5 G raw data per sample at Novogene Co., Ltd. Sequence data has been deposited at the European Genome-phenome Archive under accession number EGAS00001006991.

Fecal DNA extraction and sequencing

Fecal DNA was extracted from 45 fecal samples using a modification of a previously described protocol(7). In short, 250 mg fecal material was lysed using repeated bead beating in STAR buffer (Roche Diagnostics). Total genomic DNA was isolated from the lysates using a Maxwell device (RSC Blood Kit, Qiagen) and DNA was eluted in ~60ul nuclease free water. The library preparation and sequencing for shotgun metagenomics were performed using a HiSeq Illumina instrument at Novogene Co., Ltd 150-bp paired-end reads and 7.5 G raw data per sample.

Sample preparation, and liquid chromatography-mass spectrometry (LC-MS) analysis

Plasma, urine, stool, and adipose tissue samples for the untargeted metabolite profiling were prepared as previously described(4). Plasma, stool, urine, and adipose tissue samples from 15 subjects except one end point sample in plasma (missing), and adipose tissue were analysed using liquid chromatography (reverse-phase, and hydrophilic interaction liquid chromatography), and mass spectrometry with an ultra-high performance liquid chromatography system (Vanquish Flex UHPLC system, Thermo Scientific, Bremen, Germany) coupled to a high-resolution mass spectrometer (Q Exactive Focus, Thermo Scientific, Bremen, Germany). The data was acquired in positive (ESI+), and negative (ESI−) electrospray ionization modes. Data dependent MS2 were acquired for each mode. The technical details of the chromatographic methods, and LC-MS instrument configurations have been described previously(8).

Data matrix generation of metabolomics

Automated peak picking and alignment was done by MS-DIAL (Version 4.90)(9) after the conversion of the raw instrumental data (*.d files) to an ABF format using Reifycs Abf Converter (https://www.reifycs.com/AbfConverter), as previously described(4). A total of 236,666 features were obtained from the peak-picking from the four analytical modes.

Data analysis of metabolomics

Data pre-processing was done separately for each sample matrices and analytical modes using R version 3.6.2. Low-quality features were flagged and discarded from the main results of statistical analyses. Molecular features were only considered high-quality if they met all the following quality metrics: low number of missing values (present in more than 70% of the QC samples, present in at least 50% of samples in at least one study group). Missing values were imputed using simple imputation with value of 0 for all features.

Metabolite identification

For the metabolite identification, only those features with a MS/MS spectrum, average peak area of at least 10,000 per sample type, and raw p-value <0.05 fromthe feature-wise paired t-tests were selected. These chosen signals were annotated using MS-DIAL Version 4.90(9) by

comparing the exact m/z, retention time, and MS/MS fragmentation patterns against our in-house standard library (ca. 1000 metabolites). Further, additional searches in online MS spectral databases were also performed(10–13). Additionally, MS-FINDER Version 3.52(14) was used to characterize the unknowns. Moreover, the vendor software FreeStyle 1.3 was used for the exploration of raw data extracted ion chromatograms (EICs) and MS/MS fragmentation spectra.

Additionally, fragment similarity searches in METLIN database(11) were performed for the top 10 compounds resulting from the analysis (feature-wise) with Maaslin2 version 1.8.0(15) with the formula feature ~ visit with Participant ID as random effect.

Supplementary statistical analysis

Clinical outcomes

Differences in baseline clinical characteristics between the groups were tested using paired T-test if the data were normally distributed. Skewed data were tested by Mann Whitney's U-test. The distribution of the data was assessed by Kolmogorov–Smirnov test as well as by visual inspection of histograms, and Q-Q plots. To test differences between the time points, for continuous outcomes two types of linear mixed models were fit. For the within group comparisons, the data was subset per intervention group and a linear mixed effects model (lmer in R package lme4 version 1.1-34) was fit using the following formula: outcome ~ visit + (1 | ID). For the between group comparisons the entire dataset was used and a linear mixed effects model (lmer in R package lme4 version 1.1-34) was fit using the following formula: outcome ~ visit * intervention + (1 | ID). All outcomes were scaled before fitting the model. The following variables were log transformed before scaling for the linear model analyses: VO_{2max} (L/min), $VO_{2max/}$ (ml/kg/min), weight, BMI, waist circumference, lean mass, fasting glucose, insulin, HbA1c, HOMA-IR, TC, LDL-C, triglycerides, ALT, AST, GGT, liver fat (MRI-PDFF), liver stiffness (MRE), carbohydrates (E-%) and protein (E-%). This selection was based on visual inspection of QQ-plots and Shapiro-Wilk test for normality, to ensure the necessary assumptions were met.

Statistical analysis of tissue RNA expression

The average read count per sample was 49,7 +- 6,6 million. The reads were quality controlled with Trimmomatic version 0.38 with the following parameters: minimum length after trimming = 36, sliding window width = 4, sliding window threshold q score = 15, headcrop = 5. The quality-controlled reads were then pseudo-mapped with Kallisto version 0.45 to *Homo sapiens* transcriptome GRCh38 release 106 with the following parameters: -b 100 –bias. A quality control analysis was performed, where one baseline muscle sample was removed due to close clustering with all adipose tissue samples based on a principal coordinate analysis of the Bray-Curtis distance of all samples (see Supplementary Fig. 3).Only participants with paired samples were kept (liver: 13, muscle: 14, adipose tissue: 15). Differential gene expression was tested per sample type (liver: 29,443 genes, adipose tissue: 32,468 genes, muscle: 29,516 genes) (DESeq2 (16) version 1.34.0, formula: Gene ~ participant ID + visit, with a Wald test and parametric fit). Gene set enrichment was estimated with enrichR (17) version 3.1 with GO Biological Process (2018). Details of deposited data in Supplementary CTAT Table.

Statistical analysis of gut microbiota composition

The average read count per sample was 47,1 +- 6,4 million. Shotgun sequencing reads were quality controlled, and adapters were trimmed with fastp(18) version 0.20.0, on average 46,7 +- 6,5 million reads passed filtering (99%). The trimmed reads were mapped to *Homo sapiens* genome GRCh37 hg19 using Bowtie 2(19) version 2.3.5 (with parameters—very-sensitive, and –dovetail), on average 0.13% +- 0.21% of reads mapped to the human genome. Reads that did not map to the human genome were profiled with mOTUs(20) version 3.0.1. The relative abundance of 1,417 microbial taxa was quantified using mOTUs. In total 244 species present in >75% of participants were tested for differential abundance (ANCOM-BC version 1.4.0 (21), formula: taxon ~ ID + visit, based on the raw count data). Alpha diversity was calculated using the R package vegan version 2.6-4 (function diversity, the parameter index was set to shannon), richness was calculated using the function specnumber, and eveness was calculated as Shannon index / log(richness). Beta diversity was calculated using the vegdist function, with method set to bray or jaccard. Beta diversity was also calculated with the Aitchison distance (chemometrics package version 1.4.2 function clr with a pseudocount of 1 added to the count data, then vegdist with method set to euclidean). Difference in alpha diversity between the visits was tested with a paired Wilcoxon rank sum test (two sided) or Kruskal-Wallis test (two-sided). Difference in beta diversity between the visits was calculated with a permutation multivariate analysis of variance (PERMANOVA), function adonis2 (distance ~ visit, permutations = 9999) with permutation strata by participant ID. Multivariate dispersions of the beta diversity was calculated with the betadisper function with a pairwise permutation test. Enterotypes were calculated based on unfiltered counts at genus level, using Bray-Curtis distance (calculated as explained above) with partitioning around medoids clustering using the clustering R package version 2.1.4 . The number of clusters were determined based on the Calinski-Harabasz index from the fpc R package version 2.2-99, and the most abundant taxa per cluster was used to characterize the clusters.

Microbial pathway analysis

The metagenome reads that passed filtering and did not map to the human genome were processed with HUMANn3 with the search-mode uniref90 to generate microbial functional profiles. The data was normalized to counts per million, and nonstratified data was extracted. The unintegrated, and unmapped pathways were removed, which yielded 414 pathways. The

pathway data was then filtered by first selecting pathways with a median abundance in the top 75%, and then by keeping pathways with a variance in the top 50%. This resulted in 156 pathways that was analyzed with paired Wilcoxon test.

Statistical analysis of metabolites

After the preprocessing, and data clean-up, 11,306 molecular features in plasma samples, 8,393 in adipose tissue samples, 24,171 in urine samples, and 25,166 in stool samples were considered of high quality. The high number of molecular features before data clean-up is due to the high sensitivity of the instrument, collecting several signals from each actual metabolite, but also from the solvent background, and detector noise. For the purpose of metabolite identification, feature-wise paired t-tests were run for all baseline-endpoint pairs in each sample type, and considered significant if the raw p-values were <0.05. Thereafter, all features were filtered to have an average retention time between 1-15 minutes, have MS/MS spectra available, features with zero variance were excluded, and per sample type, features that were present in <50% of any study group were also removed, and for highly correlated pairs of features, only one was kept (using the findCorrelation function from the caret package version 6.0-93, with Spearman R>0.75 as cut-off). The fold change was calculated for each feature to measure the effect size as log2(baseline/end). Only participants with paired samples were kept for analysis (plasma: 14, stool: 15, urine: 15, adipose tissue: 14). In total 1,838 features in plasma, 1,117 features in adipose tissue, 2,158 features in urine, and 2,637 features in stool were analyzed with a linear model (Maaslin2 version 1.8.0(15), formula: feature ~ visit, with Participant ID as random effect).

Multiomics correlation analysis

The baseline, and endpoint omics data (transcriptome, metabolome, metagenome) in the exercise group were compared using Procrustes analysis (in R version 4.1.3 vegan package version 2.6-4). This required paired samples, thus across all datasets 12 samples were used, transcriptomics: 12, and metabolomics: 15. The data was Hellinger transformed (decostand from the vegan package). PCA was performed (rda function from the vegan package), which was rotated, and rescaled using the Procrustes function with the parameter symmetric set to TRUE. Finally, the rotations were tested with a permutational test using the protest function, with 9999 permutations. Additionally, a cross-tissue correlation analysis was performed in Python version 3.7.3 with Conda version 4.7.10. Delta changes (calculated as (End-

Baseline)/(Baseline+End)) of the 5 most FDR p-value significant features from each omic datasets were correlated (Spearman's rho) across datasets. Samples from 8 participants that overlapped across all omics dataset were used. Correlations with rho>0.6 and p-value <0.05 are plotted.

Supplementary figures

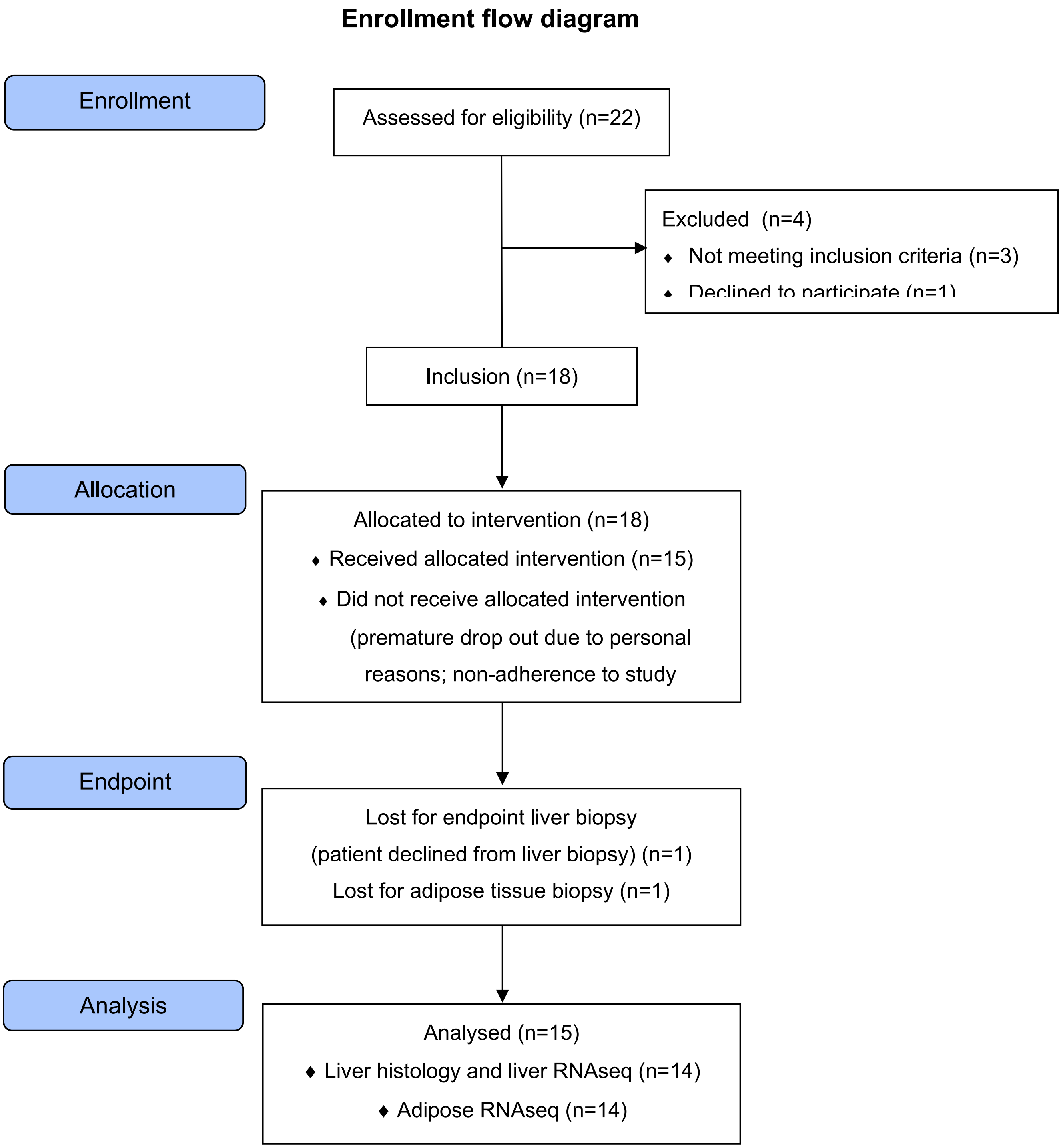

Fig. S1. Enrollment flow diagram adjusted CONSORT 2010 Flow Diagram (22).

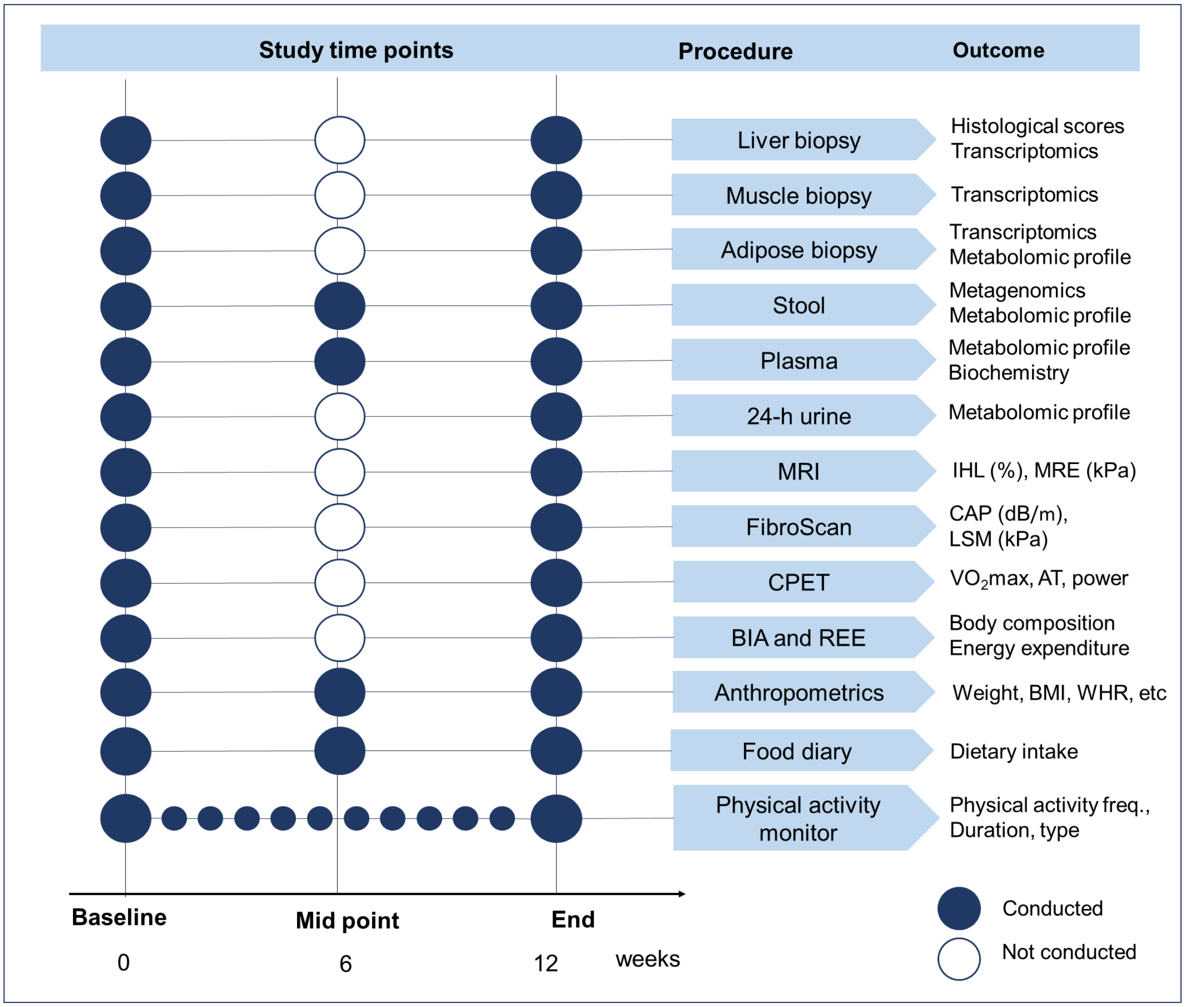

Fig. S2. Study design, procedures and outcomes during the 12-week exercise program. Dark blue circle presents on which time point (baseline, midpoint and endpoint) a procedure was conducted, white circular when a procedure was not conducted. BMI, body mass index; CAP, controlled attenuation parameter; CPET, cardiopulmonary exercise test; dB/m, decibel per meter; LSM, liver stiffness measurement; MRE, magnetic resonance elastrography; MRI, magnetic resonance imaging; kPa, kilopascals; REE, resting energy expenditure; WHR, waist-hip-ratio.

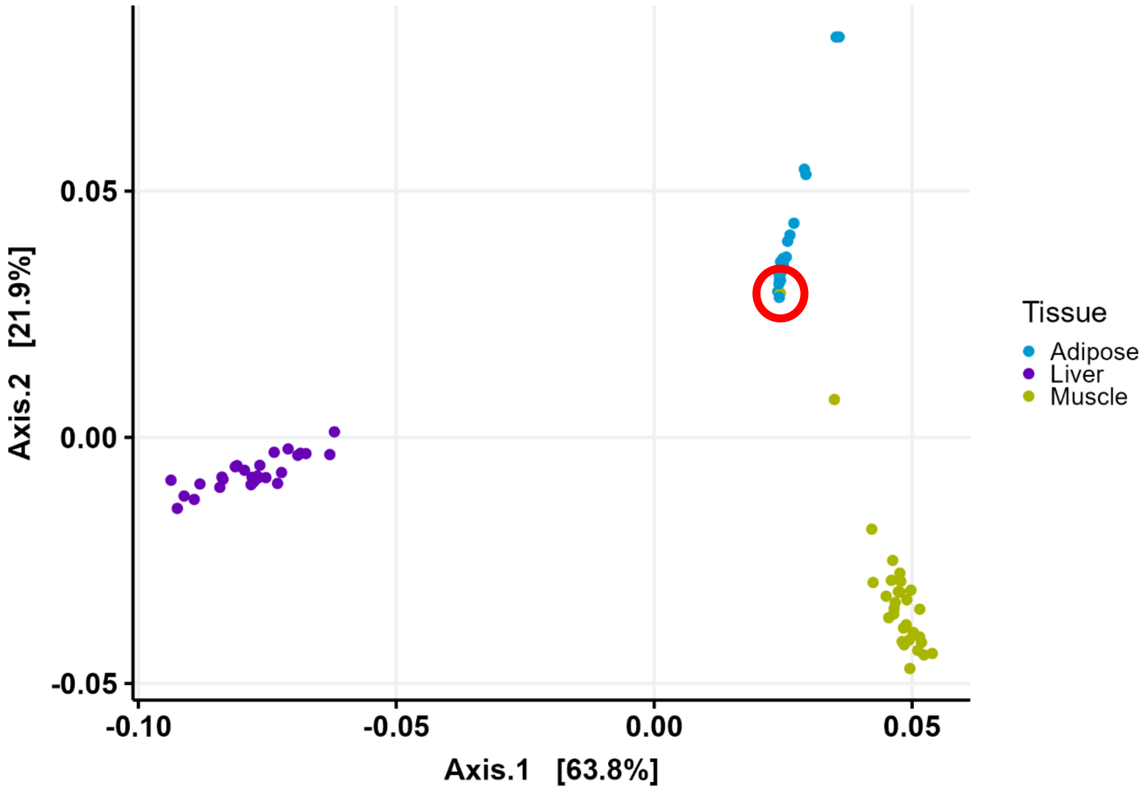

Fig. S3. Quality control of the tissue mRNA sequencing. Liver, adipose and muscle biopsy samples in principal coordinate analysis of the Bray-Curtis distance. Note one muscle (green) sample is clustered within the adipose tissue samples (blue), highlighted in the red circle.

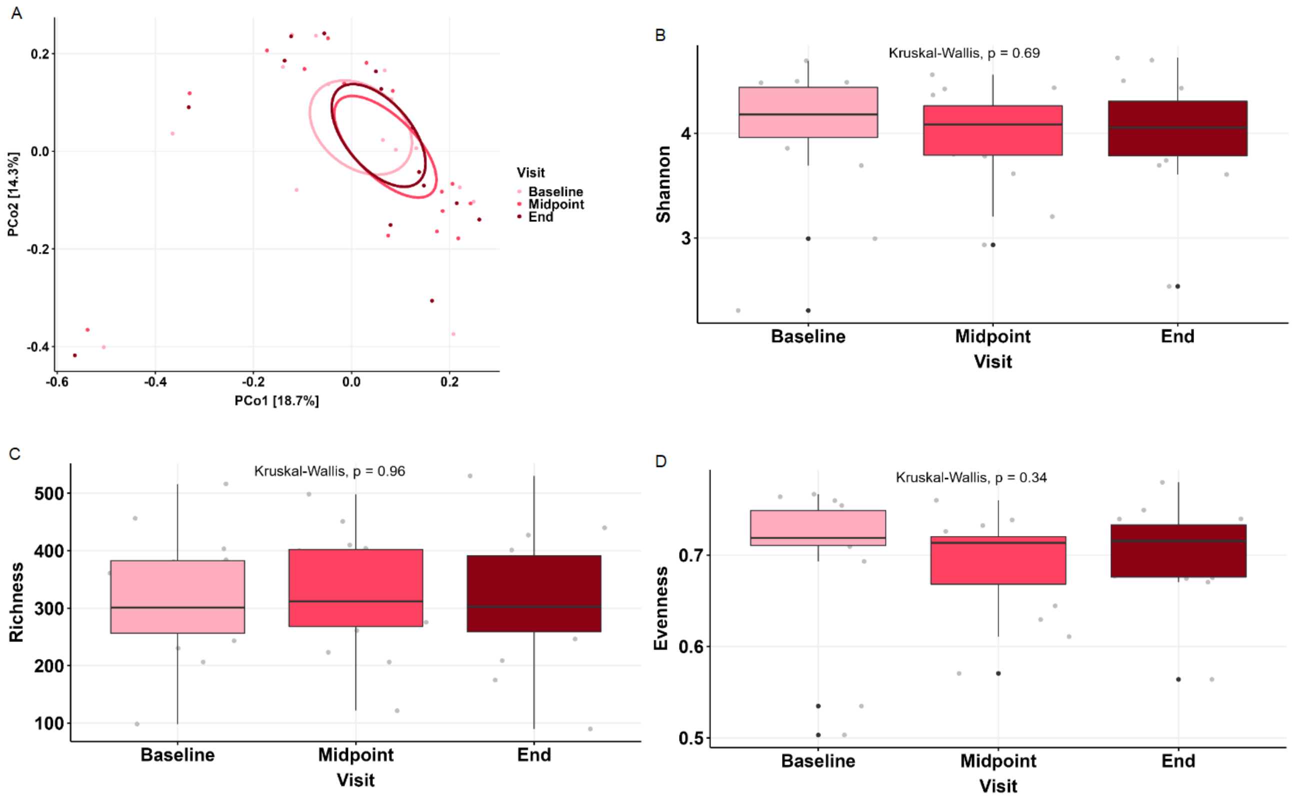

Fig. S4. The composition of the fecal gut microbiota at different time points upon the exercise program. (A) Bray-Curtis β-diversity; (B) Shannon α-diversity; (C) Richness; (D) Evenness of the gut microbiota.

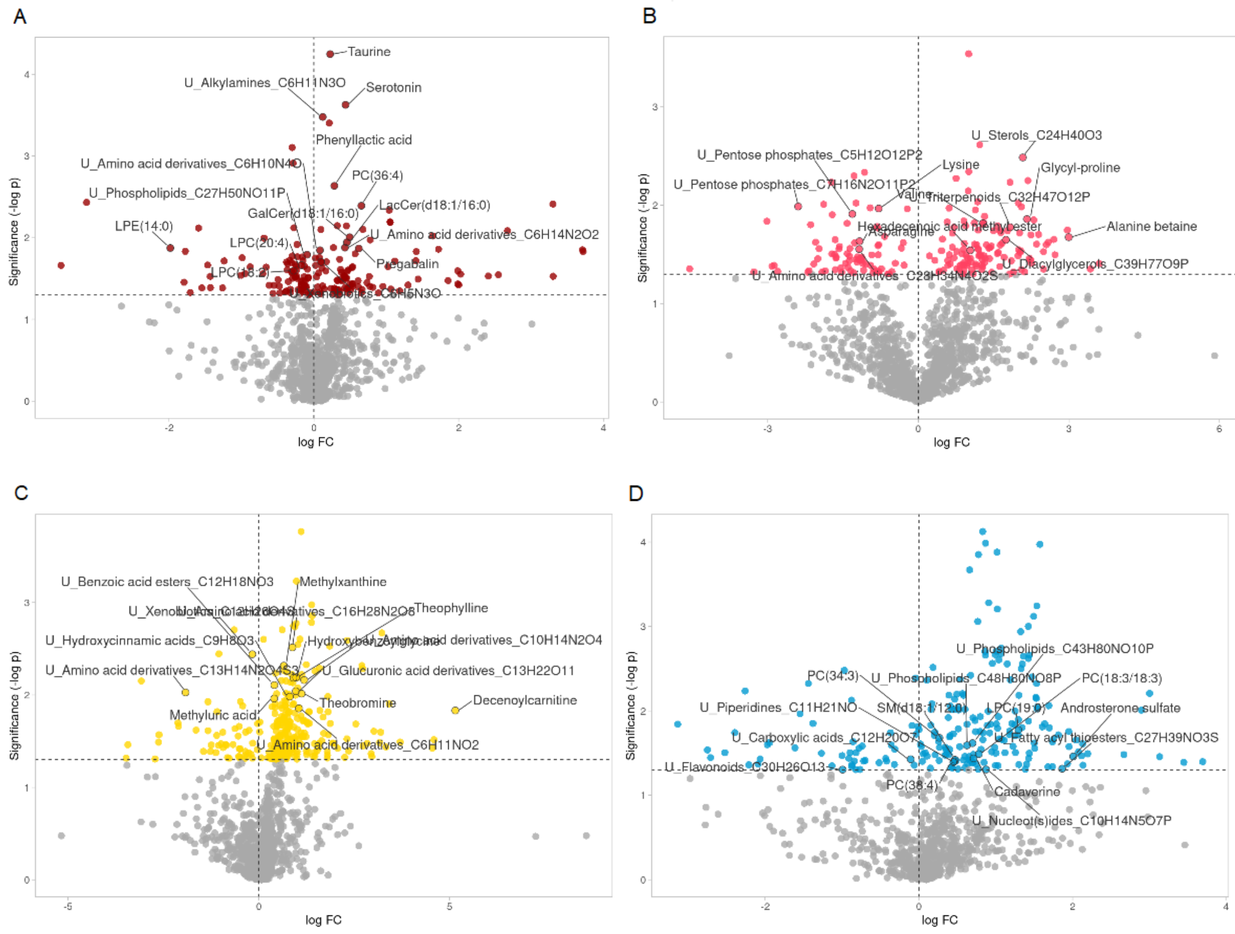

Fig. S5. Results from the paired T-test of metabolomics data per sample type. The x-axis represents the log fold change and the y-axis represents the negative log of the nominal p-value). (A) plasma; (B) stool; (C) urine; (D) adipose tissue.

Supplementary tables

Table S1. See excel file. Results from differential gene expression analysis on muscle, liver, and adipose tissue.

Table S2. See excel file. Spearman correlation results between the relative change of significantly differentially expressed genes (muscle, liver and adipose tissue) with the relative change of VO_2max/kg.

Table S3. See excel file. Spearman correlation results between the relative change significantly differentially expressed liver genes with the relative change of liver fat calculated as MRI-PDFF.

Table S4. See excel file. Spearman correlation results between the relative change significantly differentially expressed adipose tissue genes with the relative change of visceral fat volume.

Table S5. See excel file. Results from the differential abundance analysis of fecal microbial species between baseline and end of the intervention.

Table S6. See excel file. Results from paired Wilcoxon test of fecal microbial pathways.

Table S7. See excel file. Liquid chromatography-mass spectrometry (LC-MS) characteristics and statistical results from the paired t-test and linear mixed model of metabolites from plasma, stool, urine and adipose tissue. For the paired t-test, only features that were nominally significant are included in the table. For the linear mixed model, all features tested are included in the table. The sample type and average abundance based on linear mixed model results.

Table S8. See excel file. Spearman correlation results between the relative change of nominally significant metabolites from any sample type with the relative change of VO_2max/kg.

Table S9A. See excel file. Spearman correlation coefficients between the relative change of multi-omics outcomes.

Table S9B. See excel file. Spearman p-values between the relative change of multi-omics outcomes.

Table S10. See excel file. Procrustes results from baseline and end omics datasets.

Supplementary references

1. Tornvall G. Assessment of Physical Capabilities. Blackwell Scientific Publ. 1963;

2. Taylor HL, Jacobs DR, Schucker B, et al. A questionnaire for the assessment of leisure time physical activities. Journal of Chronic Diseases. 1978 Jan;31(12):741–55.

3. Hakola L, Savonen K, Komulainen P, et al. Moderators of Maintained Increase in Aerobic Exercise Among Aging Men and Women in a 4-Year Randomized Controlled Trial: The DR's EXTRA Study. Journal of physical activity & health. 2015 Nov;12(11):1477–84.

4. Babu AF, Csader S, Männistö V, et al. Effects of exercise on NAFLD using non-targeted metabolomics in adipose tissue, plasma, urine, and stool. Scientific reports. 2022;12(1):6485.

5. Kleiner DE, Brunt EM, Van Natta M, et al. Design and validation of a histological scoring system for nonalcoholic fatty liver disease. Hepatology. 2005 Jun;41(6):1313–21.

6. Bedossa P. Utility and appropriateness of the fatty liver inhibition of progression (FLIP) algorithm and steatosis, activity, and fibrosis (SAF) score in the evaluation of biopsies of nonalcoholic fatty liver disease. Hepatology. 2014 Aug;60(2):565–75.

7. Meijnikman AS, Davids M, Herrema H, et al. Microbiome-derived ethanol in nonalcoholic fatty liver disease. Nature medicine. 2022 Oct;28(10):2100–6.

8. Noerman S, Kokla M, Koistinen VM, et al. Associations of the serum metabolite profile with a healthy Nordic diet and risk of coronary artery disease. Clinical Nutrition. 2021 May;40(5):3250–62.

9. Tsugawa H, Cajka T, Kind T, et al. MS-DIAL: data-independent MS/MS deconvolution for comprehensive metabolome analysis. Nature methods. 2015 Jun;12(6):523–6.

10. Wishart DS, Tzur D, Knox C, et al. HMDB: the Human Metabolome Database. Nucleic acids research. 2007 Jan;35(Database issue):D521-6.

11. Smith CA, O'Maille G, Want EJ, et al. METLIN: a metabolite mass spectral database. Therapeutic drug monitoring. 2005 Dec;27(6):747–51.

12. Kim S, Thiessen PA, Bolton EE, et al. PubChem Substance and Compound databases. Nucleic acids research. 2016 Jan;44(D1):D1202-13.

13. Lipid Maps. Index [Internet]. Available from: https://www.lipidmaps.org/

14. Tsugawa H, Kind T, Nakabayashi R, et al. Hydrogen Rearrangement Rules: Computational MS/MS Fragmentation and Structure Elucidation Using MS-FINDER Software. Analytical chemistry. 2016;88(16):7946–58.

15. Mallick H, Rahnavard A, McIver LJ, et al. Multivariable association discovery in population-scale meta-omics studies. Coelho LP, editor. PLOS Computational Biology. 2021 Nov;17(11):e1009442.

16. Love MI, Huber W, Anders S. Moderated estimation of fold change and dispersion for RNA-seq data with DESeq2. Genome Biology. 2014 Dec;15(12):550.

17. Kuleshov M V., Jones MR, Rouillard AD, et al. Enrichr: a comprehensive gene set enrichment analysis web server 2016 update. Nucleic Acids Research. 2016 Jul;44(W1):W90–7.

18. Chen S, Zhou Y, Chen Y, et al. fastp: an ultra-fast all-in-one FASTQ preprocessor. Bioinformatics. 2018 Sep;34(17):i884–90.

19. Langmead B, Salzberg SL. Fast gapped-read alignment with Bowtie 2. Nature Methods. 2012 Apr;9(4):357–9.

20. Milanese A, Mende DR, Paoli L, et al. Microbial abundance, activity and population genomic profiling with mOTUs2. Nature Communications. 2019 Dec;10(1):1014.

21. Lin H, Peddada S Das. Analysis of compositions of microbiomes with bias correction. Nature Communications. 2020 Dec;11(1):3514.

22. Eldridge SM, Chan CL, Campbell MJ, et al. CONSORT 2010 statement: extension to randomised pilot and feasibility trials. BMJ (Clinical research ed). 2016 Oct;355:i5239.
