# Supplementary material for: Deep phenotyping of patients with MASLD upon high-intensity interval training
Source: JHEP Rep. 2024 Dec 16;7(3):101289. doi: 10.1016/j.jhepr.2024.101289 (PMC11883402; doi:10.1016/j.jhepr.2024.101289)
Supplement: Multimedia component 2 [file mmc2.docx]

**JHEP Reports**

**CTAT methods**

Tables for a “Complete, Transparent, Accurate and Timely account” (CTAT) are now mandatory for all revised submissions. The aim is to enhance the reproducibility of methods.

- Only include the parts relevant to your study
- Refer to the CTAT in the main text as ‘Supplementary CTAT Table’
- Do not add subheadings
- Add as many rows as needed to include all information
- Only include one item per row

**If the CTAT form is not relevant to your study, please outline the reasons why:**

|  |
| --- |

- 1. **Biological samples**

| **Description** | **Source** | **Identifier** |
| --- | --- | --- |
| Percutaneous ultrasound-guided liver biopsy samples obtained from the left liver lobe |  |  |
| Adipose tissue biopsies obtained from the superficial layer of the subcutaneous supraumbilical paramedian adipose tissue directly beneath the skin left side |  |  |
| Percutaneous ultrasound-guided muscle biopsies obtained from the left thigh *vastus lateralis* |  |  |
| Serum and plasma venous blood samples  obtained by venous puncture from a cephalic, basilica or median cubital vein of the left or right arm |  |  |
| 24-h urine samples |  |  |
| Morning stool samples |  |  |

- 1. **Deposited data**

| **Name of repository** | **Identifier** | **Link** |
| --- | --- | --- |
| EGA | EGAS00001006991 | [European Genome-phenome Archive (EGA)](https://web2.ega-archive.org/about/introduction) |

- 1. **Software**

| **Software name** | **Manufacturer** | **Version** |
| --- | --- | --- |
| R Statistical Computing Software | The R Foundation | https://www.r-project.org |
| Cosmed Omnia v. 2.0 | Cosmed Quark |  |
| Xcalibur software v. 4.3 | Thermo Fisher Scientific |  |
| MS-DIAL v. 4.90 | RIKEN Center for Sustainable Resource Science : Metabolome Informatics Research Team | http://prime.psc.riken.jp/compms/msdial/main.html |
| Trimmomatic v. 0.38 |  |  |
| Kallisto v. 0.45 |  |  |
| DESeq2 v. 1.34.0 |  |  |
| enrichR v. 3.1 |  |  |
| fastp v. 0.20.0 |  |  |
| Bowtie 2 v. 2.3.5 |  |  |
| mOTUs v. 3.0.1 |  |  |
| vegan v. 2.6-4 |  |  |
| chemometrics v. 1.4.2 |  |  |
| ANCOM-BC v. 1.4.0 |  |  |
| clustering v. 2.1.4 |  |  |
| fpc v. 2.2 |  |  |
| HUMANn3 |  |  |
| caret v. 6.0-93 |  |  |
| Maaslin2 v. 1.8.0 |  |  |
| python v. 3.7.3 |  |  |
| Conda v. 4.7.10. |  |  |

- 1. **Please provide the details of the corresponding methods author for the manuscript:**

| A.G. Holleboom |
| --- |

**2.0 Please confirm for randomised controlled trials all versions of the clinical protocol are included in the submission. These will be published online as supplementary information.**

|  |
| --- |
