# Supplementary material for: Deep phenotyping of patients with MASLD upon high-intensity interval training
Source: JHEP Rep. 2024 Dec 16;7(3):101289. doi: 10.1016/j.jhepr.2024.101289 (PMC11883402; doi:10.1016/j.jhepr.2024.101289)
Supplement: Multimedia component 5 [file mmc5.zip › Clinical Trial/C1 protocol CRISTINA versie 5.0 dd 8-7-2020 final.docx]

**An exerCise inteRventIon STudy In NAFLD pAtients (CRISTINA)**

**PROTOCOL TITLE** An exercise intervention study in NAFLD patients

| **Protocol ID** | CRISTINA-study |
| --- | --- |
| **Short title** | An exercise intervention study in NAFLD patients |
| **Version** | 5.0 |
| **Date** | 08-07-2020 |
| **Coordinating investigator/project leader** | Prof dr M. Nieuwdorp, MD PhD  Dept of Internal and Vascular medicine, AMC  Email: m.nieuwdorp@amc.uva.nl |
| **Principal investigator(s) (in Dutch: hoofdonderzoeker/ uitvoerder)** | Prof. dr. M. Nieuwdorp, MD PhD  Department of Vascular Medicine Amsterdam UMC, location AMC  Email: [m.nieuwdorp@amc.uva.nl](mailto:m.nieuwdorp@amc.uva.nl)  Prof dr A.K. Groen, professor of systems medicine  Department of Experimental Vascular Medicine  Department of Vascular Medicine Amsterdam UMC, location AMC  email: [a.k.groen@amc.uva.nl](mailto:a.k.groen@amc.uva.nl)  Dr. A.G. Holleboom, MD PhD  Department of Vascular Medicine Amsterdam UMC, location AMC  Email: [a.g.holleboom@amc.uva.nl](mailto:a.g.holleboom@amc.uva.nl)  V.A.T. Houttu, MSc, PhD student  Department of Vascular Medicine Amsterdam UMC  location AMC  Email: [v.a.houttu@amc.uva.nl](mailto:v.a.houttu@amc.uva.nl)  U. Boulund, MSc, PhD student  Department of Vascular Medicine Amsterdam UMC  Location AMC  Email: [u.boulund@amc.uva.nl](mailto:u.boulund@amc.uva.nl) |
| **Sponsor (in Dutch: verrichter/opdrachtgever)** | Dr. M. Nieuwdorp, MD PhD  Department of Vascular Medicine AMC-UvA  Email: m.nieuwdorp@amc.uva.nl |
| **Subsidising party** | H2020-MSCA-ITN-2018, EC grant agreement 813781 |
| **Independent expert (s)** | Dr. D.M. Cohn, department of Vascular Medicine Amsterdam UMC, location AMC  Email: d.m.cohn@amc.uva.nl |
| **Laboratory sites** | Laboratory of Experimental Vascular Medicine, AMC  (dr. G.M. Dallinga, email: [g.m.dallinga@amc.uva.nl](mailto:g.m.dallinga@amc.uva.nl))  Section of systems biology AMC and UVA (prof. A.K.  Groen and prof. N.A.W. van Riel)  **Novogene (UK) Company Limited** Building 250, Short Wing Ground Floor Babraham Research Campus Cambridge, CB22 3AT United Kingdom Tel: +44-1223-804-151 Email: [europe@novogene.com](mailto:europe@novogene.com)  **Afekta Technologies Ltd.**  Yliopistonranta 1L  70211 Kuopio  Finland  Tel: +358 44 243 2322  E-mail: support@afekta.com |

**PROTOCOL SIGNATURE SHEET**

| Name | Signature | Date |
| --- | --- | --- |
| **Head of Department:**  Prof. dr. J.M. Prins  Head of dept. of Internal Medicine |  |  |
| **Coordinating Investigator/Project leader/Principal Investigator:**  Prof. dr. M. Nieuwdorp  Internist-endocrinologist |  |  |

**TABLE OF CONTENTS**

[1 INTRODUCTION AND RATIONALE 12](#_Toc17374060)

[2 OBJECTIVES 18](#_Toc17374061)

[3 STUDY DESIGN 20](#_Toc17374062)

[4 STUDY POPULATION 21](#_Toc17374063)

[4.1 Population (base) 21](#_Toc17374064)

[4.2 Inclusion criteria 21](#_Toc17374065)

[4.3 Exclusion criteria 21](#_Toc17374066)

[4.4 Sample size calculation 22](#_Toc17374067)

[5 TREATMENTS OF SUBJECTS 23](#_Toc17374068)

[5.1 Treatment 23](#_Toc17374069)

[6 METHODS 25](#_Toc17374070)

[6.1 Study parameters/endpoints 25](#_Toc17374071)

[6.1.1 Main study parameter/endpoint 25](#_Toc17374072)

[6.1.2 Secondary study parameters/endpoints (if applicable) 25](#_Toc17374073)

[6.1.3 Other study parameters (if applicable) 25](#_Toc17374074)

[6.2 Randomisation, blinding and treatment allocation 26](#_Toc17374075)

[6.3 Study procedures 26](#_Toc17374076)

[6.3.1 Physical activity 26](#_Toc17374077)

[6.3.2 Dietary intake 26](#_Toc17374078)

[6.3.3 Anthropometrics 26](#_Toc17374079)

[6.3.4 Cardiorespiratory fitness assessment 26](#_Toc17374080)

[6.3.5 Resting energy expenditure 27](#_Toc17374081)

[6.3.6 Blood pressure and heart rate 27](#_Toc17374082)

[6.3.7 Magnetic Resonance Imaging 27](#_Toc17374083)

[6.3.8 Magnetic Resonance Spectroscopy 27](#_Toc17374084)

[6.3.9 Liver Transient Elastrography 27](#_Toc17374085)

[6.3.10 Urine and microbiota samples 28](#_Toc17374086)

[6.3.11 Blood samples 28](#_Toc17374087)

[6.3.12 Glucose monitoring 28](#_Toc17374088)

[6.3.13 Liver biopsies 28](#_Toc17374089)

[6.3.14 Muscle biopsies 29](#_Toc17374090)

[6.3.15 Subcutanous adipose tissue biopsies 29](#_Toc17374091)

[6.3.16 Storage for further analysis 29](#_Toc17374092)

[6.4 Withdrawal of individual subjects 29](#_Toc17374093)

[6.4.1 Specific criteria for withdrawal (if applicable) 30](#_Toc17374094)

[6.5 Replacement of individual subjects after withdrawal 30](#_Toc17374095)

[6.6 Follow-up of subjects withdrawn from treatment 30](#_Toc17374096)

[6.7 Premature termination of the study 30](#_Toc17374097)

[7 SAFETY REPORTING 31](#_Toc17374098)

[7.1 Temporary halt for reasons of subject safety 31](#_Toc17374099)

[7.2 AEs and SAEs 31](#_Toc17374100)

[7.2.1 Adverse events (AEs) 31](#_Toc17374101)

[7.2.2 Serious adverse events (SAEs) 31](#_Toc17374102)

[7.3 Follow-up of adverse events 32](#_Toc17374103)

[8 STATISTICAL ANALYSIS 33](#_Toc17374104)

[8.1 Primary study parameter(s) 33](#_Toc17374105)

[8.2 Secondary study parameter(s) 33](#_Toc17374106)

[9 ETHICAL CONSIDERATIONS 34](#_Toc17374107)

[9.1 Regulation statement 34](#_Toc17374108)

[9.2 Recruitment and consent 34](#_Toc17374109)

[9.3 Benefits and risks assessment, group relatedness 34](#_Toc17374110)

[9.4 Compensation for injury 35](#_Toc17374111)

[9.5 Incentives (if applicable) 36](#_Toc17374112)

[10 ADMINISTRATIVE ASPECTS, MONITORING AND PUBLICATION 37](#_Toc17374113)

[10.1 Handling and storage of data and documents 37](#_Toc17374114)

[10.2 Monitoring and Quality Assurance 37](#_Toc17374115)

[10.3 Amendments 37](#_Toc17374116)

[10.4 Annual progress report 37](#_Toc17374117)

[10.5 Temporary halt and (prematurely) end of study report 37](#_Toc17374118)

[10.6 Public disclosure and publication policy 38](#_Toc17374119)

[11 REFERENCES 39](#_Toc17374120)

**LIST OF ABBREVIATIONS AND RELEVANT DEFINITIONS**

| **ABR** | **General Assessment and Registration form (ABR form), the application form that is required for submission to the accredited Ethics Committee; in Dutch: Algemeen Beoordelings- en Registratieformulier (ABR-formulier)** |
| --- | --- |
| **AE** | **Adverse Event** |
| **ANCOVA** | **Analysis of Covariance** |
| **ANOVA** | **Analysis of Variance** |
| **CA** | **Competent Authority** |
| **CCMO** | **Central Committee on Research Involving Human Subjects; in Dutch: Centrale Commissie Mensgebonden Onderzoek** |
| **DSMB**  **ECG** | **Data Safety Monitoring Board**  **Electrocardiography** |
| **EASD** | **European Association for the Study of Diabetes** |
| **EASL** | **European Association for the Study of the Liver** |
| **EASO** | **European Association for the Study of Obesity** |
| **EU** | **European Union** |
| **EudraC** | **European drug regulatory affairs Clinical Trials** |
| **GCP** | **Good Clinical Practice** |
| **GDPR** | **General Data Protection Regulation; in Dutch: Algemene Verordening Gegevensbescherming (AVG)** |
| **HCC** | **Hepatocellular carcinoma** |
| **IB** | **Investigator’s Brochure** |
| **IC** | **Informed Consent** |
| **METC**  **MRI**  **MRS**  **NAFL**  **NAFLD**  **NASH**  **REE** | **Medical research ethics committee (MREC); in Dutch: medisch-ethische toetsingscommissie (METC)**  **Magnetic Resonance Imaging**  **Magnetic Resonance Spectroscopy**  **Non-alcoholic Fatty Liver**  **Non-alcoholic Fatty Liver Disease**  **Non-alcoholic Steatohepatitis**  **Resting Energy Expenditure** |
| **(S)AE**  **SIBO** | **(Serious) Adverse Event**  **Small Intestinal Bacterial Overgrowth** |
| **Sponsor** | **The sponsor is the party that commissions the organisation or performance of the research, for example a pharmaceutical company, academic hospital, scientific organisation or investigator. A party that provides funding for a study but does not commission it is not regarded as the sponsor, but referred to as a subsidising party.** |
| **WMO** | **Medical Research Involving Human Subjects Act; in Dutch: Wet Medisch-wetenschappelijk Onderzoek met Mensen** |

**SUMMARY**

**Rationale:** Non-Alcoholic Fatty Liver Disease (NAFLD) is the most common cause of liver dysfunction with a global prevalence of 25 % which is accompanied by increasing obesity and obesity related metabolic diseases. NAFLD is defined as at least 5 % of fat in hepatocytes in absence of other hepatopathies. It covers a spectrum of different disease stages varying from non-alcoholic fatty liver disease (NAFL) to an advanced stage of the disease called non-alcoholic steatohepatitis (NASH). NASH is defined as steatosis with hepatocyte injury, inflammation and fibrosis while in NAFL only lipid accumulation is detected. Hepatocellular carcinoma (HCC) can develop at the fibrotic stage before any sign of symptoms of liver dysfunction. The end stage of the disease is cirrhosis which is an increasing cause of liver transplantations in the western world. There are currently no pharmacological treatment options to treat NAFLD or to prevent its progression to NASH, NAFL-related fibrosis or cirrhosis. Internationally recommended therapies include lifestyle intervention modifications targeting weight loss. Nevertheless, lifestyle interventions require a lot of resources. Additionally, weight loss as a clinical intervention is rather unsustainable. Exercise has been demonstrated to reduce (MRI based) hepatic lipid content in NAFLD patients without overall weight loss. However, the mechanism behind the effect of exercise independently of body weight change is unclear. There is a strong link between gut microbiota and the development of NAFLD. Additionally, exercise modulates gut microbiota. Thus, it can be suggested that the effects of exercise on NAFLD may not only be mediated through a physiological response to the liver, but through the gut microbiota and related metabolites. This study aims to investigate the clinical effect of exercise on molecular mechanisms involved in NAFLD and explore both interaction and relative contribution of liver, subcutaneous adipose tissue and muscle histology and gene expression in relation to glucose metabolism and gut microbiome composition. In addition to this we will study whether potential changes in the gut microbiota can be used to monitor exercise progression and predict the prognosis of NAFLD. Finally, potential biomarkers will also be searched for by transcriptomic analyses of liver, muscle and adipose tissue, and metabolomic analyses of blood, feces and urine.

**Objective**: We aim to study the effect of exercise on NAFLD (by analysing hepatic lipid content, liver histology, and gene expression). Furthermore, we aim to establish the role of exercise-mediated changes in gut microbiome in the reduction of severity of NAFLD and the relation with other metabolically involved organs including adipose tissue and muscle metabolism and glucose excursions. We thus aim to identify biomarkers of exercise effect on NAFLD state. These biomarkers comprise plasma and urine metabolites, gene expression of liver, muscle and adipose tissue, as well as gut microbial composition.

**Study design:** Intervention study.

**Study population:** In total 15 subjects aged 18-70 years of age with diagnosed NAFLD.

**Intervention (if applicable)**: High-intensity interval exercise training for 12 weeks.

**Main study parameters/endpoints:** The main study parameters are changes in NAFLD liver histology and gene expression in relation to oral and gut microbiota composition changes upon exercise. Second changes in other organs involved upon exercise driven metabolic fitness including subcutaneous adipose tissue and muscle gene expression (RNA-sequencing) and histology, in relation to glucose metabolism (freestyle libre) and metabolites in plasma and urine will be studied. Finally, the relative contribution to NAFLD and glucose metabolism of the different organs (liver, adipose tissue and muscle) upon exercise will be studied.

**Nature and extent of the burden and risks associated with participation, benefit and group relatedness:**

This study is a continuation of our previous intervention studies aiming to study underlying mechanisms of gut microbiota in liver gene expression in NALFD-NASH subjects (MASH study 2013_207) and our study on the role of microbiota on muscle gene expression and glucose metabolism (APPETITE study 2014_084). Oral microbiota is studied by taking an oral swab of the upper front teeth rime in overnight fasted subjects that refrained from tooth brushing that morning (FATMED study 2016_117). Moreover, we currently are building a large prospective cohort of patients with NAFLD-NASH to follow the natural course of (liver biopsy proven) changes in NAFLD-NASH between baseline and after 5 years (2017_311). Participants will be recruited from our outpatient clinics. An ultrasound or transient electrography will be performed to establish hepatic steatosis, if they do not already have an existing diagnosis. All subjects will in total have six site study visits, and total 21 hours in 12 weeks. At each visit we will collect blood and fecal samples. In addition, body weight will be measured at each visit. Other anthropometrics including height and waist circumference will be screened at baseline. At the baseline (first) and endpoint (final) visit we will collect (ultrasound guided) liver, muscle and subcutaneous adipose tissue biopsies, and Magnetic Resonance Imaging (MRI) and Magnetic Resonance Spectroscopy (MRS) will be performed. During these two visits, bioelectrical impedance will be conducted to screen body composition and resting energy expenditure (REE) will be determined. Also, ergospirometry test will be performed to determine the intensity of the individually prescribed training program and the efficacy of the exercise intervention. Exercise habits will be assessed by a questionnaire for the entire 12-week intervention period. Additionally, an objective physical activity monitoring will be performed through the intervention. Subjects will also fill in online questionnaires regarding their food intake for one week prior to each visit. Glucose monitoring will be conducted for 4 weeks during the intervention, 2 weeks at the baseline and 2 weeks before the end of the intervention to screen the effect of the exercise on glucose metabolism.

Our study participants will be referred to the AMC intervention radiology department for liver biopsy to assess NAFLD histology severity and gene expression (RNA sequencing) in liver tissue. As an experienced interventional radiologist will perform the liver biopsy ultrasound-guided, the risk (comprising mostly bleeding from the biopsy site) of complications will be very low (<0.1 %). Moreover, local hemostasis after the procedure will be observed and bleeding disorders or anticoagulant therapy are an exclusion criterion. Based on our previous experience, we might encounter ~ 1 patients with a complication (most likely small bleeding) in our entire study. Despite ample efforts to establish non-invasive methods in small groups of NAFLD/NASH patients over the last decade, a liver biopsy is still the gold standard for discerning between NAFLD and NASH and provides possibility to study gene expression. NASH refers to an advanced state of NAFLD which is characterised by hepatocyte injury, inflammation and fibrosis. When participants are found to have NASH in their baseline liver biopsy, they will be referred to our outpatient clinic for clinical follow-up and dietary counselling.

# INTRODUCTION AND RATIONALE

Non-Alcoholic Fatty Liver Disease (NAFLD) is the most common cause of liver dysfunction in the western world. It is strongly associated with obesity, insulin resistance, hypertension, and dyslipidemia and, as such, can be regarded as the hepatic component of the metabolic syndrome (de Alwis & Day, 2008; Younossi, 2019; Younossi et al., 2016). The current global prevalence of NAFLD has been estimated at 25 % of the adult population, however since obesity is reaching epidemic proportions worldwide, NAFLD is also expected to further increase in prevalence (Ekstedt et al., 2006; Younossi et al., 2016). Ethnic minorities show different predisposition to NAFLD. E.g. in the United States,  Hispanics predisposes to progressive NAFLD, whereas African background may be protective (Rich et al, 2018). Strikingly, to date no approved pharmacotherapy exists for NAFLD. NAFLD is attributable to high medical costs in society, which highlights the need for viable treatment options (Younossi, 2019).

NAFLD is defined as at least 5 % of hepatocytes presenting with steatosis (lipid accumulation), in absence of other hepatopathies, most importantly viral hepatitis, excessive alcohol consumption, or auto-immune or genetic liver diseases (Younossi et al., 2016). It is typically an asymptomatic illness, which explains why it in many cases can reach advanced stages before it is diagnosed. The diagnosis is typically made after routine health checks which identifies liver blood test abnormalities (NAFLD accounts for 60-90 % of such test results), however 80 % of patients with NAFLD have normal liver blood tests (de Alwis & Day, 2008). There appears to be no increase in mortality risk in patients with steatosis, which is often seen as rather benign, however patients with simple steatosis do suffer an increased risk of cardiovascular but not liver-related mortality (Bedossa, 2017; Ekstedt et al., 2006). The exact link between NAFLD and cardiovascular disease has not yet been clearly described (de Alwis & Day, 2008; Younossi et al., 2016). Moreover, longitudinal research indicates that most patients with NAFLD develop type 2 diabetes or impaired glucose tolerance over time due to increased adiposity, hence the collection of adipose tissue biopsies in this study (Ekstedt et al., 2006).

NAFLD covers a spectrum of liver diseases, ranging from simple steatosis, or NAFL, to its more progressive forms Non-Alcoholic Steatohepatitis (NASH), fibrosis, cirrhosis and hepatocellular carcinoma (HCC). Importantly, HCC can occur in the fibrotic state, i.e. before cirrhosis develops, indicating the toxic metabolic environment in NASH and setting NASH-HCC apart from e.g. HCC induced by viral hepatitis, which only occurs in the cirrhotic state (Bedossa, 2017; Friedman et al., 2018). NASH is defined as steatosis with hepatocyte injury, inflammation and fibrosis. In 10 years’ time, approximately 15 % of patients with simple steatosis will develop NASH (Chedid, 2017). Exact data for the Dutch population are lacking, but a study by Koehler et al. (2016) found presence of fibrosis assessed by transient elastography (TE) in 5.6 % of the general population over 45 years of age. It was associated with type 2 diabetes and steatosis. Currently the only possible method to discern between steatosis and steatohepatitis, and thus to determine the stage of NAFLD or NASH, is by histological examination which requires a liver biopsy. This is of high importance, since different stages have different prognoses and thus must be treated or managed differently (Bedossa, 2017; de Alwis & Day, 2008). Cirrhosis caused by NASH has become the most common cause of liver transplantations in the USA in 2018 (Younossi et al., 2016). Most patients have recurring steatosis within 4 years of a liver transplant, with 50% of that group progressing to NASH and fibrosis, and cases of recurrent cirrhosis have also been reported (de Alwis & Day, 2008; Gitto et al., 2018).

There are currently no pharmacological treatment options to treat NAFLD or to prevent its progression to NASH, NAFL-related fibrosis, or cirrhosis (Oseini & Sanyal, 2017; Ratziu, 2017). According to the European Association for the Study of the Liver (EASL), European Association for the Study of Diabetes (EASD), and the European Association for the Study of Obesity (EASO) (2016) the internationally recommended therapies include physical exercise and dietary modifications. Mainly, weight reduction through lifestyle interventions is the target to prevent NAFLD progression. It is stated in the guideline that 7-10 % weight reduction is recommended for NAFLD patients. Previously published intervention studies have investigated the effect of weight reduction through a combination of diet, exercise and behavioural interventions on the disease (Promrat et al., 2010; Vilar-Gomez et al., 2015). Results are still inconsistent whether weight loss is sufficient therapy in advanced stages of NASH whereas in steatosis even a small weight loss is beneficial for reducing liver fat and insulin resistance (Ratziu, 2017). However, weight reduction and its sustainability are more complicated in real-life clinical practice than seen in research intervention trials with intense guidance and follow-ups. Most of the obese patients who manage to lose weight successfully tend to regain most of it in the first 3 years. In addition to the difficulties related to weight management in health care, lifestyle interventions require a lot of investment and knowledge among health care professionals. Due to these matters it would be necessary to identify which specific lifestyle intervention would be the most effective to prevent the progression of NAFLD to increase efficacy, adherence and motivation.

Despite the fact that weight reduction is beneficial for the liver status, it has been indicated that hepatic fat can be reduced even without significant overall weight loss through lifestyle interventions (Katsagoni et al., 2017). This further confirms that lifestyle interventions are beneficial for NAFLD without directly targeting weight reduction. Indeed, physical exercise as a lifestyle intervention is seen to reduce liver fat without change in overall body weight (Keating et al., 2015). In longitudinal studies it has been shown that regular exercise minimizes the risk for developing NAFLD, and cardiorespiratory fitness is associated with changes in liver fat (Anderson et al., 2016; Kantartzis et al., 2009). In general, physical activity is a well-known lifestyle factor that influences overall health. Various epidemiological studies have shown the association between sedentary lifestyle and increased risk of diseases and mortality (Patterson et al., 2018). It is recommended for an adult to perform regularly either at least 150 minutes a week moderate-intensity or 75 minutes a week vigorous-intensity training or an appropriate combination of moderate- and vigorous-intensity aerobic activity with muscle-strengthening exercise for at least 2 times a week (US Department of Health and Human Services, 2018)

There are only a few randomized clinical intervention studies investigating the effect of physical exercise on NAFLD in humans all based on surrogate endpoints. In a 12-week randomized controlled trial with NAFLD subjects high-intensity aerobic interval (HII) training reduced (Mri based) liver fat and improved liver enzymes independent of weight change with decrease in fat mass (Hallsworth et al., 2015). Studies investigation aerobic exercise have found similar results with different duration of aerobic exercise intervention from 4 weeks up to 16 weeks (Johnson et al., 2009; Keating et al., 2015; Sullivan et al., 2011). Additionally, in a randomized clinical trial with 8-week intervention of resistance exercise (MRI based) liver fat was reduced without weight loss or change in body composition (Hallsworth et al., 2011). Another resistance exercise intervention with 12-week intervention (MRI based) liver fat content and body weight were reduced but there was no correlation between these parameters suggesting the improvement was not due to weight loss (Zelber-Sagi et al., 2014). After aerobic exercise levels of liver enzymes ALT and AST were decreased (Shamsoddini et al, 2016). Also, resistant training lowered the levels of these enzymes (Hajighasem et al., 2018; Zelber-Sagi et al., 2014). Although exercise is shown to reduce (MRI based) hepatic lipids in NAFLD patients, Houghton et al. (2017) demonstrated in a 12-week randomized clinical exercise trial that exercise does not have an influence on inflammatory markers or fibrosis in NASH patients. Altogether, although total training time, duration of sessions, frequency and exercise method vary greatly in previously published studies, for a precise conclusion it can be suggested that regardless of the type of the exercise intervention exercise alone is an effective method for reducing liver fat content either with or without change in body weight or body composition. However the organ specific regulation of each organ involved in metabolic fitness (including muscle, adipose tissue and liver) has not been studied at a molecular (biopsy) gene expression and histology level upon strenuous exercise in NAFLD subjects.

This is especially important as the liver seems to be the central orchestrator affected by exercise due to the major roles of the liver in metabolism. However, the mechanism behind the effect of exercise independent of body weight on hepatic fat is still unknown (Farzanegi et al., 2019). In general there is variation of the physiological response to exercise between different types of exercise. It is demonstrated that physiological response to exercise is seen in metabolism of lipids in adipose and skeletal muscle tissues. One reason for reduced lipids in the liver after regular exercise can be due to increased lipolysis and decreased lipogenesis (Trefts et al., 2015). It is also suggested that exercise has an effect on the inflammation due to increased lipid oxidation and then decreased lipid accumulation in liver mediated through exercise (Farzanegi et al., 2019; Trefts et al., 2015). However, the level of triglycerides and free fatty acids can be lowered only with weight decrease and that is why decrease in hepatic lipids without weight loss is not due to the transportation of lipids to the liver. Furthermore, exercise has a beneficial effect on insulin sensitivity in skeletal muscle and adipose tissues and also affects to glucose metabolism in the liver. Thus, it can be suggested that the effects of exercise on NAFLD is partly caused by the physiological response to exercise. However, performing a systems biology approach to dissect molecular pathways underlying the effect of exercise on hepatic lipids absent of weight loss in relation to other organs involved in metabolic fitness (muscle and adipose tissue) in NAFLD patients warrants more studies.

Previous studies have indicated alterations in the gut microbiota in patients with NAFLD, typically an overgrowth of bacteria in the small intestine, referred to as small intestinal bacterial overgrowth (SIBO). In addition to this NAFLD is associated with increased gut permeability, which correlates with the severity of steatosis (Miele et al., 2009; Wigg et al., 2001). Previous research has identified a causal relationship between NASH and the gut microbiota (Fischer et al., 2010). Changes in the gut microbiota can lead to worsening of steatosis and steatohepatitis in NASH mouse models, and these changes could be transmitted to wildtype mice (Henao-Mejia et al., 2012). In addition to this two observational studies found an increased abundance of Gammaproteobacteria in obese NASH patients compared to healthy controls, and a lower abundance of Bacteroidetes in subjects with NASH compared to subjects with steatosis (Mouzaki et al., 2013; Raman et al., 2013). Changes in gut microbial composition can be used to predict the development of choline deficiency-induced NAFLD, indicating the usefulness of this measure (Fischer et al., 2010).

Perhaps, these findings can be implemented in the relation to the effects of exercise, whether the mechanisms behind the effect of exercise on NAFLD is mediated through gut microbiota and related metabolites. The effects of exercise on gut microbiota have mainly been investigated in rodent models. Among human studies, a recent longitudinal study with obese and lean subjects tried to demonstrate the effect of exercise intervention on the modification of gut microbiota in obese and lean subjects (Allen et al., 2018). Another follow-up study with obese women showed also alterations in microbiota composition after endurance exercise intervention (Munukka et al., 2018). One recently published randomized crossover trial study with 33 subjects investigated whether a 5-week exercise intervention alters gut microbiota and how it is associated with the cardiometabolic phenotypes in elderly men (Taniguchi et al., 2018). In the exercise period compared to control period a significant decrease in the relative abundance of *Clostridium difficile* and a significant increase in *Oscillospira* were detected. The abundance of *Clostridium difficile* was correlated with AST, ALT among several other metabolic markers whereas *Oscillospira* was correlated with body weight, total cholesterol, HDL and HbA1c. In a mice model exercise-caused alterations in gut microbiota in donor mice were detected in recipient mice after five weeks of a colonization (Allen et al., 2018). The role of physical exercise on gut microbiota needs more randomized controlled trials for better understanding of the effects of exercise on gut microbiota.

Gut microbiome is strongly affected by the host including host’s genetics and ethnicity, but also it is affected by daily fluctuation of gut motility and transit time (Deschasaux, et al., 2018; Roager et al., 2016; Tottey et al., 2017). Strong inverse correlation is seen with defaecation patterns where increased stool frequency is correlated with decreased diversity (Hadizadeh et al., 2017). These changes might play a role also further in the metabolism of fermented carbohydrates and catabolised proteins in the colon affecting the whole body (Roager et al., 2016).  Furthermore, exercise has effect on gastric emptying (Horner, Schubert, Desbrow, Byrne, & King, 2015). The changes in defaecation patterns might be one of the explanatory factors of changes in the gut microbiota and metabolomes (Roager et al., 2016). Thus, the effects of gut motility require more studies to be able to address the underlying modulating factors upon interventions such as exercise interventions. Small bowel motility can be assessed by non-invasive dynamic MRI motility imaging which requires only a short imaging time (C. S. de Jonge, Smout, Nederveen, & Stoker, 2018; Catharina S de Jonge et al., 2018). As far as we know gut motility has not been previously studied in NAFLD patients upon exercise which might be essential when interpreting the effects of exercise on gut microbiome.

It has been estimated that gut bacteria in humans produce about 300 volatile organic compounds, and these bacterial products may be continuously delivered to the liver through the portal vein. The effect of this is largely unknown, however a previous study found that the role of gut microbiota in affecting metabolic pathways was important in cardiovascular disease (Knaapen et al., 2013). There have also been previous studies investigating the NAFLD specific metabolome. There are certain metabolic compounds found in feces that correlate with variations in the microbiome, specifically for NAFLD patients compared to lean controls (Raman et al., 2013) Previous studies have found that the liver and plasma metabolic profile in NAFLD correlates well, and plasma metabolomic profiling has been used to identify three distinct NAFLD patient subtypes (Alonso et al., 2017). In addition to this, several different studies have identified a very clear distinction in the NAFLD metabolome compared to healthy controls, some of these effects may be used to predict hepatic fat accumulation. Despite recent advances, reliable biomarkers are needed as an early diagnostic measure to be used by clinicians (Gitto et al., 2018).

The transcriptomic profile of adipose tissue in NAFLD is currently in the very early stages of being investigated. One study investigated adipose tissue transcriptome in adolescents, and found several uniquely regulated networks in normal, steatosis, and NASH samples (Sheldon et al., 2016) This suggests an important effect on the adipose transcriptome in NAFLD, however the driving forces of these effects are largely unknown. It is likely that the mechanisms can be further elucidated by investigating which genes vary upon HI exercise.

A few recent studies investigated the transcriptome in NAFLD liver samples, they identified several genes associated with cirrhosis and alcohol metabolism, and suggested potential functional pathways and networks that were affected during different stages of NAFLD. They found some pathway perturbations in both NAFL and NASH that were associated with lipid metabolism and cell cycle control (Almanza et al., 2018; Gerhard et al., 2018; Suppli et al., 2019; Ye & Liu, 2015; Zhu et al., 2016) Even specific sets of genes have been identified in subgroups of NAFLD that can aid in discerning between high- and low-risk NAFLD related mortality (Lou et al., 2017; Moylan et al., 2014; Starmann et al., 2012) Moreover several studies have been conducted to analyse the transcriptomic effect of diet on the NAFLD liver, however the effect of exercise on hepatic gene expression is largely unknown (de Wit, Afman, Mensink, & Müller, 2012; Sales et al., 2018). The gene expression changes in muscle tissue following exercise intervention in NAFLD patients has not been analysed previously, though it is known that exercise does affect the muscle transcriptome (Damas et al., 2018; Hinkley et al,2017; Keller et al., 2011; Lundberg et al., 2016). This intervention study and the analysis of muscle transcriptome can thus be of great importance in understanding the underlying molecular mechanisms of the exercise effect on NAFLD. Previous exercise intervention studies have found an effect on NAFLD, however there is scant evidence of the underlying mechanisms of this effect. In addition to this, there are scarce clinical studies that have investigated the effect of exercise on the microbiome in relation to NAFLD (Porras et al., 2018). This study will investigate the clinical effects of high intensity exercise on NAFLD, and explore whether these effects are related to changes in gut microbiota composition. In addition to this we will study whether potential changes in the gut microbiota can be used to monitor exercise progression and predict the prognosis of NAFLD. Finally, potential biomarkers will also be searched for by transcriptomic analyses of muscle, adipose, and liver tissue, and metabolomics analyses of blood and urine.

# OBJECTIVES

**Primary Objective:** to evaluate the clinical effect of high-intensity interval exercise on NAFLD liver histology and genes in relation to gut microbiome composition. To do this we will collect:

At baseline and endpoint:

- Anthropometrics (body weight, waist and hip circumference and body composition)
- Ergospirometry tests (VO2max)
- MRI and MRS scan of the abdomen to estimate liver fat content, fibrosis and abdominal fat depot assessed by MR modality, and also the liver fibrosis assessed by transient elastrography (FibroScan)
- Oral swab, Fecal and 24 h urine samples
- Free style Libre glucose monitoring (worn during 2 weeks)
- Blood samples (fasting plasma and DNA)
- (Ultra sound guided) Liver biopsy, muscle and adipose tissue biopsy for histology and gene expression
- REE
- Calf circumference

At mid-intervention:

- Body weight, waist and hip circumference
- Oral and fecal sample
- Blood sample (fasting)

At baseline:

- Height
- Ethnicity

At baseline, mid-intervention and endpoint:

- 4-day dietary intake list before the intervention and 5-day dietary lists before mid-intervention and endpoint

Through the entire intervention

- Physical activity questionnaire
- Objective physical activity monitoring

**Secondary Objective:** to analyse gut microbiota composition and identify novel biomarkers of high-intensity interval exercise effect on NAFLD liver genes progression. Also, we aim to study the relative contribution of each organ (liver/muscle and adipose tissue) in relation to gut microbiota composition and improvement in glucose metabolism upon exercise. To do this we will collect:

At baseline and endpoint:

- Fecal and 24 h urine samples
- Blood samples
- Liver, muscle and adipose tissue biopsy (for histology and gene expression)
- Gut motility

At mid-intervention:

- Fecal samples
- Blood samples

# STUDY DESIGN

This study is designed as an intervention study in which subjects will follow the 12-week exercise intervention. Subjects will receive instructions for a high-intensity aerobic interval training (HI) (described below). Dietary intake will remain unchanged. The first part of the study is conducted with 15 subjects. This is followed by an interim analysis and based on the results of this analysis, the intervention will be extended with another 15 subjects based on approval by the IRB. The study design is presented in figure 1.

Complementary study

Baseline

Mid-intervention

Final intervention

Interim analysis

Diagnosed NAFLD

(5.5 % > liver fat)

BMI < 40 kg/m^2^

18–70 years of age

n=15

Ergospirometry test

Anthropometrics

Food records

REE

Oral swab

Stool and urine samples

MRI and MRS

Blood samples

Liver, adipose tissue and muscle biopsies

Ergospirometry test

Anthropometrics

Food records

REE

Oral swab

Stool and urine samples

MRI and MRS

Blood samples

Liver, adipose tissue and muscle biopsies

Body weight

Food records

Oral swab

Stool samples

Blood samples

Exercise intervention – pilot study

Screening

Week 0

+/- 3 week

Identical to pilot study,

recruitment of

additional subjects

+/- 3 months

Continous glucose monitoring

Continous glucose monitoring

Week 12

+/- 3 week

Week 6

+/- 2 week

Figure 1 Overview of the study design with the main procedures that will be performed during the 12-week exercise intervention.

# STUDY POPULATION

## Population (base)

The 18-70-year-old subjects (n=15) will be recruited via advertisements from the outpatient clinics of the Amsterdam Medical Centre (AMC), and through newspaper advertisement.

## Inclusion criteria

In order to be eligible to participate in this study, a subject must meet all of the following criteria:

- Diagnosis of steatosis on transient elastography, conventional ultrasound, magnetic resonance imaging, computed tomography, liver biopsy, or identified as steatotic subject by fatty liver index (FLI) algorithm which is based on waist circumference, body mass index, and levels of triglyceride and γ-glutamyltransferase (Koehler et al. 2013).
- 18-70 years of age
- BMI < 40 kg/m^2^

## Exclusion criteria

A potential subject who meets any of the following criteria will be excluded from participation in this study:

- Acute illness or current evidence of acute or chronic inflammatory or infective diseases
- Abusive alcohol use (> 21 IU/week for men, > 14 IU/week for women)
- Diagnosed liver cirrhosis and/or hepatocellular carcinoma
- Diagnosed cardiorespiratory, neurological or musculoskeletal disease
- Diagnosed insulin or GLP-1 agonist treated type 2 diabetes or type 1 diabetes
- Hepatitis B and/or C or auto-immune hepatitis
- Wilsons disease/alpha-1-antitripsine deficiency
- Hemochromatosis
- Untreated hypothyroidism
- Lipoatrophy
- Bleeding disorder or anti-coagulant use which cannot be temporarily discontinued
- Not able or willing to undergo MRI (for example claustrophobia, ICD, pacemaker)
- Diagnosed depression or any mental illness rendering the patients unable to understand the nature, scope and possible sequences of the study
- Any participation in regular exercise and/or diet program more than 2 times per week in the 3 months prior to recruitment

## Sample size calculation

Based on hepatic PNPLA3 expression that is twice as high in steatosis patients compared to healthy obese subjects (Aragonès et al., 2016). To detect a 25% reduction in PNPLA2 expression upon 12 weeks exercise course, we expected to need a group sample size of 28 (2sided chi- square test with a desired alpha of 0.05 and a desired power of 0.8). Assuming around 10 % of dropouts in this trial, a final sample size of 30 is required. Based on the suggestions of the IRB we will start with inclusion of 15 subjects and study changes in liver gene RNAseq including PNPLA3 gene liver expression upon exercise (Aragonès et al., 2016). Based on those findings, we will validate our power calculation above and see if the number of 30 subjects is indeed needed.

# TREATMENTS OF SUBJECTS

## Treatment

The intervention group will follow a high-intensity interval training (HI) protocol on a cycle ergometer (Hallsworth et al., 2015). To determine the intensity of the individually prescribed training program and the efficacy of the exercise intervention, ergospirometry tests will be performed in collaboration with the department of Cardiology AMC (dr. Jorstad) that has all expertise to execute this study. The training sessions and ergospirometry tests will be carried out in Polifysiek, Hogeschool van Amsterdam. Furthermore, physical activity of study subjects will be monitored through the intervention by questionnaires. These are described later in the chapter of study procedures. Subjects are asked to keep their diet and weight unchanged.

The exercise intervention will start immediately after the ergospirometry test. In detail, the concept of HI involves repeated bouts of exercise at an intensity of 85 % of W_max4_ interspersed by recovery periods based on a baseline ergospirometry test. W_max4_ refers to the hypothetical workload sustainable for 4 min (Tornvall G, 1963). While being hypothetical, W_max4_ is useful for defining the presumably optimal intensity for work intervals at HI. The intervention group will perform HI session involving 5 bouts of 2-4 minutes work intervals (at 85 % of W_max4_) interspersed by 3 minutes of active recovery (at 10 % of W_max4_) period twice per week on non-consecutive days for 12 weeks. Each work interval will be 2 minutes long in the first week with 5 seconds added per each exercise session (i.e. 10 seconds per week) so that HI intervals will be 4 minutes long by week 12. If the subject fails to complete the work interval, he/she will still be instructed to continue the exercise session by starting the following scheduled work interval(s) after the recovery period(s). At the beginning of the intervention period, each HI session will last approximately 40 minutes, including warm-up (at 30% of W_max4_), recovery periods and cool-down (at 20 % of W_max4_). At the end of the intervention period, each HI session will last approximately 50 minutes. HI will be conducted in a group of 1-3 subjects closely supervised by exercise physiologist, or some other qualified health care professional (e.g. biomedical laboratory scientist) adequately trained for the task.

Additionally, an individualized exercise training program will be prescribed for each subject in the HI group consisting of home-based low-to moderate-intensity aerobic exercises. An overall weekly goal, including supervised exercise, will be three hours of aerobic exercise e.g. walking, swimming and cycling following international guidelines (US GUIDELINE 2018). Exercise training program is described in details in table 1.

Table 1. Exercise training program in high-intensity aerobic interval group.

| Study weeks | **0–4** | **5-8** | **9-12** |
| --- | --- | --- | --- |
| *High-intensity aerobic interval training* |  |  |  |
| Duration of warm-up, min | 10 | 10 | 10 |
| High-intensity interval training frequency/week | 2 | 2 | 2 |
| Intensity of work phase, % W_max4_ | 85 | 85 | 85 |
| Intensity of recovery phase, % W_max4_ | 10 | 10 | 10 |
| Interval sets, number | 5 | 5 | 5 |
| Duration of the interval, seconds | 120-160 | 160-200 | 200-240 |
| Recovery between sets, minutes | 3 | 3 | 3 |
| Intensity of warm-up, % W_max4_ | 30 | 30 | 30 |
| Intensity of cool-down, % W_max4_ | 20 | 20 | 20 |
| Duration of cool-down, min | 5 | 5 | 5 |
| *Additional home-based aerobic training* |  |  |  |
| Duration, min/week | 140 | 120 | 100 |

W_max4_ refers to the hypothetical workload sustainable for 4 minutes.

# METHODS

## Study parameters/endpoints

### Main study parameter/endpoint

Main study parameters and endpoints include:

- NAFL disease progression, evaluated by liver biopsy and MRI/MRS scan. This is based on analysis between baseline and endpoint.
- Hepatic, muscle and adipose tissue histology and gene expression (evaluated by RNA-sequencing) that is affected by exercise intervention. This is based on liver, muscle and adipose tissue biopsy at baseline and endpoint.

### Secondary study parameters/endpoints (if applicable)

Secondary study parameters include:

- Fecal and oral microbial composition, evaluated by metagenomic sequencing. This is based on oral and fecal sample at baseline, midway and endpoint.
- Metabolite quantification of plasma and urine samples. This is based on urine samples at baseline and endpoint, and blood samples at baseline, midway and endpoint.
- Gut motility in NAFLD patients. This is based on motility MRI analysis at baseline and endpoint.

### Other study parameters (if applicable)

Other study parameters include:

- Body weight will be measured at baseline, mid-intervention and final study visits to include as possible confounding factors.
- Body composition will be measured at baseline and final study visits to include as possible confounding factors.
- Physical activity will be followed by questionnaire.
- REE will be determined at baseline and endpoint.
- Dietary intake lists will be screened before baseline, mid-intervention and final visits to see whether there is changes in dietary habits as possible confounding factor.
- Relative contribution of (RNAseq metabolic pathway based) metabolic changes in each organ (liver/muscle/adipose tissue) and gut microbiota (metabolic pathway) metabolic changes in relation to exercise induced glucose metabolism improvements.
- Calf circumference will be determined at baseline and endpoint.
- Pre- and postexercise blood biomarkers.

## Randomisation, blinding and treatment allocation

Not applicable.

## Study procedures

### Physical activity

Physical activity will be monitored by an objective physical activity monitoring through the intervention (Polar Active). Additionally, leisure time physical activity during the intervention period will be assessed by the modified Minnesota Leisure-Time Physical Activity Questionnaire (Taylor et al., 1978). The modified version to be used in the present study has not been formally validated while the original questionnaire has been validated (Bonnefoy et al., 2001). The modified version of the questionnaire is translated into Dutch from the modified Finnish questionnaire which feasibility has been, however, confirmed at the large-scale 4-year lifestyle intervention study (DR’s EXTRA) (Hakola et al., 2015). All ergospirometry tests and HI sessions will be executed in an allocated Cardio fitness program.

### Dietary intake

The subjects will get instructions to keep their diet unchanged. Online dietary intake lists will be taken as previously described (voedingscentrum.nl). Online 4-day dietary lists are taken just before the intervention period at baseline, and 5-day dietary lists are taken midway and at the end of the intervention. The online dietary intake lists will be recorded during predefined consecutive days (3-4 weekdays, 1 weekend day) and checked by researchers.

### Anthropometrics

Body weight will be measured at baseline, mid-intervention and final intervention visits. Height will be measured at baseline visit. Circumferences of waist (at the midpoint between the lateral iliac and lowest rib) and hip, thigh will be measured to the nearest 0.5 cm at baseline and endpoint visits. Body mass index (BMI) will be calculated as weight (kg) divided by height (m) squared. Body composition will be determined by bioelectrical impedance in subjects in standing position at baseline and endpoint visits together with ergospirometry test. Also, calf circumference will be measured at baseline and endpoint.

### Cardiorespiratory fitness assessment

To determine the intensity of the individually prescribed training program and the efficacy of the exercise intervention, ergospirometry tests (VO2max) will be carried out on a cycle ergometer at baseline and after 12 weeks together with the department of Cardiology (dr Jorstad) and Polifysiek, Hogeschool van Amsterdam. The test will start with a 1-minute sitting period in the saddle followed by a 3-minute warm-up with 0 Watts (W) after that the workload will increase gradually at 6 seconds intervals according to an individualized protocol. The test will be supervised by a physician or another qualified health care professional adequately trained for the task and participants will be verbally encouraged to continue until exhaustion. Respiratory gas exchange and ventilation will be measured by the breath-by-breath method and electrocardiography (ECG) will be recorded throughout the exercise test.

### Resting energy expenditure

REE will be measured at baseline and endpoint visits. Oxygen consumption and CO_2_ production will be measured for 20 minutes using a ventilated hood system. With these measurements the REE and respiratory quotient (RQ) can be calculated. The RQ represents the ratio of CO_2_ exhaled to the amount of oxygen consumed by the individual and represents whole body substrate oxidation (glucose, fat, and protein oxidation). The abbreviated Weir equation is used to calculate the 24-hour energy expenditure.

### Blood pressure and heart rate

Blood pressure and heart rate will be measured at baseline and final intervention visits.

### Magnetic Resonance Imaging

MRI scans of the abdomen area will be performed to estimate liver fat content, fibrosis, visceral and subcutaneous fat and quantitate gut motility on the 3T Philips Ingenia MRI scanner. Altogether the scans to estimate abdominal adipose tissue depot, hepatic fat and fibrosis and gut motility will take approximately 60 minutes.

### Magnetic Resonance Spectroscopy

The intrahepatic content of lipid (IHCL) will be quantified through MRS.

### Liver Transient Elastrography

Transient Elastrography, also referred to as FibroScan, will be performed to screen and identify the patients who do not have a previous diagnosis of NAFLD. FibroScan will be performed at the screening visit and also at the endpoint after the intervention period. Recommended values for different stage of fibrosis are described in Table 2.

Table 2. Recommended values for fibrosis staging**.**

|  | F0-F1(kPa) | F2 (kPa) | F3 (kPa) | F4 (kPa) |
| --- | --- | --- | --- | --- |
| NAFLD/NASH (liver stiffness) | ≤7.0 | ≥7.5 | ≤10 | ≥14.0 |

KPa, kilopascals; NAFLD, non-alcoholic fatty liver disease; NASH, non-alcoholic steatohepatitis

### Urine and microbiota samples

Stool and oral microbiota samples will be collected at baseline, midterm, and endpoint visits to determine changes in microbiota composition upon the high intensity exercise intervention. The oral samples will be taken by collecting a swab of the upper tooth rim of the front teeth in overnight fasted subjects that refrained from tooth brushing that morning. Moreover, collection on feces collection system by the subject him/herself. It is then transported to AMC on ice packs, and there it will be frozen at -80°C until analysis. The analysis will be done by sequencing at Novogene. 24-hour urine collection will be performed for at baseline and endpoint of the intervention to determine metabolite content. The samples are collected by the patient him/herself and frozen at -80°C until analysis.

### Blood samples

For determination of various biomarkers and metabolomics, total of 3 x 60 ml of venous blood samples will be withdrawn in overnight fasted state. Stated volume of venous blood includes the spare blood samples as well. Also, blood samples will be taken after the first and last exercise session for pre- and postexercise biomarkers.

### Glucose monitoring

Glucose monitoring (Freestyle Libre) will be performed at weeks at the baseline, and at weeks 11 and 12 before the end of the intervention. Altogether 4 weeks of glucose monitoring during the intervention. The subjects are instructed to perform 8-10 scans per day. After the 2-week monitoring period the results will be processed by a specific software in order to get an estimate of the daily fluctuation of blood glucose concentration.

### Liver biopsies

The ultrasound-guided liver biopsy will be performed at baseline and endpoint visits as previously described (2013_207). If a liver biopsy is already performed 12 months prior to screening, no need for baseline biopsy. After an overnight fast, the subject will be in supine position, and using an ultrasound a non-vascularised segment of the right liver lobe will be identified. A needle of 14 to 19 gauge will be used for the biopsy, and the patient will be asked to hold their breath during the biopsy. Afterwards a bandage will be put on the puncture site. The total estimated time for the biopsy is 15-30 minutes. The subject is then instructed to lie on his/her right side for 1-2 hours after the test, and pulse, blood pressure and temperature are checked after the biopsy. All samples will be stored both in formalin/paraffin and frozen in liquid nitrogen after extraction, and stored at -80°C until analysis. Ultrasound-guided percutaneous liver biopsy is a safe method at our hospital with a low complication rate (typically self-limiting bleeding) of less than 0.1% (Sporea, Popescu, & Sirli, 2008).

### Muscle biopsies

Muscle biopsies are carried out under sterile conditions and local anaesthesia (lidocaine 20 mg/ml (2 %), approximately 5 ml subcutaneously) at baseline and final intervention visits as previously described (2014_084). An incision will be made in the muscle and a biopsy will be taken. The biopsy specimen will be washed in a buffer (NaCl 0.9% / Hepes 28.3 gr/L) to remove blood. It will then be inspected for fat or fascia content and dried on gauze swabs, and subsequently stored in both formalin/paraffin and frozen in liquid nitrogen after extraction, and stored at -80°C until analysis. These biopsies will be used to estimate gene expression using RNA-sequencing.

### Subcutanous adipose tissue biopsies

At baseline and endpoint visits subcutaneous fat aspirates will be performed as previously described (2013_207). Biopsies will be carried out under sterile conditions and local anaesthesia (lidocaine 20 mg/ml (2 %), approximately 5 ml subcutaneously). Fat tissue will be obtained by hollow needle suction in the peri-umbilical area (thus no incision will be made). One half of a sample will be stored at -80°C until analysis. Haemostasis will be attained by providing local skin pressure, and suture strips will be used to close the wound and a pressure bandage will be given to minimize changes of local haematoma.

### Storage for further analysis

Samples will be stored for 15 years in biobank of the department of vascular medicine of the AMC (“Biobank vasculaire geneeskunde AMC”) in line with all applicable regulations and laws for future additional analysis. A separate protocol for the storage in the biobank will be simultaneous submitted to the “biobank toetsingscommissie”.

## Withdrawal of individual subjects

Subjects can leave the study at any time for any reason if they wish to do so without any consequences. In this case, financial compensation will be calculated based on extent of participation. The investigator can decide to withdraw a subject from the study for urgent medical reasons.

### Specific criteria for withdrawal (if applicable)

Participants with any new condition during the study that require hospital admission, change in drug prescriptions or likely to affect the outcome of this study will be withdrawn. Participants will also be withdrawn if instructions as written in the protocol are not followed.

## Replacement of individual subjects after withdrawal

Withdrawn subjects will be replaced in order to attain the desired sample size.

## Follow-up of subjects withdrawn from treatment

Subjects withdrawn for medical reasons will be followed until the interfering condition has resolved or reached a stable state.

## Premature termination of the study

Not applicable since no medicinal product is involved and since this entails a pilot study of 15 participants.

# SAFETY REPORTING

## Temporary halt for reasons of subject safety

In accordance to section 10, subsection 4, of the WMO, the sponsor will suspend the study if there is sufficient ground that continuation of the study will jeopardise subject health or safety. The sponsor will notify the accredited METC without undue delay of a temporary halt including the reason for such an action. The study will be suspended pending a further positive decision by the accredited METC. The investigator will take care that all subjects are kept informed.

## AEs and SAEs

### Adverse events (AEs)

Adverse events are defined as any undesirable experience occurring to a subject during the study, whether or not considered related to the experimental intervention. All adverse events reported spontaneously by the subject or observed by the investigator or his staff will be recorded.

### Serious adverse events (SAEs)

A serious adverse event is any untoward medical occurrence or effect that

- results in death;
- is life threatening (at the time of the event);
- requires hospitalisation or prolongation of existing inpatients’ hospitalisation;
- results in persistent or significant disability or incapacity;
- is a congenital anomaly or birth defect; or
- any other important medical event that did not result in any of the outcomes listed above due to medical or surgical intervention but could have been based upon appropriate judgement by the investigator.

An elective hospital admission will not be considered as a serious adverse event.

The investigator will report all SAEs to the sponsor without undue delay after obtaining knowledge of the events.

The sponsor will report the SAEs through the web portal *ToetsingOnline* to the accredited METC that approved the protocol, within 7 days of first knowledge for SAEs that result in death or are life threatening followed by a period of maximum of 8 days to complete the initial preliminary report. All other SAEs will be reported within a period of maximum 15 days after the sponsor has first knowledge of the serious adverse events.

## Follow-up of adverse events

All AEs will be followed until they have abated, or until a stable situation has been reached. Depending on the event, follow up may require additional tests or medical procedures as indicated, and/or referral to the general physician or a medical specialist. SAEs need to be reported till end of study within the Netherlands, as defined in the protocol.

# STATISTICAL ANALYSIS

## Primary study parameter(s)

Statistical analyses will be performed by SPSS statistical analysis software. Normality of the data will be assessed by a Kolmogorov–Smirnov test. Data transformation will be done if the data is not normally distributed. The primary analyses of studied primary parameters will be performed between the baseline and endpoint intervention by analysis of one-sample t-test, or chi-square test. If the data is not normally distributed and data transformation is not possible, the Mann-Whitney test will be used. Further associations will be assessed with Pearson rank correlation test / Pearson chi-square test.

## Secondary study parameter(s)

Metabolome, transcriptome and microbiota data will be analysed as stated above.

# ETHICAL CONSIDERATIONS

## Regulation statement

The study will be conducted according to the principles of the Declaration of Helsinki (version October 2008) and in accordance with the Medical Research Involving Human Subjects Act (WMO).

## Recruitment and consent

Subjects will be recruited by their treating physician or by advertisements in a newspaper or at the outpatient clinics. Nature, aims, methods, anticipated benefits and potential hazards related to the study will be fully explained to the subject prior to applying any Study-related procedure by principal investigator, or sub-investigators. Each subject will be informed that his medical records may be reviewed during the course of the Study or afterwards, including review by government agencies. Each subject will be informed that his medical data will be included in a database and may be reviewed during the course of the Study or afterwards, including review by government agencies. However, only authorised personnel will review these data. Each subject should be informed that the investigator will protect any personal information not related to the Study, and that individuals associated with the Study are bound by the same confidentiality obligations as other health care professionals with regard to subject confidentiality.

Each subject must give written consent according to local rules after an adequate time to consider their decision. Two copies of the consent will be signed before performing any screen evaluations. The consent form will be written in a language that the subject can read and understand. The patient information letter and informed consent form should be attached as a separate document.

The investigator will explain that the subjects are completely free to refuse to enter the Study or to withdraw from it at any time, without any consequences for their further care and without need for justification. The investigator will complete and sign the informed consent section of the CRF for each subject enrolled.

## Benefits and risks assessment, group relatedness

Participant may benefit in the short term from the exercise since it may improve their physical fitness and motivation to actively perform exercise. Additionally, patients are helping to further unravel the relation between exercise induced changes in gut microbiota in relation to metabolic control in liver, muscle and adipose tissue which may lead to novel diagnostic and therapeutic leads

The placing of the intravenous cannula in our study can be an unpleasant experience for the subjects and may result in (self limiting) bruising.

An experienced interventional radiologist will perform ultrasound-guided liver biopsy. Ultrasound-guided percutaneous liver biopsy is a safe method with very low the risk of complications (< 0.1 %) comprising mostly bleeding from the biopsy site. Moreover, local hemostasis after the procedure can be observed. Liver biopsy will be performed under local anaesthesia.

Adipose tissue biopsy will be taken from the fat of the abdomen. The abdominal skin will be anesthetized at the site of the biopsy so it will be painless for the participants. However, it is unpleasant but not harmful since anaesthetic is a small prick and may feel uncomfortable. In few hours after the biopsy, the area of biopsy may feel a bit sore and a bruise may develop.

Muscle biopsy will be taken from the muscle of leg under aesthetic conditions. Following a muscle biopsy the leg may stay sensitive for a few days. It is a minor intervention, usually without any events but like any procedure there is a minor risk of bleeding or infection.

## Compensation for injury

The sponsor/investigator has a liability insurance which is in accordance with article 7 of the WMO. The sponsor (also) has an insurance which is in accordance with the legal requirements in the Netherlands (Article 7 WMO). This insurance provides cover for damage to research subjects through injury or death caused by the study. The insurance applies to the damage that becomes apparent during the study or within 4 years after the end of the study.

1. € 650.000,-- (i.e. six hundred and fifty thousand Euro) for death or injury for each subject who participate in the Research;
2. € 5.000.000,-- (i.e. five million Euro) for death or injury for all subjects who participate in the Research;
3. € 7.500.000,-- (i.e. seven million five hundred thousand Euro) for the total damage incurred by the organisation for all damage disclosed by scientific research for the Sponsor as ‘verrichter’ in the meaning of said Act in each year of insurance coverage.

## Incentives (if applicable)

All study participants will receive a financial compensation €350 and a refund for travel costs. Financial compensation is based on the burden and the time spent in the study (21 hours per participant, excluding training). The compensation thus equals 17 euros per hour.

# ADMINISTRATIVE ASPECTS, MONITORING AND PUBLICATION

## Handling and storage of data and documents

Multiple endpoint measures are not included in subject’s medical records and are entered directly into the eCRF. This applies to the subject’s lifestyle and intoxication information, the subject’s current use of medication and lifestyle, administrative information related to the MRI/MRS scan, the ultrasound guided liver and muscle biopsy, and adipose tissue biopsy, and on the collection of blood, oral, fecal and 24 h urine samples and the exercise test (ergospirometry), as well as the dietary intake data, BIA, REE and glucose monitor results*.* All data will be registered under a unique study code. The key to this code will only be known to the principal‐ and coordinating investigators. Furthermore, all research and medical data will be kept confidential. Only the researchers that are involved in this study in AMC will be able to see the data and identify participants. After the study has been finished, all the data will be kept at the AMC. Research material will be stored 15 years. By signing the informed consent, participants allow future analysis of stored material. Members of the department of National Health or representatives of the research council or local medical ethics committee have access to the data at any time. This information will be included in the written informed consent.

## Monitoring and Quality Assurance

## Amendments

Amendments are changes made to the research after a favourable opinion by the accredited METC has been given. All amendments will be notified to the METC that gave a favourable opinion.

## Annual progress report

The sponsor/investigator will submit a summary of the progress of the trial to the accredited METC once a year. Information will be provided on the date of inclusion of the first subject, numbers of subjects included and numbers of subjects that have completed the trial, serious adverse events/ serious adverse reactions, other problems, and amendments.

## Temporary halt and (prematurely) end of study report

The investigator/sponsor will notify the accredited METC of the end of the study within a period of 8 weeks. The end of the study is defined as the last patient’s last visit.

The sponsor will notify the METC immediately of a temporary halt of the study, including the reason of such an action.

In case the study is ended prematurely, the sponsor will notify the accredited METC within 15 days, including the reasons for the premature termination.

Within one year after the end of the study, the investigator/sponsor will submit a final study report with the results of the study, including any publications/abstracts of the study, to the accredited METC.

## Public disclosure and publication policy

The trial will be registered in the Netherland Trial before study onset. The results of this study will be submitted for publication in an international, peer-reviewed journal.

# REFERENCES

Allen, J. M., Mailing, L. J., Cohrs, J., Salmonson, C., Fryer, J. D., Nehra, V., … Woods, J. A. (2018). Exercise training-induced modification of the gut microbiota persists after microbiota colonization and attenuates the response to chemically-induced colitis in gnotobiotic mice. *Gut Microbes*, *9*(2), 115–130. https://doi.org/10.1080/19490976.2017.1372077

Allen, Jacob M., Mailing, L. J., Niemiro, G. M., Moore, R., Cook, M. D., White, B. A., … Woods, J. A. (2018). Exercise Alters Gut Microbiota Composition and Function in Lean and Obese Humans. *Medicine and Science in Sports and Exercise*, *50*(4), 747–757. https://doi.org/10.1249/MSS.0000000000001495

Almanza, D., Gharaee-Kermani, M., Zhilin-Roth, A., Rodriguez-Nieves, J. A., Colaneri, C., Riley, T., & Macoska, J. A. (2018). Nonalcoholic Fatty Liver Disease Demonstrates a Pre-fibrotic and Premalignant Molecular Signature. *Digestive Diseases and Sciences*. https://doi.org/10.1007/s10620-018-5398-4

Alonso, C., Fernández-Ramos, D., Varela-Rey, M., Martínez-Arranz, I., Navasa, N., Van Liempd, S. M., … Mato, J. M. (2017). Metabolomic Identification of Subtypes of Nonalcoholic Steatohepatitis. *Gastroenterology*, *152*(6), 1449-1461.e7. https://doi.org/10.1053/j.gastro.2017.01.015

Anderson, E. L., Fraser, A., Howe, L. D., Callaway, M. P., Sattar, N., Day, C., … Lawlor, D. A. (2016). Physical Activity Is Prospectively Associated With Adolescent Nonalcoholic Fatty Liver Disease. *Journal of Pediatric Gastroenterology and Nutrition*, *62*(1), 110–117. https://doi.org/10.1097/MPG.0000000000000904

Aragonès, G., Auguet, T., Armengol, S., Berlanga, A., Guiu-Jurado, E., Aguilar, C., … Richart, C. (2016). PNPLA3 Expression Is Related to Liver Steatosis in Morbidly Obese Women with Non-Alcoholic Fatty Liver Disease. *International Journal of Molecular Sciences*, *17*(5). https://doi.org/10.3390/ijms17050630

Bedossa, P. (2017). Pathology of non-alcoholic fatty liver disease. *Liver International*, *37 Suppl 1*, 85–89. https://doi.org/10.1111/liv.13301

Bonnefoy, M., Normand, S., Pachiaudi, C., Lacour, J. R., Laville, M., & Kostka, T. (2001). Simultaneous validation of ten physical activity questionnaires in older men: a doubly labeled water study. *Journal of the American Geriatrics Society*, *49*(1), 28–35. https://doi.org/10.1046/j.1532-5415.2001.49006.x

Chedid, M. F. (2017). Nonalcoholic Steatohepatitis: The Second Leading Indication for Liver Transplantation in the USA. *Digestive Diseases and Sciences*, *62*(10), 2621–2622. https://doi.org/10.1007/s10620-017-4724-6

Damas, F., Ugrinowitsch, C., Libardi, C. A., Jannig, P. R., Hector, A. J., McGlory, C., … Phillips, S. M. (2018). Resistance training in young men induces muscle transcriptome-wide changes associated with muscle structure and metabolism refining the response to exercise-induced stress. *European Journal of Applied Physiology*, *118*(12), 2607–2616. https://doi.org/10.1007/s00421-018-3984-y

de Alwis, N. M. W., & Day, C. P. (2008). Non-alcoholic fatty liver disease: the mist gradually clears. *Journal of Hepatology*, *48 Suppl 1*, S104-12. https://doi.org/10.1016/j.jhep.2008.01.009

Deschasaux M, Bouter KE, Prodan A, et al. Depicting the composition of gut microbiota in a population with varied ethnic origins but shared geography. *Nat Med*. 2018;24(10):1526-1531. doi:10.1038/s41591-018-0160-1

de Jonge, C. S., Smout, A. J. P. M., Nederveen, A. J., & Stoker, J. (2018). Evaluation of gastrointestinal motility with MRI: Advances, challenges and opportunities. *Neurogastroenterology & Motility*, *30*(1), e13257. https://doi.org/10.1111/nmo.13257

de Jonge, Catharina S, Gollifer, R. M., Nederveen, A. J., Atkinson, D., Taylor, S. A., Stoker, J., & Menys, A. (2018). Dynamic MRI for bowel motility imaging–how fast and how long? *The British Journal of Radiology*, 20170845. https://doi.org/10.1259/bjr.20170845

de Wit, N. J. W., Afman, L. A., Mensink, M., & Müller, M. (2012). Phenotyping the effect of diet on non-alcoholic fatty liver disease. *Journal of Hepatology*, *57*(6), 1370–1373. https://doi.org/10.1016/j.jhep.2012.07.003

Ekstedt, M., Franzén, L. E., Mathiesen, U. L., Thorelius, L., Holmqvist, M., Bodemar, G., & Kechagias, S. (2006). Long-term follow-up of patients with NAFLD and elevated liver enzymes. *Hepatology (Baltimore, Md.)*, *44*(4), 865–873. https://doi.org/10.1002/hep.21327

European Association for the Study of the Liver (EASL), European Association for the Study of Diabetes (EASD), & European Association for the Study of Obesity (EASO). (2016). EASL-EASD-EASO Clinical Practice Guidelines for the management of non-alcoholic fatty liver disease. *Journal of Hepatology*, *64*(6), 1388–1402. https://doi.org/10.1016/j.jhep.2015.11.004

Farzanegi, P., Dana, A., Ebrahimpoor, Z., Asadi, M., & Azarbayjani, M. A. (2019). Mechanisms of beneficial effects of exercise training on non-alcoholic fatty liver disease (NAFLD): Roles of oxidative stress and inflammation. *European Journal of Sport Science*, 1–10. https://doi.org/10.1080/17461391.2019.1571114

Fischer, L. M., Reid, R. W., Spencer, M. D., Fodor, A. A., Zeisel, S. H., & Hamp, T. J. (2010). Association Between Composition of the Human Gastrointestinal Microbiome and Development of Fatty Liver With Choline Deficiency. *Gastroenterology*, *140*(3), 976–986. https://doi.org/10.1053/j.gastro.2010.11.049

Friedman, S. L., Neuschwander-Tetri, B. A., Rinella, M., & Sanyal, A. J. (2018). Mechanisms of NAFLD development and therapeutic strategies. *Nature Medicine*, *24*(7), 908–922. https://doi.org/10.1038/s41591-018-0104-9

Gerhard, G. S., Legendre, C., Still, C. D., Chu, X., Petrick, A., & DiStefano, J. K. (2018). Transcriptomic Profiling of Obesity-Related Nonalcoholic Steatohepatitis Reveals a Core Set of Fibrosis-Specific Genes. *Journal of the Endocrine Society*, *2*(7), 710–726. https://doi.org/10.1210/js.2018-00122

Gitto, S., Schepis, F., Andreone, P., & Villa, E. (2018). Study of the Serum Metabolomic Profile in Nonalcoholic Fatty Liver Disease: Research and Clinical Perspectives. *Metabolites*, *8*(1), 17. https://doi.org/10.3390/metabo8010017

Hadizadeh, F., Walter, S., Belheouane, M., Bonfiglio, F., Heinsen, F.-A., Andreasson, A., … D’Amato, M. (2017). Stool frequency is associated with gut microbiota composition. *Gut*, *66*(3), 559–560. https://doi.org/10.1136/gutjnl-2016-311935

Hajighasem, A., Farzanegi, P., & Mazaheri, Z. (2018). Effects of combined therapy with resveratrol, continuous and interval exercises on apoptosis, oxidative stress, and inflammatory biomarkers in the liver of old rats with non-alcoholic fatty liver disease. *Archives of Physiology and Biochemistry*. https://doi.org/10.1080/13813455.2018.1441872

Hakola, L., Savonen, K., Komulainen, P., Hassinen, M., Rauramaa, R., & Lakka, T. A. (2015). Moderators of Maintained Increase in Aerobic Exercise Among Aging Men and Women in a 4-Year Randomized Controlled Trial: The DR’s EXTRA Study. *Journal of Physical Activity & Health*, *12*(11), 1477–1484. https://doi.org/10.1123/jpah.2014-0299

Hallsworth, K., Fattakhova, G., Hollingsworth, K. G., Thoma, C., Moore, S., Taylor, R., … Trenell, M. I. (2011). Resistance exercise reduces liver fat and its mediators in non-alcoholic fatty liver disease independent of weight loss. *Gut*, *60*(9), 1278–1283. https://doi.org/10.1136/gut.2011.242073

Hallsworth, K., Thoma, C., Hollingsworth, K. G., Cassidy, S., Anstee, Q. M., Day, C. P., & Trenell, M. I. (2015). Modified high-intensity interval training reduces liver fat and improves cardiac function in non-alcoholic fatty liver disease: A randomized controlled trial. *Clinical Science*, *129*(12), 1097–1105. https://doi.org/10.1042/CS20150308

Henao-Mejia, J., Elinav, E., Jin, C., Hao, L., Mehal, W. Z., Strowig, T., … Flavell, R. A. (2012). Inflammasome-mediated dysbiosis regulates progression of NAFLD and obesity. *Nature*, *482*(7384), 179–185. https://doi.org/10.1038/nature10809

Hinkley, J. M., Konopka, A. R., Suer, M. K., & Harber, M. P. (2017). Short-term intense exercise training reduces stress markers and alters the transcriptional response to exercise in skeletal muscle. *American Journal of Physiology. Regulatory, Integrative and Comparative Physiology*, *312*(3), R426–R433. https://doi.org/10.1152/ajpregu.00356.2016

Horner, K. M., Schubert, M. M., Desbrow, B., Byrne, N. M., & King, N. A. (2015). Acute Exercise and Gastric Emptying: A Meta-Analysis and Implications for Appetite Control. *Sports Medicine*, *45*(5), 659–678. https://doi.org/10.1007/s40279-014-0285-4

Houghton, D., Thoma, C., Hallsworth, K., Cassidy, S., Hardy, T., Burt, A. D., … Trenell, M. I. (2017). Exercise Reduces Liver Lipids and Visceral Adiposity in Patients With Nonalcoholic Steatohepatitis in a Randomized Controlled Trial. *Clinical Gastroenterology and Hepatology : The Official Clinical Practice Journal of the American Gastroenterological Association*, *15*(1), 96-102.e3. https://doi.org/10.1016/j.cgh.2016.07.031

Johnson, N. A., Sachinwalla, T., Walton, D. W., Smith, K., Armstrong, A., Thompson, M. W., & George, J. (2009). Aerobic exercise training reduces hepatic and visceral lipids in obese individuals without weight loss. *Hepatology (Baltimore, Md.)*, *50*(4), 1105–1112. https://doi.org/10.1002/hep.23129

Kantartzis, K., Thamer, C., Peter, A., Machann, J., Schick, F., Schraml, C., … Stefan, N. (2009). High cardiorespiratory fitness is an independent predictor of the reduction in liver fat during a lifestyle intervention in non-alcoholic fatty liver disease. *Gut*, *58*(9), 1281–1288. https://doi.org/10.1136/gut.2008.151977

Katsagoni, C. N., Georgoulis, M., Papatheodoridis, G. V., Panagiotakos, D. B., & Kontogianni, M. D. (2017). Effects of lifestyle interventions on clinical characteristics of patients with non-alcoholic fatty liver disease: A meta-analysis. *Metabolism: Clinical and Experimental*, *68*, 119–132. https://doi.org/10.1016/j.metabol.2016.12.006

Keating, S. E., Hackett, D. A., Parker, H. M., O’Connor, H. T., Gerofi, J. A., Sainsbury, A., … Johnson, N. A. (2015). Effect of aerobic exercise training dose on liver fat and visceral adiposity. *Journal of Hepatology*, *63*(1), 174–182. https://doi.org/10.1016/j.jhep.2015.02.022

Keller, P., Vollaard, N. B. J., Gustafsson, T., Gallagher, I. J., Sundberg, C. J., Rankinen, T., … Timmons, J. A. (2011). A transcriptional map of the impact of endurance exercise training on skeletal muscle phenotype. *Journal of Applied Physiology (Bethesda, Md. : 1985)*, *110*(1), 46–59. https://doi.org/10.1152/japplphysiol.00634.2010

Knaapen, M., Kootte, R. S., Zoetendal, E. G., de Vos, W. M., Dallinga-Thie, G. M., Levi, M., … Nieuwdorp, M. (2013). Obesity, non-alcoholic fatty liver disease, and atherothrombosis: a role for the intestinal microbiota? *Clinical Microbiology and Infection : The Official Publication of the European Society of Clinical Microbiology and Infectious Diseases*, *19*(4), 331–337. https://doi.org/10.1111/1469-0691.12170

Koehler, E. M., Plompen, E. P. C., Schouten, J. N. L., Hansen, B. E., Darwish Murad, S., Taimr, P., … Janssen, H. L. A. (2016). Presence of diabetes mellitus and steatosis is associated with liver stiffness in a general population: The Rotterdam study. *Hepatology (Baltimore, Md.)*, *63*(1), 138–147. https://doi.org/10.1002/hep.27981

Lou, Y., Tian, G.-Y., Song, Y., Liu, Y.-L., Chen, Y.-D., Shi, J.-P., & Yang, J. (2017). Characterization of transcriptional modules related to fibrosing-NAFLD progression. *Scientific Reports*, *7*(1), 4748. https://doi.org/10.1038/s41598-017-05044-2

Lundberg, T. R., Fernandez-Gonzalo, R., Tesch, P. A., Rullman, E., & Gustafsson, T. (2016). Aerobic exercise augments muscle transcriptome profile of resistance exercise. *American Journal of Physiology. Regulatory, Integrative and Comparative Physiology*, *310*(11), R1279-87. https://doi.org/10.1152/ajpregu.00035.2016

Miele, L., Valenza, V., La Torre, G., Montalto, M., Cammarota, G., Ricci, R., … Grieco, A. (2009). Increased intestinal permeability and tight junction alterations in nonalcoholic fatty liver disease. *Hepatology (Baltimore, Md.)*, *49*(6), 1877–1887. https://doi.org/10.1002/hep.22848

Mouzaki, M., Comelli, E. M., Arendt, B. M., Bonengel, J., Fung, S. K., Fischer, S. E., … Allard, J. P. (2013). Intestinal microbiota in patients with nonalcoholic fatty liver disease. *Hepatology (Baltimore, Md.)*, *58*(1), 120–127. https://doi.org/10.1002/hep.26319

Moylan, C. A., Pang, H., Dellinger, A., Suzuki, A., Garrett, M. E., Guy, C. D., … Diehl, A. M. (2014). Hepatic gene expression profiles differentiate presymptomatic patients with mild versus severe nonalcoholic fatty liver disease. *Hepatology (Baltimore, Md.)*, *59*(2), 471–482. https://doi.org/10.1002/hep.26661

Munukka, E., Ahtiainen, J. P., Puigbó, P., Jalkanen, S., Pahkala, K., Keskitalo, A., … Pekkala, S. (2018). Six-week endurance exercise alters gut metagenome that is not reflected in systemic metabolism in over-weight women. *Frontiers in Microbiology*, *9*(OCT). https://doi.org/10.3389/fmicb.2018.02323

Oseini, A. M., & Sanyal, A. J. (2017). Therapies in non-alcoholic steatohepatitis (NASH). *Liver International : Official Journal of the International Association for the Study of the Liver*, *37 Suppl 1*, 97–103. https://doi.org/10.1111/liv.13302

Patterson, R., McNamara, E., Tainio, M., de Sá, T. H., Smith, A. D., Sharp, S. J., … Wijndaele, K. (2018). Sedentary behaviour and risk of all-cause, cardiovascular and cancer mortality, and incident type 2 diabetes: a systematic review and dose response meta-analysis. *European Journal of Epidemiology*, *33*(9), 811–829. https://doi.org/10.1007/s10654-018-0380-1

Porras, D., Nistal, E., Martínez-Flórez, S., González-Gallego, J., García-Mediavilla, M. V., & Sánchez-Campos, S. (2018). Intestinal Microbiota Modulation in Obesity-Related Non-alcoholic Fatty Liver Disease. *Frontiers in Physiology*, *9*(December), 1813. https://doi.org/10.3389/fphys.2018.01813

Promrat, K., Kleiner, D. E., Niemeier, H. M., Jackvony, E., Kearns, M., Wands, J. R., … Wing, R. R. (2010). Randomized controlled trial testing the effects of weight loss on nonalcoholic steatohepatitis. *Hepatology*. https://doi.org/10.1002/hep.23276

Raman, M., Ahmed, I., Gillevet, P. M., Probert, C. S., Ratcliffe, N. M., Smith, S., … Rioux, K. P. (2013). Fecal microbiome and volatile organic compound metabolome in obese humans with nonalcoholic fatty liver disease. *Clinical Gastroenterology and Hepatology : The Official Clinical Practice Journal of the American Gastroenterological Association*, *11*(7), 868-75.e1-3. https://doi.org/10.1016/j.cgh.2013.02.015

Ratziu, V. (2017). Non-pharmacological interventions in non-alcoholic fatty liver disease patients. *Liver International : Official Journal of the International Association for the Study of the Liver*, *37 Suppl 1*, 90–96. https://doi.org/10.1111/liv.13311

Rich NE, Oji S, Mufti AR, et al. Racial and Ethnic Disparities in Nonalcoholic Fatty Liver Disease Prevalence, Severity, and Outcomes in the United States: A Systematic Review and Meta-analysis. *Clin Gastroenterol Hepatol*. 2018;16(2):198-210.e2. doi:10.1016/j.cgh.2017.09.041

Roager, H. M., Hansen, L. B. S., Bahl, M. I., Frandsen, H. L., Carvalho, V., Gøbel, R. J., … Licht, T. R. (2016). Colonic transit time is related to bacterial metabolism and mucosal turnover in the gut. *Nature Microbiology*, *1*(9), 16093. https://doi.org/10.1038/nmicrobiol.2016.93

Sales, R. C., Medeiros, P. C., Spreafico, F., de Velasco, P. C., Gonçalves, F. K. A., Martín-Hernández, R., … Tavares do Carmo, M. G. (2018). Olive Oil, Palm Oil, and Hybrid Palm Oil Distinctly Modulate Liver Transcriptome and Induce NAFLD in Mice Fed a High-Fat Diet. *International Journal of Molecular Sciences*, *20*(1). https://doi.org/10.3390/ijms20010008

Shamsoddini, A., Sobhani, V., Ghamar Chehreh, M. E., Alavian, M., & Zaree, A. (2016). The Effects of Aerobic and Resistance Exercise Training on Liver Enzymes and Hepatic Fat in Iranian Men With Nonalcoholic Fatty Liver Disease. *Hepatitis Monthly*. https://doi.org/10.5812/hepatmon.35162

Sheldon, R. D., Kanosky, K. M., Wells, K. D., Miles, L., Perfield, J. W., Xanthakos, S., … Rector, R. S. (2016). Transcriptomic differences in intra-abdominal adipose tissue in extremely obese adolescents with different stages of NAFLD. *Physiological Genomics*, *48*(12), 897–911. https://doi.org/10.1152/physiolgenomics.00020.2016

Sporea, I., Popescu, A., & Sirli, R. (2008). Why, who and how should perform liver biopsy in chronic liver diseases. *World Journal of Gastroenterology*, *14*(21), 3396–3402.

Starmann, J., Fälth, M., Spindelböck, W., Lanz, K.-L., Lackner, C., Zatloukal, K., … Sültmann, H. (2012). Gene expression profiling unravels cancer-related hepatic molecular signatures in steatohepatitis but not in steatosis. *PloS One*, *7*(10), e46584. https://doi.org/10.1371/journal.pone.0046584

Sullivan, S., Kirk, E. P., Mittendorfer, B., Patterson, B. W., & Klein, S. (2012). Randomized trial of exercise effect on intrahepatic triglyceride content and lipid kinetics in nonalcoholic fatty liver disease. *Hepatology (Baltimore, Md.)*, *55*(6), 1738–1745. https://doi.org/10.1002/hep.25548

Suppli, M. P., Rigbolt, K. T. G., Veidal, S. S., Heebøl, S., Lykke Eriksen, P., Demant, M., … Knop, F. K. (2019). HEPATIC TRANSCRIPTOME SIGNATURES IN PATIENTS WITH VARYING DEGREES OF NON-ALCOHOLIC FATTY LIVER DISEASE COMPARED TO HEALTHY NORMAL-WEIGHT INDIVIDUALS. *American Journal of Physiology. Gastrointestinal and Liver Physiology*. https://doi.org/10.1152/ajpgi.00358.2018

Taniguchi, H., Tanisawa, K., Sun, X., Kubo, T., Hoshino, Y., Hosokawa, M., … Higuchi, M. (2018). Effects of short-term endurance exercise on gut microbiota in elderly men. *Physiological Reports*, *6*(23), e13935. https://doi.org/10.14814/phy2.13935

Taylor, H. L., Jacobs, D. R., Schucker, B., Knudsen, J., Leon, A. S., & Debacker, G. (1978). A questionnaire for the assessment of leisure time physical activities. *Journal of Chronic Diseases*, *31*(12), 741–755. https://doi.org/10.1016/0021-9681(78)90058-9

Tornvall G. (1963). Assessment of physical capabilities. *Acta Physiol Scand Suppl* , *58*(201), 1–102.

Tottey, W., Feria-Gervasio, D., Gaci, N., Laillet, B., Pujos, E., Martin, J.-F., … Brugère, J.-F. (2017). Colonic Transit Time Is a Driven Force of the Gut Microbiota Composition and Metabolism: In Vitro Evidence. *Journal of Neurogastroenterology and Motility*, *23*(1), 124–134. https://doi.org/10.5056/jnm16042

Trefts, E., Williams, A. S., & Wasserman, D. H. (2015). Exercise and the Regulation of Hepatic Metabolism. *Progress in Molecular Biology and Translational Science*, *135*, 203–225. https://doi.org/10.1016/bs.pmbts.2015.07.010

US Department of Health and Human Services. (2018). *Physical Activity Guidelines for Americans 2 nd edition*. Washington, DC.

Vilar-Gomez, E., Martinez-Perez, Y., Calzadilla-Bertot, L., Torres-Gonzalez, A., Gra-Oramas, B., Gonzalez-Fabian, L., … Romero-Gomez, M. (2015). Weight Loss Through Lifestyle Modification Significantly Reduces Features of Nonalcoholic Steatohepatitis. *Gastroenterology*. https://doi.org/10.1053/j.gastro.2015.04.005

Wigg, A. J., Roberts-Thomson, I. C., Dymock, R. B., McCarthy, P. J., Grose, R. H., & Cummins, A. G. (2001). The role of small intestinal bacterial overgrowth, intestinal permeability, endotoxaemia, and tumour necrosis factor alpha in the pathogenesis of non-alcoholic steatohepatitis. *Gut*, *48*(2), 206–211. https://doi.org/10.1136/gut.48.2.206

Ye, H., & Liu, W. (2015). Transcriptional networks implicated in human nonalcoholic fatty liver disease. *Molecular Genetics and Genomics : MGG*, *290*(5), 1793–1804. https://doi.org/10.1007/s00438-015-1037-3

Younossi, Z. M. (2019). Non-alcoholic fatty liver disease – A global public health perspective. *Journal of Hepatology*, *70*(3), 531–544. https://doi.org/10.1016/j.jhep.2018.10.033

Younossi, Z. M., Koenig, A. B., Abdelatif, D., Fazel, Y., Henry, L., & Wymer, M. (2016). Global epidemiology of nonalcoholic fatty liver disease-Meta-analytic assessment of prevalence, incidence, and outcomes. *Hepatology (Baltimore, Md.)*, *64*(1), 73–84. https://doi.org/10.1002/hep.28431

Zelber-Sagi, S., Buch, A., Yeshua, H., Vaisman, N., Webb, M., Harari, G., … Shibolet, O. (2014). Effect of resistance training on non-alcoholic fatty-liver disease a randomized-clinical trial. *World Journal of Gastroenterology*, *20*(15), 4382–4392. https://doi.org/10.3748/wjg.v20.i15.4382

Zhu, R., Baker, S. S., Moylan, C. A., Abdelmalek, M. F., Guy, C. D., Zamboni, F., … Zhu, L. (2016). Systematic transcriptome analysis reveals elevated expression of alcohol-metabolizing genes in NAFLD livers. *The Journal of Pathology*, *238*(4), 531–542. https://doi.org/10.1002/path.4650
