# Supplementary material for: Deep phenotyping of patients with MASLD upon high-intensity interval training
Source: JHEP Rep. 2024 Dec 16;7(3):101289. doi: 10.1016/j.jhepr.2024.101289 (PMC11883402; doi:10.1016/j.jhepr.2024.101289)
Supplement: Multimedia component 5 [file mmc5.zip › Clinical Trial/E1-2 PIF-ICF CRISTINA versie 9.0 dd 8-7-2020 final ENG.docx]

**Patient information for participation in medical scientific research**

**The CRISTINA study**

The influence of an intensive training program on fatty liver and intestinal bacteria

Study title in English: An Exercise Intervention Study in NAFLD Patients

**Introduction**

Dear sir/madam,

We kindly ask you to participate in a study in which we investigate the effect of exercise on non-alcoholic fatty liver disease (NAFLD) and whether these expected effects are mediated by the intestinal bacteria (gut microbiota). Before you decide whether you want to participate in this study, it is important that you know why this research is being done and what your participation entails. Please read the following information carefully and ask questions to your doctor or the study coordinators. By signing the attached consent form, you indicate that you wish to participate in the study.

**1. General information**

This research is performed at the Department of Vascular Medicine, Amsterdam University Medical Centers, location AMC. This study has been approved by the medical ethics review committee. General information on the quality requirements of research can be found in the brochure 'Medical scientific research' (Appendix H).

**2. What is the background and what is the purpose of this study?**

NAFLD is the most common liver disease worldwide. It comprises a number of stages, starting with isolated fatty liver (hepatic steatosis, or non-alcoholic fatty liver (NAFL), followed by the more progressive stages non-alcoholic steatohepatitis (NASH), and NASH-related fibrosis, in some cases resulting in liver cirrhosis and hepatocellular carcinoma (HCC). Metabolic disorders such as obesity and the metabolic syndrome (characterized by dysregulation of lipid and glycemic control) are strongly associated with the development of NAFLD. However, the mechanisms leading to the progression of NAFLD are incompletely understood. There is evidence that gut bacteria play a role in the development and progression of NAFLD.

Currently, there are no pharmacological therapeutics to treat NAFLD. Internationally recommended therapies include lifestyle modifications, including dietary changes and an increase in physical activity. Exercise is known to have beneficial effects on overall human health, as well as directly on the liver itself. However, the question is how this relates to other involved organs such as muscle and fat tissue. Moreover, research shows that exercise affects gut bacteria that are thought to play a role in the development of NAFLD.

Because NAFLD is increasing in prevalence and there is of yet no medication to treat it, we aim to unravel the underlying disease mechanisms, in particular the role of the gut microbiota. To do so, we intend to include 30 participants between 18-70 years of age with NAFLD in this study. The study participants will follow a high-intensity interval training for 12 weeks.

**3. How is the research conducted?**

If you participate, the study will have a duration of approximately 3 months in total.

**Screening visit**

If you decide to participate in the study and you have signed the informed consent form, we will first determine whether you can participate in the study. The examiner will, among other things, take your medical history, including current medication use, ask questions and perform a physical examination, draw your blood and perform a Fibroscan examination.

**Therapy**

If you participate in the study, you will exercise twice a week for 12 weeks under the supervision (cardio fitness) of a physiotherapist. During these sessions you will receive instructions on how to do the training. You will also do exercises at home, so that you are exercising for a total of at least 3 hours per week.

**Visits and measurements**

If you are eligible for the study, depending on the results of the screening visit, we will invite you to the subsequent visits (6 visits of 21 hours in total). See Table 1 and Figure 1 for a detailed overview.

**Tabel 1.** Content and duration of the study visits.

| **Period** | **Study appoinment** | **Content** |
| --- | --- | --- |
| **Before training period** | **Initial interview** | Explanation of the research + assessment if you can participate in the research. |
|  | **Visit 1**  Screening  Week -2  Total time: 1 h | Explanation of the research + signing the consent form.  Screening whether you can participate in the study:   Fasting blood sample.   Physical examination.   Fibroscan of the liver. |
|  | **Visit 2**  Week -1  Total time: 6 h |  Fasting blood sample.   Measurement of energy consumption at rest   Adipose tissue biopsies.   Saliva sample.   MRI examination.   Fitting glucose monitor (FreeStyle Libre) to be worn for the period of 14 days. |
|  | **Visit 3**  Week 0  Total time: 3 h |  Liver and muscle biopsy (ultrasound guided).   Collection of 24h urine and morning stools.   Checking online diet list of previous 4 days. |
| **Training period** | Start training  (training 2 x week)  Week 1 | Exercise test (ergospirometry) and body composition.  Start training session.  Blood sample taken after first training session.  Start of the physical activity questionnaire. |
|  | **Visit 4**  Week 6  Total time: 1 h |  Measurements of body weight.   Collection of stool and saliva sample.   Fasting blood sample.   Checking online diet list of previous 5 days. |
|  | End training  Week 12 | Last training session and blood draw after the last session.  Exercise test (ergospirometry) and body composition.  Closing the physical activity questionnaire. |
| **After training period** | **Visit 5**  Week 12  Total time: 6 h |  Fasting blood sample.   Measurement of energy consumption at rest.   Adipose tissue biopsies.   Saliva sample.   MRI examination and Fibroscan of the liver.   Fitting glucose monitors (FreeStyle Libre) worn for 14 days. |
|  | **Bezoek 6**  Week 12  Total time: 3 h |  Liver and muscle biopsy (ultrasound guided).   Collection of 24h urine and morning faeces.   Checking online diet list of previous 5 days. |


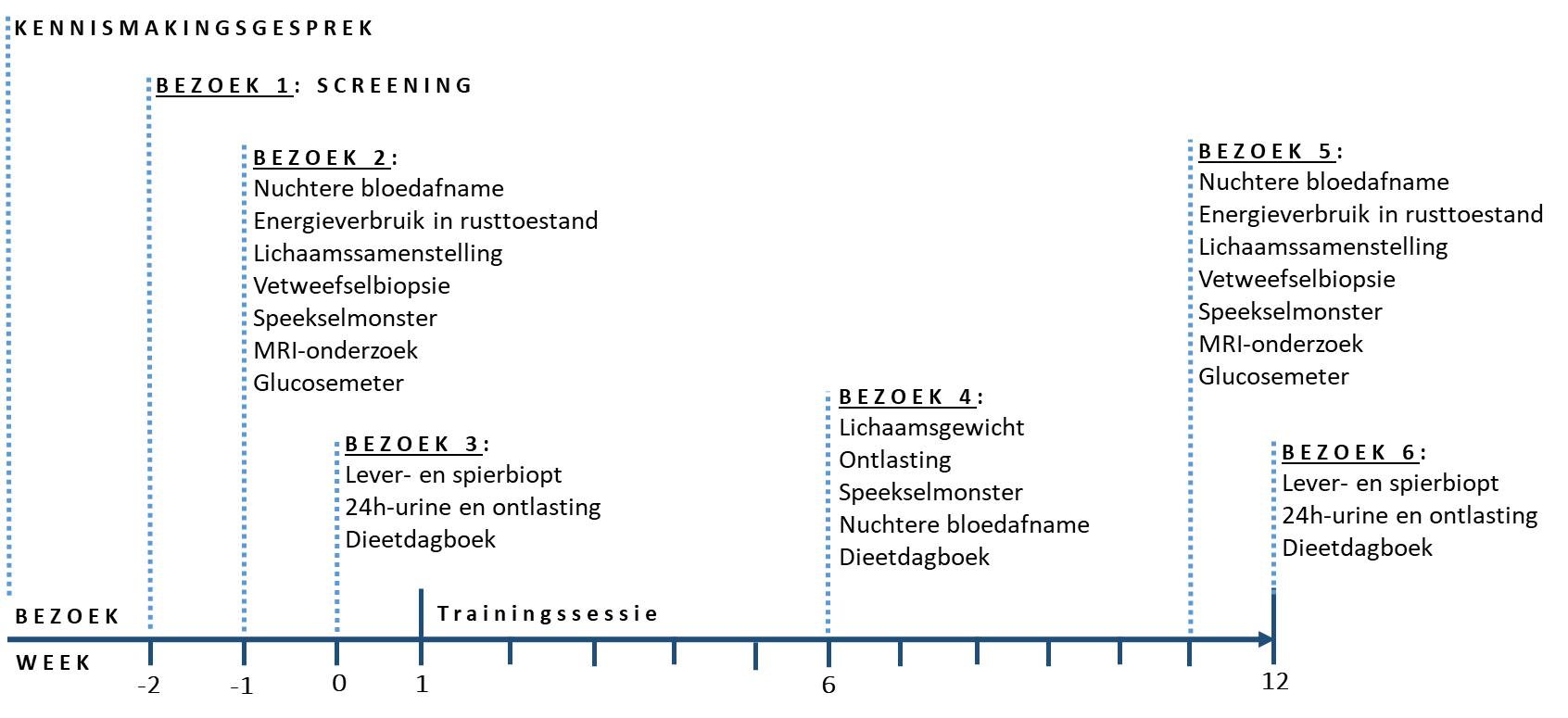


**Figure 1.** The timeline of the study visits.

**4. What is expected of you?**

To ensure that the investigation runs smoothly and for your own safety, we ask that you comply with the following instructions:

• Try to follow the examiner's instructions as closely as possible during all measurements

• Do not participate in any other medical-scientific research study.

• Try to come to all appointments for your study visits.

We ask you to contact the study doctor in the following cases:

• Before taking any other medication. Also when these are homeopathic drugs, natural medicines, vitamins and / or any medication from a pharmacy.

• If you are admitted or have been treated in a hospital.

• If you suddenly have health problems.

• If you no longer wish to participate in the study.

• If your contact details change.

**5. What do the examinations entail and what discomfort may you expect from participating in the study?**

Sports intervention and exercise test (ergo spirometry): Participation in the study means that you have to exercise regularly for three months. This can be experienced as difficult and it also takes time. By means of the exercise test at the beginning of the study, we will adapt the further training program to your individual level. For instructions on training sessions and the exercise test, see appendix E. In summary, the sports program includes the following:

- High intensity training on a spin bike, twice a week in a build-up schedule from 40 to 50 minutes. Additional moderate-intensity home exercise consisting of walking, swimming or cycling.

Diet and food diary: You will follow your regular diet during these twelve weeks. This is important for the reliability of the research results. Also note the following:

- Your appetite may change as a result of the exercise. However, try to maintain the same quality of your diet. Pay special attention to avoid an increase in the number of sugary drinks and snacks you consume. Do not forget to drink water immediately after exercising to maintain your fluid balance.

- Medication use must remain unchanged during the study. Contact your researcher if there are any changes in your medication.

- Try to keep your weight constant (try not to lose or gain weight).

In addition, you will be asked 3 times to fill out an online food diary: before week 0, week 6 and week 12, for 4 or 5 days (3-4 working days and 1 weekend day which will be predefined) via https://mijn.voedingscentrum.nl/nl/eetmeter. See Appendix B for food diary instructions.

Questionnaires and Polar Active: We will ask all participants to complete a physical activity questionnaire, which measures your activity during these 3 months. In addition, you will wear a Polar Active activity monitor on your wrist during these 12 weeks. Make sure you take the watch with you every time you come to the exercise training, so that it can be read.

Continous glucosemeter: (FreeStyle Libre): we do not expect this to be burdensome for participants. The FreeStyle Libre glucose meters are meters that are placed on the skin, with a very small needle just under the skin (not in the bloodstream). While wearing it you can do everything you are used to doing, such as showering, exercising, etc. We will use the glucose meters to get an estimate of the daily fluctuation in your blood glucose concentration. Glucose monitoring will be done during 2 weeks prior to the sports intervention and during the last 2 weeks. We will ask you to perform 8-10 scans per day. It is important to regularly hold a sensor over the meter, otherwise the sensor will stop recording. Click here for more information about the meters: https://www.youtube.com/watch?v=_pcgmCpNPCw. This is a short YouTube video showing how the gauges are applied and used.

Blood collection: A total of 3 x 60 ml of blood will be drawn during the study visits. We do not expect any complications from blood drawing as it is standard procedure. However, placing the intravenous cannula can be an unpleasant experience in this study. There is a low risk of phlebitis (inflammation of the veins) at the intravenous injection sites, which is unpleasant, but not harmful, temporary and usually self-limiting.

MRI examination: MRI stands for Magnetic Resonance Imaging. We use the MRI equipment to make cross-sectional pictures of your liver, abdomen and bowel motility. You must be sober for this examination.

Liver biopsy: All participants will be referred to the AMC interventional radiology department for liver biopsy. An interventional radiologist will perform an ultrasound-guided liver biopsy. Ultrasound-guided percutaneous liver biopsy has a very low risk of complications (<0.1%), mainly consisting of bleeding from the biopsy site. Prior to the biopsy, the skin at the biopsy site is numbed. Despite this, there may be pain at the biopsy site that can last for several hours. Sometimes multiple biopsies are needed. After the procedure, you will be observed to ensure that local blood clotting is proceeding properly. A bandage will be placed over the puncture site. After the biopsy, participants should lie on their right side for 1-2 hours. You cannot participate in the study if you have an increased tendency to bleed or if you are taking anticoagulant medication.

Adipose tissue biopsy: First of all, the abdominal skin is anesthetized at the site of the biopsy. The anesthetic is a small injection and can feel a bit uncomfortable. Because of this anesthesia you will not feel any pain during the biopsy. After this, a small amount of fatty tissue (about 1 gram) will be aspirated from the subcutaneous fat layer with a hollow needle. The puncture hole in the skin will be covered with a plaster. In the hours following the biopsy, the biopsy site may feel a little sore and a bruise may develop. This will disappear over time.

Muscle biopsy: The muscle biopsy will be performed under local anesthesia. After a muscle biopsy, your leg may feel tender for a few days. It is a minor procedure, usually without complications, but as with any procedure, there is a small risk of bleeding or infection.

**6. Possible advantages and disadvantages**

It is important that you carefully weigh the possible advantages and disadvantages before you decide to participate in this study.

Possible benefit of participation:

Participation in the study can lead to better physical fitness. It can also lead to better overall health. You also contribute to a better understanding of how long-term physical exercise contributes to better metabolic health of the liver, muscle and adipose tissue. This could possibly result in new (intestinal bacteria based) treatments.

Disadvantages of participating in the study can be:

- Possible complications of biopsies

Participation in the study also means:

- That you lost extra time;

- (Additional) testing;

- That you have agreements that you must adhere to;

All these matters have been described above under points 4, 5 and 6.

**7. What happens if you do not want to participate in this study?**

You decide whether to participate in the study. Participation is voluntary. If you decide not to participate, you don't need to do anything else. You don't have to sign anything. You also don't have to say why you don't want to participate. If you are a patient, you will simply receive the treatment you would otherwise receive. If you participate, you can stop at any time during any step of the study.

**8. End of the study**

Your participation in the study will stop in the following situations

• You choose to stop yourself

• The end of the full investigation has been reached

• The examiner thinks it would be better for you to stop

• The AMC, the government or the assessing medical ethics review committee decides to stop the research.

The entire study is complete when all participants have completed the study. After processing all data, the researcher will inform you about the most important results of the research. This will happen approximately one year after your participation.

**9. Use and storage of your data and bodily material**

Your personal data and bodily material are collected, used and stored for this research. This concerns data such as your name, address, gender, date of birth, ethnicity, and data about your health. Blood, urine, feces, and biopsies are required for this study. The collection, use and storage of your data and your bodily material is necessary to answer the questions asked in this study and to publish the results. We ask for your permission for the use of your data and bodily material.

**Confidentiality of your data and bodily material**

To protect your privacy, your data and your bodily material are assigned a code. Your name and other data that can directly identify you are omitted. Data can only be traced back to you with the key of the code. The key to the code remains safely stored in the Amsterdam UMC, location AMC. The data and bodily material sent to the cooperating unit in Finland and Germany contain only the code, but not your name or any other identifying information. The data in reports and publications about the research cannot be traced back to you either.

**Access to your data for verification**

Some individuals will be able to access to all of your data at the study site. Also to the uncoded data. This is necessary in order to check whether the research has been carried out properly and reliably. These persons will keep your data secret. We ask you to give permission for this inspection. Persons who will have access to your data for verification purposes are:

- Inspectors on behalf of the client, the Amsterdam UMC, location AMC

- National supervisory authorities, for example, the Health and Youth Care Inspectorate

**Retention period for data and bodily material**

Your data must be kept in the AMC for 15 years. Your bodily material will be kept for 5 years in order to be able to repeat failed determinations or to make new determinations related to this research.

**Storage and use of data and bodily material for future research**

After this study, your data and bodily material may also be important for other scientific research in the field of NAFLD. For this purpose, your data will be kept for 15 years and your bodily material for 15 years. For information and permission for longer storage (maximum 15 years) for future research, please refer to the separate information letter and consent statement for the biobank associated with this study.

**Information about unexpected findings**

During this investigation, something may be found by chance that is not important for the investigation, but is important for you. If this is important to your health, you will be notified. You can then discuss with your GP or specialist what needs to be done. You also give permission for this.

**Revoke your consent**

You can always withdraw your consent to the use of your personal data. That applies to this research as well as for the storage and use of your bodily materials for future research. The research data collected until you withdraw your consent will still be used in the research. Your bodily material will be destroyed when you withdraw your consent. If measurements have already been done using your bodily material, this data will still be used.

**Transfer to countries outside the European Union (EU)**

In this study, your coded data and bodily material will also be forwarded to countries outside the EU, because the genetic analysis of your intestinal bacteria, liver tissue, adipose tissue and muscle biopsies will take place at Novogene in China. In those countries, the rules of the EU for the protection of your personal data do not apply. However, your privacy will be guaranteed because we will only send samples with your coded data (entry number) to Novogene, which Novogene cannot trace back to you. The final link with clinical data will take place at Amsterdam UMC.

**Learn more about your rights when processing data**

For general information about your rights when processing your personal data, you can consult the website of the Dutch Data Protection Authority:

<https://autoriteitpersoonsgegevens.nl/>

If you have any questions about your rights, please contact the person responsible for the processing of your personal data. For this research this is: Prof. dr. M. Nieuwdorp, who works at the Academic Medical Center (AMC), e-mail: [m.nieuwdorp@amsterdamumc.nl](mailto:m.nieuwdorp@amsterdamumc.nl).

If you have any questions or complaints about the processing of your personal data, we recommend that you first contact the research location. You can also contact the data protection officer of the institution via fg@amc.nl or the Dutch Data Protection Authority.

**Registration of the research**

Information about this research is also included in an overview of medical-scientific studies, namely www.toetsingonline.nl. No data is included that can be traced to you. After the study is finished, this website may display a summary of the study results.

**10. Are you insured when you participate in the study?**

An insurance policy will be taken out for everyone who participates in this study. The insurance covers damage as a result of the investigation. This applies to damage that has arisen during the research or within four years after the end of the research. You will find the insurance text in Appendix G.

**11. Information to your general practitioner (GP)**

We will always send a letter to your GP to let them know that you are taking part in the study. This is for your own safety. If abnormalities are found during the test (for example in the blood) that are important for your health, we will inform your GP and yourself. If you do not want this, you cannot participate in this study. You cannot participate in the study if you do not have a doctor.

**12. Is there a compensation if you decide to participate in this study?**

You will receive 350 euros for participating in the study and an allowance for travel expenses. In addition, you will receive free participation in a cardio fitness program during the study.

**13.** **Who can I contact with questions about this study?**

For all questions about the study and your rights or if an accident occurs in connection with the study, please contact the study doctor, Prof. dr. M. Nieuwdorp, internist. For any questions you may have regarding the study, you may also contact the independent physician involved in this study, D. M. Cohn, or the undersigned. All contact information can be found in Appendix D.

**14. Consent form**

After you have had sufficient time to think, you will be asked to decide whether you wish to participate in this study. If you give permission, we ask you to confirm this in writing on the accompanying statement of consent (Appendix J). With your written consent, you indicate that you have understood the information and agree to participate in the study. Both you and the researcher will receive a signed version of this consent form.

**15. Will you be kept informed when new developments become known about the study?**

The investigation will proceed as closely as possible according to plan. But the situation can change. For example, through the reaction of your body or through new information. Then we will discuss this directly with you. You then decide whether you want to stop or continue with the study. If your safety or well-being is at risk, we will stop the investigation immediately.

**16. Which medical ethics review board approved this study?**

The Medical Ethical Review Committee (METC) of the AMC has approved this study. More information about the approval can be found in the general brochure for scientific research.

Sincerely, on behalf of drs V.A.T Houttu, researcher

dr A.G. Holleboom, internist

Department of Vascular Medicine

Amsterdam University Medical Centers, location Amsterdam

email: [a.g.holleboom@amsterdamumc.nl](mailto:a.g.holleboom@amsterdamumc.nl)

**17. Attachments**

A. Instructions for preparing laboratory samples

B. Diet diary instructions

C. Morning Stool Collection Instructions

D. 24-H Urine Collection Instructions

E. Exercise Test Instructions

F. Instructions for the training sessions

G. Insurance information

I. Algemene brochure medisch-wetenschappelijk onderzoek met mensen

J. Contactgegevens

K. Verklaring van toestemming

**Appendix A: Instructions for preparing blood samples**

When asked to come sober for one of the study visits, please adhere to the following instructions:

- Keep your eating habits the same prior to visits. Avoid large meals, alcohol, sweets or snacks (e.g. a bar of chocolate or a bag of chips) 24 hours prior to the visits.

- Limit eating, chewing gum or lozenges 12 hours prior to visit. During fasting, water (1 glass of water in the evening and 1 glass on the morning of the test) is allowed, but coffee, tea, milk or juice are not allowed.

- You may simply take your prescribed home medication the morning of the examination (with water!). However, there is 1 exception: you may only take thyroid medication (Thyrax) after the morning blood tests!

You are not allowed to smoke on the morning of the examination.

On the morning of the examination, if possible, come by car or public transport to limit physical strain as much as possible (so don't come by bike). We ask you to arrive 15 minutes early for each visit, so that your blood circulation can calm down.

Avoid any major physical exertion 24 hours prior to the blood draw and/or liver biopsy.

Blood and/or plasma donation during the study is not allowed and there must be a donation-free period of at least 1 month before the first visit.

If you are ill (e.g. flu/severe cold), please contact the researcher. Infections can have major effects on research results. The visit may then have to be postponed.

**Appendix B: Food diary instructions**

For the CRISTINA study, you will collect data about your diet for five days before the study visits. For this we use the 'Eetmeter' of the Nutrition Center. Below you will find instructions for the online food diary.

- Keep the food diary for at least 4-5 consecutive days a week before and after the study visit.

o Make sure at least 1 day falls on a weekend

o Collecting more than 4-5 days in the food diary is always allowed.

- Write down everything you eat and drink, preferably immediately after consumption so that you don't forget anything.

- Describe the dishes you eat as accurately as possible (e.g. all individual ingredients in salads and sauces).

- Record the quantity of each food item as accurately as possible (in grams, decilitres, tea/dining/serving spoons, slices, etc.).

- Also note the quality as accurately as possible (e.g. semi-skimmed milk, cheese with 24% fat, wholemeal bread, etc.).

- Take into account the method of preparation (oven, baked, boiled) and the use of fats and oils in baking.

Create an account

1. Go to https://mijn.voedingscentrum.nl/

2. Click on register and enter your details here.

3. Activate your account.

Complete a diet diary

1. Log in with your login details: https://mijn.voedingscentrum.nl/nl/login/. The 'Eat Meter' can also be downloaded as an app on your smartphone.

2. Below is an overview of the interface.

3. Here you write down everything you eat and drink during breakfast, lunch, dinner and in between.


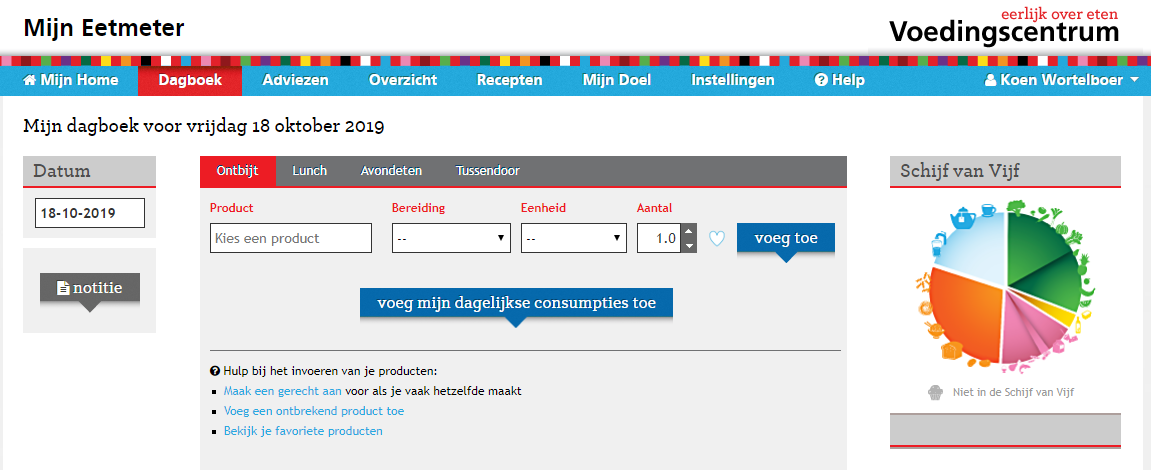


1. Add product or ingredient:

a. Look up the product or ingredient. Pay attention to the quality (wholemeal bread, skimmed milk, etc.)

b. If applicable: select a cooking method (boiled, baked, etc.)

c. Choose a unit (gram, serving spoon, etc.)

d. Choose a number (how many grams/serving spoons)

2. You can remove products by clicking on the red cross to the right of the products.

3. If desired, you can add a note to the day.


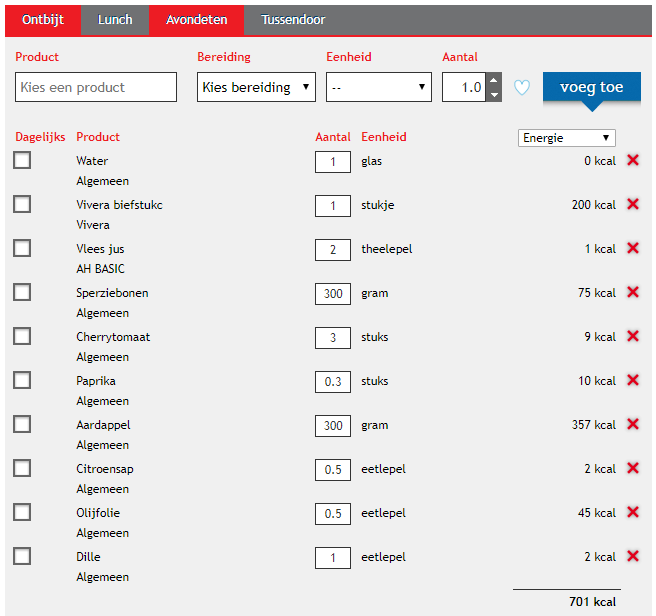
4. Below is an example of a completed diary.
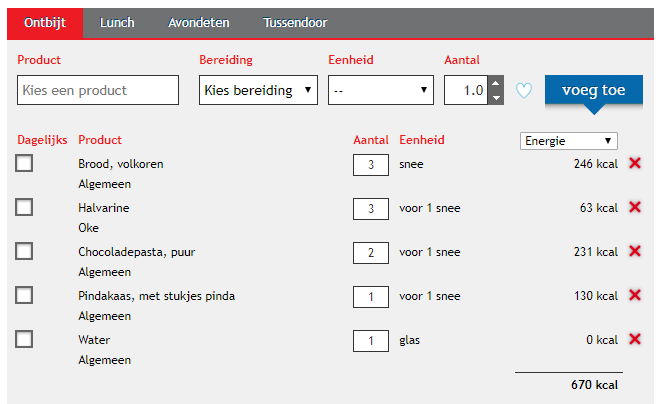


**Export and send data**

After collecting the data (3 x 4-5 days), we ask you to send us your diet diary. For this you need to download your diet diary and send it to the researcher by e-mail. We can do the visit together. You do this as follows:

1. Go to “Overview” and select the correct week number(s) under “date”. (see below)

2. Press the “excel” button to download the overview in Excel format.

3. Send the data by email to the researcher: v.a.houttu@amsterdamumc.nl
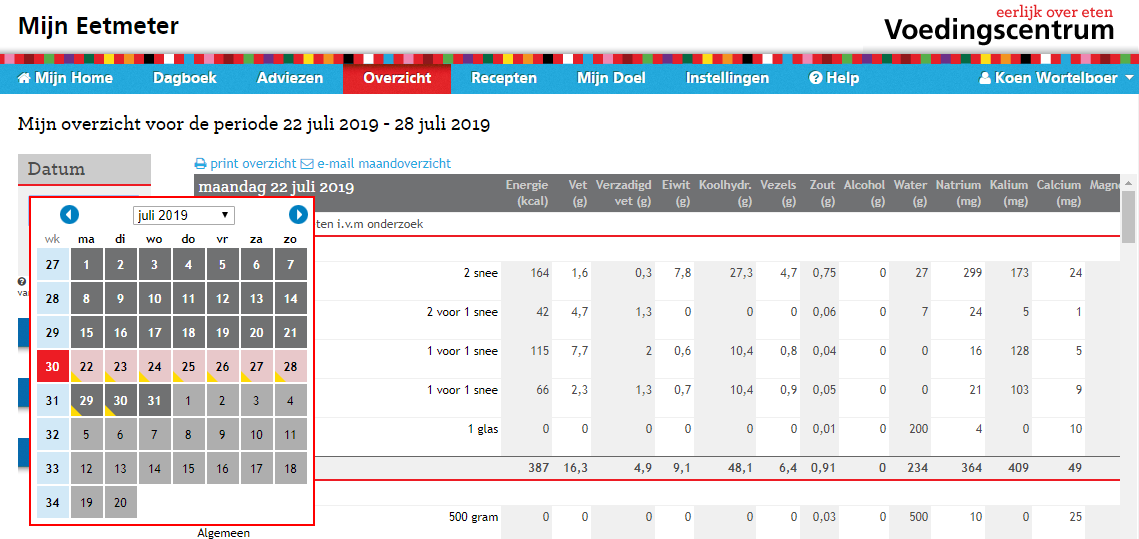


**Appendix C: Morning Stool Collection Instructions**

For the CRISTINA study, we ask that you collect morning stool before your visit. Stool is collected before, midway through and after the intervention. Below are instructions for collecting the stool.


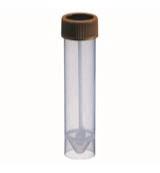

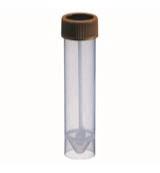

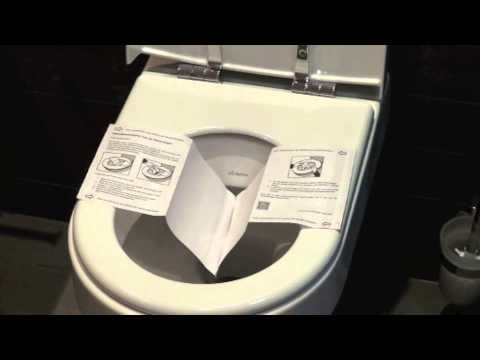
**The** **package contains the following**


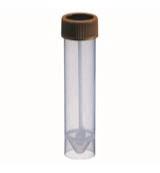
- Spoon tubes with brown cap 3x

- Feces catcher 1x

- Instructions for collection

**Verzameling ochtend ontlasting**

1.
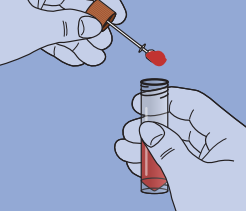

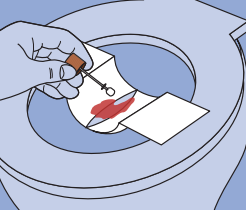
Collect your faeces in the toilet using the faeces catcher. Follow the instructions on the feces trap.
2.
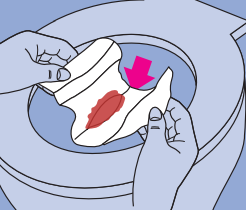

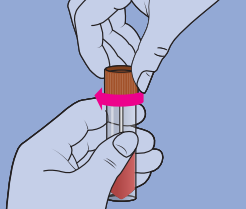
Fill in three spoon tubes with faeces using the spoon attached to the cap. Fill the tube (maximum) halfway with faeces.Screw the cap tightly onto the spoon tubes.

1

2

1. The used faeces catcher can be flushed down the toilet.
2. Attention: all tubes already contain a label with your data. Preferably hand in the tubes as soon as possible (see below).

4

3

**Collection and delivery of faeces**

- Collect your stool as directed above on the morning before your first, midway and final study visit.

- After filling the spoon tubes, they should be taken to the study visit. The tubes can be temporarily stored at room temperature.

- If you cannot collect stool in the morning, you can collect the stool a day in advance. Then keep the spoon tubes in the fridge and don't forget to take them with you to the study visit.

**Appendix D: Instructions for 24-Hour Urine Collection**

We ask that you collect 24-hour urine before your visit. Below are the instructions for collecting 24-hour urine. For this you use the ureter and the urethra.


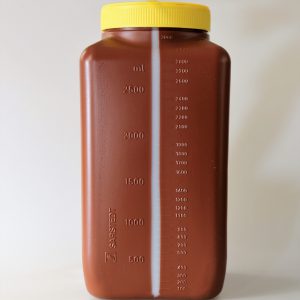


**Het pakket bevat het volgende**

- Urine container 1x
-
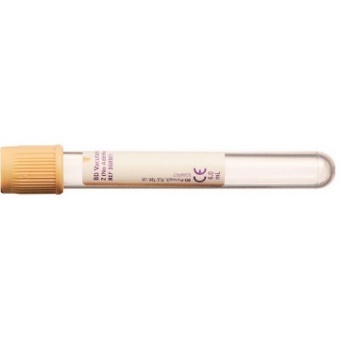

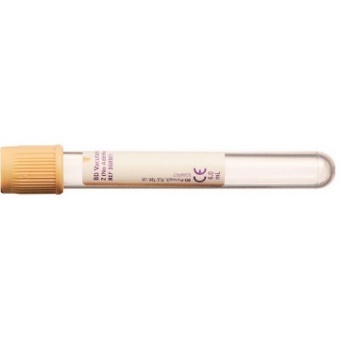

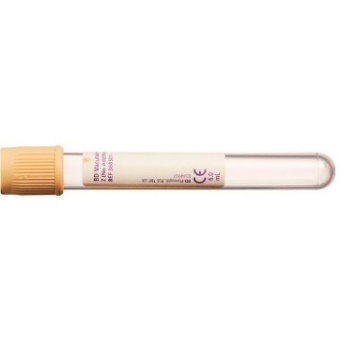
Urinetubes 3x
- Collection instructions and kit

**Collection**

1. Empty your bladder the morning before the study visit (24 hours before the scheduled visit). Do not collect this urine. Record the date and time of urination (start date).

Start date:_______ Time:______

2. Then urinate in the jar all day (24 hours), evening and night. Be careful not to lose urine, for example with the stool. Try to keep the container as cool as possible during the day. However, do not let the urine freeze.

3. The next morning (24 hours after start) collect urine for the last time in the collection container. Record the date and time of last urination (end date) and volume. The jar can hold three liters, which is usually sufficient. When the jar is full, you may stop collecting.

End date:_______ Time:______ Volume:_____

4. Remove the sticker from the lid of the jar. Hold the jar upside down and then insert the urine tube one by one into the opening in the lid. The tubes fill up automatically.

5. After the urine tube has been filled, it should be taken with you to the study visit. The tubes can be temporarily stored at room temperature.

6. Please also bring the urine cup with you to the study visit.

**Appendix E: Exercise Test Instructions**

The exercise test (ergo spirometry) examines the functioning of the heart and lungs and determines the maximum effort. The test takes place on a spin bicycle using heart activity measurement (ECG), blood pressure measurement and gas exchange measurement. You do it twice, namely before and after the training period of 12 weeks at Polfysiek (Hogeschool van Amsterdam).

Preparing for the test

When preparing for the test, keep the following in mind:

- Avoid strenuous exercise on the day before the examination and certainly at least on the day of the examination itself.

- Just eat your breakfast and take your regular medicines. However, two hours before the test, you should not eat a heavy meal or drink coffee, tea or cola drinks, but you can drink other liquids.

- The use of alcohol is not recommended for two days before ergo spirometry, because alcohol and the slight hangover have a strong influence on the heart.

- Avoid smoking 12 hours before the test; In any case, you are not allowed to smoke for four hours before the examination.

- Wear or bring: shorts or sportswear with a towel (changing room available). Women can wear a very loose sleeveless T-shirt

Location of Polyphysics

Polifysiek is located close to Amsterdam UMC on the Health Campus of the Amsterdam University of Applied Sciences. The address of Polifysiek is Tafelbergweg 51, 1105 BD Amsterdam.

The entrance to the Amsterdam University of Applied Sciences is on Tafelbergweg, past the golf course just before the viaduct onto the campus (through the barriers, sign up for Polifysiek, free entrance).

**Appendix F: Instructions for the training sessions**

The training is performed twice a week according to an individual, ascending program on the exercise bike. You do the training in small groups at Polfysiek, Amsterdam University of Applied Sciences (HvA). This HIIT training consists of the following parts: A: Warming up of 10 minutes (low intensity), B: 5 x intensive blocks (HIIT training) with 5 x 3 minutes rest in between and C: Cooling down of 5 minutes (low intensity). The intensity of the training is determined by the outcome of the exercise test at the start.

Before workout

When preparing for your training session, keep the following in mind:

- Take your medicines that you use regularly.

- You should not eat a heavy meal two hours before exercising, but you can drink anything. Fasting beforehand is also not allowed.

- Avoid smoking 12 hours before exercise; In any case, you should not smoke four hours before exercising.

- Bring shorts or sportswear, T-shirt and towel for training. A dressing room is available

- Please note: if you are ill or absent: Contact the researcher VAT Houttu (v.a.houttu@amsterdamumc.nl), if possible a new appointment will be scheduled.

Location of Polyphysics

Polifysiek is located close to Amsterdam UMC on the Health Campus of the Amsterdam University of Applied Sciences. The address of Polifysiek is Tafelbergweg 51, 1105 BD Amsterdam.

The entrance to the Amsterdam University of Applied Sciences is on Tafelbergweg, past the golf course just before the viaduct onto the campus (through the barriers, sign up for Polifysiek, free entrance).

**Freestyle Libre scanner device**


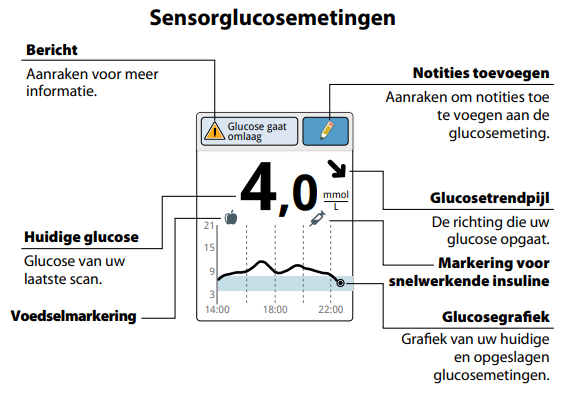

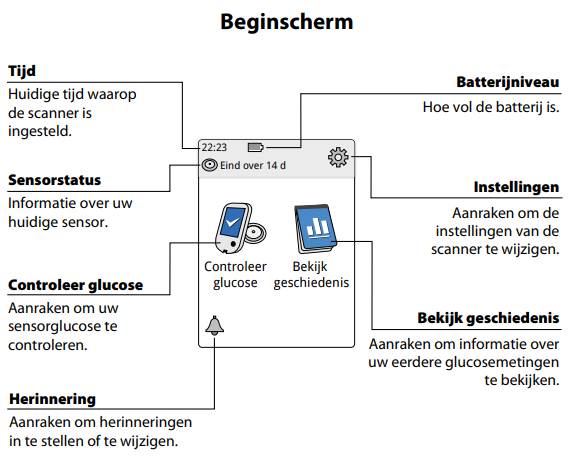


**Applying the sensor**


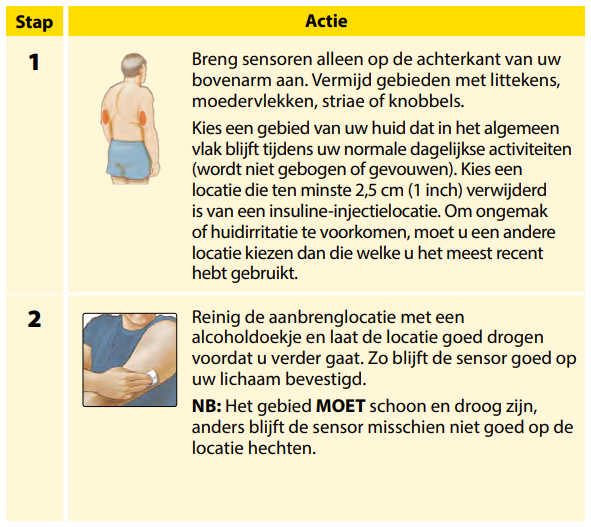

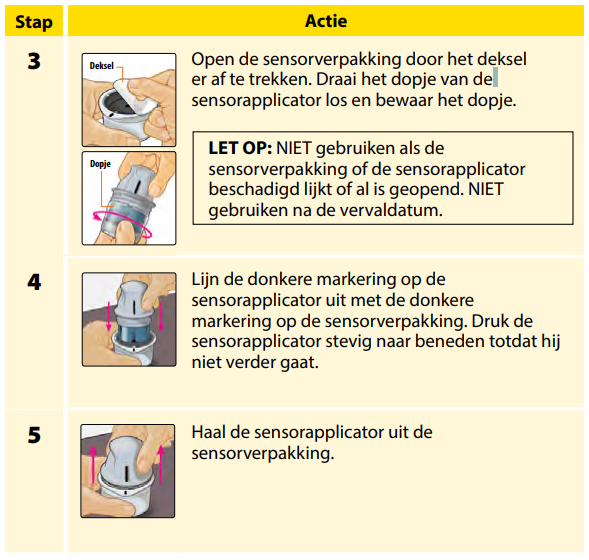


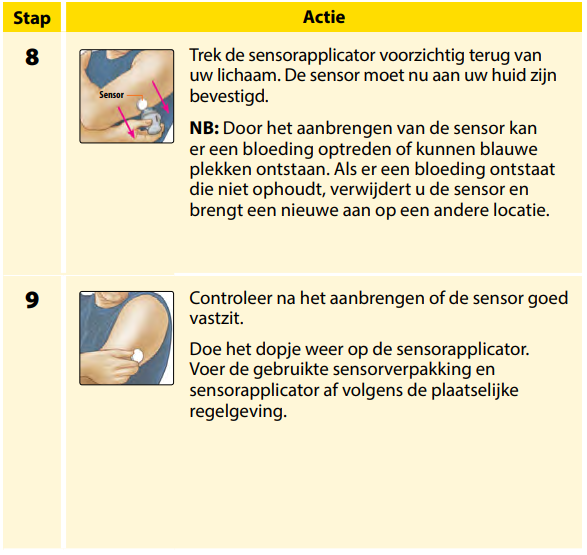


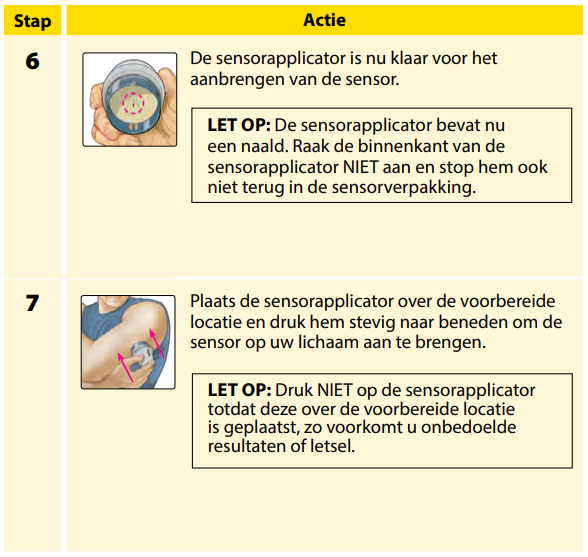


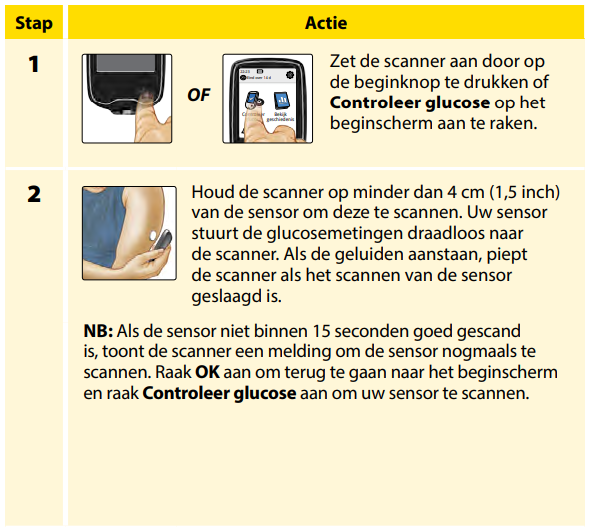
**Start the sensor Measure blood sugar**


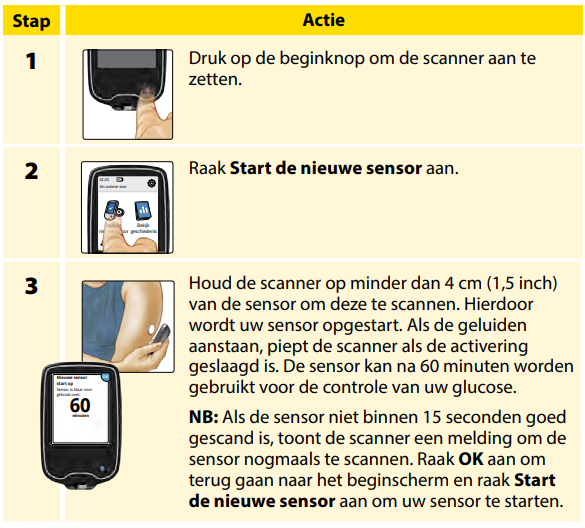


Remarks

- The sensor can store up to 8 hours of data. Therefore, you should scan the sensor at least every eight hours.

o Our advice is to scan the sensor before you go to sleep and when you wake up.

o We also recommend scanning the sensor during every meal.

- It is possible to scan the sensor through clothing (less than 4 cm).

- A fully charged scanner battery should work for up to 7 days. If the battery is almost empty, you can charge it with the supplied cable. This takes about 3 hours.

Remove sensor

- Pull up the edge of the adhesive that adheres your sensor to your skin.

- Pull the sensor from your skin in one slow movement.

**Annex I: Information about the insurance**

AMC has taken out insurance for everyone who participates in this study. The insurance covers damage caused by participation in the study. This applies to damage during the study or within four years after the end of your participation in the study. You must report damage to the insurer within those four years.

The insurance does not cover all damage. At the bottom of this text you will find a brief description of which damage is not covered.

These provisions are contained in the Compulsory Insurance Decree for Medical Research involving Human Subjects. This decision can be found on www.ccmo.nl, the website of the Central Committee on Research Involving Human Subjects (see 'Library' and then 'Laws and regulations').

In the event of damage, you can contact the insurer [or claims representative] directly.

The insurer of the study is:

Name: Centramed B.A.

Address: PO Box 7374, 2701 AJ Zoetemeer

Telephone number: 070 301 70 70

Email: info@centramed.nl

Policy number: 624.528.303

The insurance offers cover of € 650,000 per test subject with a maximum of € 5,000,000 for the entire research and € 7,500,000 for damage resulting from medical research that is reported per policy year.

The insurance does not cover the following damage:

• damage due to a risk about which you have been informed in the written information. This does not apply if the risk is more serious than anticipated or if the risk was very unlikely;

• damage to your health that would also have occurred if you had not participated in the study;

• damage due to not (completely) following directions or instructions;

• damage to your descendants, as a result of a negative effect of the research on you or your descendants;

• damage caused by an existing treatment method in research into existing treatment methods.

Furthermore, the test subject is requested to contact Prof. Dr. M. Nieuwdorp, 020-5665737**Appendix J: Contact information**

Researcher

V. A. T. Houttu, promovenda

Afdeling Vasculaire Geneeskunde

Amsterdam UMC, locatie AMC

Tel: 020 5665158

Email: [v.a.houttu@amsterdamumc.nl](mailto:v.a.houttu@amsterdamumc.nl)

Research physician

Dr. A.G. Holleboom, MD PhD

Afdeling Vasculaire Geneeskunde

Amsterdam UMC, locatie AMC

Tel: 020 5661925

Email: [a.g.holleboom@amsterdamumc.nl](mailto:a.g.holleboom@amsterdamumc.nl)

Research nurse

D. Zwirs

Afdeling Vasculaire Geneeskunde

Amsterdam UMC, locatie AMC

Tel: 020 5666638

Email: [d.zwirs@amsterdamumc.nl](mailto:d.zwirs@amsterdamumc.nl)

Principal investigator

Prof. dr. M. Nieuwdorp

Afdeling Vasculaire Geneeskunde

Amsterdam UMC, locatie AMC

Tel: 020 5665737

Email: [m.nieuwdorp@amsterdamumc.nl](mailto:m.nieuwdorp@amsterdamumc.nl)

Independent physician

D. M. Cohn

Afdeling Vasculaire Geneeskunde

Amsterdam UMC, locatie AMC

Tel: 020 5668274

Email: [d.m.cohn@amsterdamumc.nl](mailto:d.m.cohn@amsterdamumc.nl)

Data Protection Officer

Mw. mr. J.B.M. Inge, [fg@amc.nl](mailto:fg@amc.nl)

**Appendix K: Subject consent form**

CRISTINA study

- I have read the information letter. I could also ask questions. My questions have been answered satisfactorily. I had enough time to decide whether to participate.

- I know that participation is voluntary. I also know that I can decide at any time not to participate or to stop the study. I don't have to give a reason for that.

- I give permission for my GP to be informed that I am participating in this study.

- I give permission for my general practitioner and/or treating specialist to be informed of unexpected findings that are or could be important for my health.

- I consent to the collection and use of my data, including documentation of ethnicity, blood samples and bodily material to answer the research question in this study.

- I know that for the purpose of checking the investigation, some people may have access to all my data. These people are listed in this information letter. I consent to such access by these persons.

-I give □ yes □ no

permission to keep my personal data longer and to use it for future research in the field of NAFLD.

- I give □ yes □ no

permission to store my bodily material for 5 years after this research for provisions related to this research, as described in the information letter.

- I give □ yes □ no

permission to approach me again after this investigation for a follow-up investigation.

- I want to participate in this research.

Name:

-------------------------------------------------------------------------------------------------------------------------

Signature: Date : __ / __ / ____

-------------------------------------------------------------------------------------------------------------------------

I declare that I have fully informed this subject about the said study. If information becomes known during the research that could influence the subject's consent, I will inform him/her in good time.

Name of researcher (or his representative):

-------------------------------------------------------------------------------------------------------------------------

Signature Date: __ / __ / ____

-------------------------------------------------------------------------------------------------------------------------

The subject receives a complete information letter, together with a signed version of the consent form.
