# Supplementary material for: Deep phenotyping of patients with MASLD upon high-intensity interval training
Source: JHEP Rep. 2024 Dec 16;7(3):101289. doi: 10.1016/j.jhepr.2024.101289 (PMC11883402; doi:10.1016/j.jhepr.2024.101289)
Supplement: Multimedia component 5 [file mmc5.zip › Clinical Trial/ENGLISH translation ethics approval.docx]

ENGLISH translation

----

Amsterdam, September 9, 2020

our reference: 2019_061#B2020493

concerns: Positive further assessment

NL69349.018.19

An exercise intervention study in NAFLD patients

Dear Mr Nieuwdorp,

The METC AMC has, on the basis of Article 2, paragraph 2, sub a of the Medical Research Act

consult with people (WMO) about the amendment associated with the above-mentioned research file. We are pleased to inform you that our committee

- authorized to make judgments pursuant to Article 2, paragraph 2, under a, of the Medical-Scientific Act

research with people (WMO);

- works according to the ICH-GCP guidelines;

- on the basis of the documents submitted to it as stated below;

- in view of Article 3 of the WMO;

- having regard to Articles 5 and 6,

has decided on a positive further assessment of this protocol and its implementation in the AMC.

Documents involved in the assessment:

A1 offer letter dated July 8, 2020

A1 offer email dated July 10, 2020

A1 offer email dated August 24, 2020

A1 offer letter dated August 24, 2020, unsigned

A1 offer email dated September 1, 2020

A1 offer email dated September 9, 2020

B1 ABR form NL69349.018.19 version 05 dated July 8, 2020

C1 protocol version 5.0 dated July 8, 2020

E1 E2 subject information and consent statement version 9.0 dated July 8, 2020

E1 E2 new information participating test subjects version 1.2 dated September 9, 2020 TC

The amendment, submitted to us for assessment on July 10, 2020, was discussed at the meeting of the executive board of our committee of July 22, 2020. Further handling is mandated to the

secretary. This has established that when the amended documents were submitted on August 24

2020, September 1, 2020 and September 9, 2020, the committee's request has been met. The amendment concerns, among other things, correction of an exclusion criterion and the collection of data on ethnicity and adjustments.

We would like to point out that pursuant to Article 23 of the Medical Research Involving Human Subjects Act the person whose interest is directly involved in a decision of the METC, within six weeks

can submit an administrative appeal to the Central Office on the day on which the decision is announced Committee on Human Research. Such an administrative appeal must be addressed to:

to: CCMO, PO Box 16302, 2500 BH The Hague.

Perhaps unnecessarily, the METC points out that the obligations included in the original positive decision stated, remain in full force.

For the exact composition of the committee at the meeting in which the decision was made, please

contact the committee secretariat.

Yours sincerely,

on behalf of the Medical Ethical Review Committee AMC,

Mrs. T. Groenveld,

Secretary

As long as the restrictive measures due to the coronavirus apply, the decisions of the METC

not be provided with a wet signature. The decisions are sent digitally. If after the

If the withdrawal of the measures still requires a signed decision, the METC would like to hear this.

c.c. CCMO (pdf via TOL)

c.c. pdf by email A.G.Holleboom, V.A.T.Houttu, N.Sons
