# Supplementary material for: Deep phenotyping of patients with MASLD upon high-intensity interval training
Source: JHEP Rep. 2024 Dec 16;7(3):101289. doi: 10.1016/j.jhepr.2024.101289 (PMC11883402; doi:10.1016/j.jhepr.2024.101289)
Supplement: Multimedia component 5 [file mmc5.zip › Clinical Trial/P2 B2020493 d.d. 9 september 2020.pdf]

Amsterdam UMC
Universitair Medische Centra

Aan de heer prof.dr. M. Nieuwdorp
Inwendige geneeskunde
F4-159-2

Medisch Ethische Toetsingscommissie AMC
TK0-270

Amsterdam, 9 september 2020
ons kenmerk: 2019_061#B2020493
betreft: **Positief nader oordeel**
NL69349.018.19
An exercise intervention study in NAFLD patients

Geachte heer Nieuwdorp,

De METC AMC heeft zich, op grond van artikel 2, lid 2, sub a van de Wet medisch-wetenschappelijk onderzoek met mensen (WMO) beraden over het amendement behorend bij bovengenoemd onderzoeksdossier.
Wij delen u gaarne mee dat onze commissie

- tot oordelen bevoegd krachtens artikel 2, tweede lid, onder a, van de Wet medisch-wetenschappelijk onderzoek met mensen (WMO);
- werkzaam volgens de ICH-GCP richtlijnen;
- op grond van de haar voorgelegde stukken als hierna vermeld;
- gelet op artikel 3 van de WMO;
- gelet op artikel 5 en 6,

heeft besloten tot een positief nader oordeel over dit protocol en de uitvoering daarvan in het AMC.

In de beoordeling betrokken documenten:
A1 aanbiedingsbrief d.d. 8 juli 2020
A1 aanbiedingsemail d.d. 10 juli 2020
A1 aanbiedingsemail d.d. 24 augustus 2020
A1 aanbiedingsbrief d.d. 24 augustus 2020, ongetekend
A1 aanbiedingsemail d.d. 1 september 2020
A1 aanbiedingsemail d.d. 9 september 2020
B1 ABR-formulier NL69349.018.19 versie 05 d.d. 8 juli 2020
C1 protocol versie 5.0 d.d. 8 juli 2020
E1 E2 proefpersoneninformatie en toestemmingsverklaring versie 9.0 d.d. 8 juli 2020
E1 E2 nieuwe info deelnemende proefpersonen versie 1.2 d.d. 9 september 2020 TC

Het amendement, aan ons ter beoordeling voorgelegd op 10 juli 2020, is besproken in de vergadering van het dagelijks bestuur van onze commissie van 22 juli 2020. De verdere afhandeling is gemandateerd aan de secretaris. Deze heeft geconstateerd dat met het voorleggen van de aangepaste stukken d.d. 24 augustus 2020, 1 september 2020 en 9 september 2020 aan het verzoek van de commissie is voldaan. Het amendement betreft onder meer correctie van een exclusiecriterium en het verzamelen van gegevens over etniciteit en aanpassingen.

Wij wijzen u erop dat op grond van artikel 23 van de Wet medisch-wetenschappelijk onderzoek met mensen degene wiens belang rechtstreeks bij een besluit van de METC is betrokken, daartegen binnen zes weken na de dag waarop het besluit bekend is gemaakt, een administratief beroepschrift kan indienen bij de Centrale Commissie Mensgebonden Onderzoek. Een dergelijk administratief beroepschrift dient geadresseerd te worden aan: CCMO, Postbus 16302, 2500 BH Den Haag.

AMC
Meibergdreef 9
1105 AZ Amsterdam
Postbus 22660
1100 DD Amsterdam
T +31(0)20 566 9111
www.amc.nl

Wellicht ten overvloede wijst de METC erop dat de verplichtingen die bij het oorspronkelijke positieve besluit zijn vermeld, onverminderd van kracht zijn.

Ten tijde van de beoordeling was de commissie als volgt samengesteld:

prof.dr. J.A. Swinkels	:	voorzitter, psychiater
mw.drs. G.H.M. van Ammers	:	lid dat onderzoek beoordeelt vanuit de invalshoek van de proefpersoon
mw. dr. E.C.A. Asscher	:	medisch ethicus
dr. H. van den Berg	:	lid kinderarts
dr. P.M. Bet	:	plv. lid ziekenhuisapotheker, klinisch farmacoloog
mw.prof.dr. M.A. Boermeester	:	chirurg
prof. dr. A.J. Bredenoord	:	maag darm lever arts
prof.dr. M.G.W. Dijkgraaf	:	plv. lid, methodoloog
mw. J.M.M. Dijkstra	:	lid dat onderzoek beoordeelt vanuit de invalshoek van de proefpersoon
prof.mr.dr. J.K.M. Gevers	:	plv. jurist
prof.dr J.W. Groothoff	:	plv. kinderarts
dr.ir. J.M. den Harder	:	klinisch fysicus
mw.dr. M.D. Hazenberg	:	internist-hematoloog
dr. J.Ph. de Jong	:	plv. lid, ethicus
dr. R.E. Jonkers	:	longarts/plv. lid klinisch farmacoloog
dr. M.J.W. Koelemay	:	vaatchirurg
mw.mr. E.J. Kranendonk	:	jurist
prof.dr. R.A.A. Mathôt	:	ziekenhuisapotheker, klinisch farmacoloog
dr. J.T.M. van der Meer	:	internist-infectioloog
dr. P.J Nederkoorn	:	neuroloog
mw.mr. W. Paping-Kool	:	plv. jurist
prof.dr. A.J.P.M. Smout	:	maag darm lever arts
mw.mr. L.M. Spittuler	:	lid dat onderzoek beoordeelt vanuit de invalshoek van de proefpersoon
prof.dr. J. Stam	:	neuroloog
dr. H.L. Tan	:	cardioloog
prof.dr. J.G.P. Tijssen	:	plv. lid, klinisch epidemioloog
mw.dr. A.M. Westermann	:	internist-oncoloog
prof.dr. D.L. Willems	:	plv. medisch ethicus
prof.dr. A.H. Zwinderman	:	biostatisticus.

Voor de exacte samenstelling van de commissie tijdens de vergadering waarin het besluit is genomen, kunt u contact opnemen met het secretariaat van de commissie.

Met vriendelijke groet,
namens de Medisch Ethische Toetsingscommissie AMC,

Mw. T. Groenveld,
ambtelijk secretaris

Zo lang de beperkende maatregelen als gevolg van het coronavirus gelden zullen de besluiten van de METC niet worden voorzien van een natte handtekening. De besluiten worden digitaal verstuurd. Indien u na het intrekken van de maatregelen alsnog een ondertekend besluit nodig heeft, verneemt de METC dit graag.

c.c. CCMO (pdf via TOL)
c.c. pdf per e-mail A.G.Holleboom, V.A.T.Houttu, N.Sons

AMC en VUmc werken
samen in Amsterdam UMC

AMC
Meibergdreef 9
1105 AZ Amsterdam

Postbus 22660
1100 DD Amsterdam

T +31(0)20 566 9111
www.amc.nl
