# Supplementary material for: Deep phenotyping of patients with MASLD upon high-intensity interval training
Source: JHEP Rep. 2024 Dec 16;7(3):101289. doi: 10.1016/j.jhepr.2024.101289 (PMC11883402; doi:10.1016/j.jhepr.2024.101289)
Supplement: Multimedia component 6 [file mmc6.pdf]

Deep phenotyping of patients with MASLD upon high-intensity interval training

Veera Houttu[1,2,3,4,†], **Ulrika Boulund**[1,2,3,4,†], Marian Troelstra[5], Susanne Csader[6], Daniela Stols-Gonçalves[1,4], Anne Linde Mak[1,4], Anne-Marieke van Dijk[1,4], Julia Bouts[1], Maaike Winkelmeijer[2], Xanthe Verdoes[2], Sandra van den Berg-Faay[5], Donne Lek[7], Ted Ronteltap[7], Ferdinand de Haan[7], Harald Jorstad[8], Ville Männistö[9], Kai Savonen[6,10], Heikki Pentikäinen[10], Kati Hanhineva[6,11,12], Ambrin Farizah Babu[6,11], Gianni Panagiotou[13,14,15,16], Otto van Delden[17], Joanne Verheij[18], Michial Doukas[19], Aart Nederveen[5], Ursula Schwab[6], Aldo Grefhorst[1,2,3,4], Max Nieuwdorp[1,2,3,4], Adriaan Georgius Holleboom[1,2,3,4,*]

JHEP Reports **2025**. vol. 7 | 1–13

Check for updates

Background & Aims: Exercise is a key component of lifestyle management in patients with metabolic dysfunction-associated steatotic liver disease (MASLD), but neither its therapeutic effect on the active stage of the disease, that is metabolic dysfunction-associated steatohepatitis (MASH) nor the mediating mechanisms have been characterized. Therefore, we performed multi-omic phenotyping of patients with MASLD-MASH on an exercise program.

Methods: Fifteen patients with MASLD conducted high-intensity interval training (HIIT) combined with home-based training for 12 weeks. MASLD was evaluated using histology, transient elastography, and multiparametric magnetic resonance imaging (MRI) before and after the intervention. Change in maximal oxygen consumption (VO_{2max}) and MRI-determined liver fat were compared with a control group of patients with MASLD (n = 22). RNA sequencing was performed on liver, muscle, and fat biopsies of patients in the exercise group. Stool was analyzed by shotgun metagenomics and untargeted metabolomics was performed on plasma, urine, adipose, and stool.

Results: HIIT increased VO_{2max} by 10.1% and improved mitochondrial metabolism in skeletal muscle, indicating improved cardiorespiratory fitness and adherence. VO_{2max} increased significantly in the exercise group compared with controls. Histologically, no reduction in steatosis, MASH, or liver fibrosis was observed; however, transient elastography tended to improve. MRI-determined liver fat did not change in the exercise group compared with controls. HIIT induced changes in mRNA expression of genes related to beiging of adipose tissue and fibrogenesis in liver. In addition, specific gut microbial taxa and metabolites changed.

Conclusions: HIIT increased cardiorespiratory fitness and induced beneficial gene expression changes in muscle, adipose tissue, and liver, but without translation into histological improvement of MASLD. Longer exercise intervention trials are warranted to validate or refute current recommendations for exercise as a cornerstone treatment for MASLD-MASH.

Clinical trial registry: Dutch Trial Register (registration number NL7932).

© 2024 The Author(s). Published by Elsevier B.V. on behalf of European Association for the Study of the Liver (EASL). This is an open access article under the CC BY license (http://creativecommons.org/licenses/by/4.0/).

Introduction

In co-occurrence with obesity and type 2 diabetes mellitus (T2DM), metabolic dysfunction-associated steatotic liver disease (MASLD)[1] is now the primary cause of chronic liver disease, affecting 25–33% of the global population, and 55–70% of patients with T2DM.[2,3] The spectrum of MASLD ranges from isolated steatosis to metabolic dysfunction-associated steatohepatitis (MASH) and fibrosis, which can culminate in cirrhosis and hepatocellular carcinoma. Additionally, MASLD doubles the risk of atherosclerotic cardiovascular disease.[4] The fibrotic stages of MASLD are associated with increased liver-related and all-cause mortality.[5]

The pathophysiology of MASLD is multifactorial, occurring in the setting of obesity, insulin resistance, and a sedentary lifestyle[6] with unhealthy dietary factors.[7] Lifestyle modulation remains the cornerstone to prevent and treat MASLD, especially since no approved pharmacotherapy for MASLD has yet arrived.[8] Studies report that exercise might alleviate hepatic steatosis, even in the absence of weight loss, and guidelines therefore recommend it for patients with any MASLD stage.[9] However, the effect of exercise on the active and progressive stages of MASLD, that is MASH and fibrosis, has been poorly characterized.[10] This is mainly because of lack of studies that combine in-depth tissue sampling with multiple methods to

* Corresponding author. Address: A.G. Holleboom, Department of Vascular Medicine, Internal Medicine, Amsterdam UMC, location AMC, Meibergdreef 9, Floor G7, 1105 AZ Amsterdam, The Netherlands. Tel.: +31-20-73-27413.
E-mail address: a.g.holleboom@amsterdamumc.nl (A.G. Holleboom).
† These authors share first authorship.
https://doi.org/10.1016/j.jhepr.2024.101289

assess MASLD, MASH, and fibrosis, such as multiparametric magnetic resonance imaging (MRI), transient electrography, and histological analysis of liver biopsies.

Gut microbiota dysbiosis, small intestinal bacterial overgrowth, reduced gut barrier function and specific microbiome-derived metabolites such as ethanol have all been implicated to affect MASLD-MASH.[11–13] Lifestyle interventions with exercise can modulate the gut microbiome, including gut microbiota associated metabolites.[14,15] However, the metabolic changes occurring in patients with MASLD on the level of the liver, muscle, and subcutaneous adipose tissue upon exercise, in relation to each other and to gut microbes, remain to be discerned.

We performed deep phenotyping of patients with advanced stages of MASLD during an exercise intervention to (a) examine whether exercise *per se*, that is without weight loss, can improve MASLD assessed by imaging and histology and (b) to uncover the potentially mediating metabolic processes involved.

Materials and methods

Study design

We conducted a single-arm 12-week exercise intervention study without altering body weight in patients with histologically characterized MASLD in Amsterdam University Medical Center, Amsterdam, The Netherlands, in compliance with the ethical guidelines of the 1975 Declaration of Helsinki as reflected in *a priori* approval by the appropriate institutional review committee. The study was approved (7/2019) by the Medical Ethics Committee of AUMC, location AMC, and registered in the Dutch Trial Register (registration number NL7932). Each participant gave informed consent in writing before the study. The study process is detailed in the CONSORT flow diagram[16] (Fig. S1) and in Fig. S2.

Patient inclusion

Fifteen adult participants were recruited from the local MASLD clinic. To be eligible, participants had to be aged 18–70 years, have a diagnosis of MASLD (based on liver histology, vibration-controlled transient elastography, conventional ultrasound or MRI) and a BMI <40 kg/m^2. The main exclusion criteria were acute or chronic inflammatory or infectious diseases; excessive alcohol use (>20 units/week for men, >14 units/week for women); diagnosis of other liver diseases (such as hepatitis B, hepatitis C, autoimmune hepatitis, cirrhosis, and hepatocellular carcinoma); cardiorespiratory, neurological or musculoskeletal diseases; type 1 diabetes mellitus; inadequately controlled hypothyroidism, lipodystrophy, depression, or any mental illness rendering the patients unable to understand the purpose and procedures of the study; bleeding disorder or use of anticoagulants; contraindications for MRI scan; participation in any exercise and/or diet program more than twice a week in the 3 months before recruitment, or use of insulin or glucagon-like peptide 1 receptor agonists.

A control group of 22 patients with MASLD was included from an identical exercise intervention study conducted in the University of Eastern Finland, Kuopio, Finland.[15] The 22 patients did not participate in the high-intensity interval training (HIIT) intervention. The design, recruitment, and inclusion criteria have been previously described.[15] Briefly, the patients were recruited from the Kuopio University Hospital (KUH), Kuopio Health Care Centre, and Occupational Health Care. Patients with a diagnosis of MASLD based on ultrasound, MRI or computed tomography, aged 18–70 years and BMI >35 kg/m^2 were eligible. The primary outcome was MRI-determined liver fat.

Exercise intervention

The intervention was a 12-week HIIT program with two monitored sessions per week on a cycle ergometer (Monark ergomedic 839e, Vansbro, Sweden) (details in the Supplementary Materials and Methods). In total, participants performed 3 h of exercise per week including HIIT and home-based low-to moderate-intensity aerobic exercise, in line with international guidelines.[17] HIIT program intensity and efficacy of the exercise intervention were determined by cardiopulmonary exercise testing (Cosmed Quark, Cosmed Omnia 2.0, Rome, Italy) on the cycle ergometer.

Physical activity, and dietary intake monitoring

Compliance to the home-based exercise was monitored using a monitor device and questionnaires. Participants were instructed to maintain their habitual dietary intake during the study period and it was monitored by dietary food diaries. See Supplementary material for details.

MRI of the liver and abdomen

Multiparametric MRI of liver and abdomen of the exercise group were conducted in an overnight fasted state using a clinical 3.0 T MRI unit (Ingenia; Philips, Best, The Netherlands). Liver fat content of the control patients was measured by MRI-PDFF via Siemens Avanto fit, NUMARIS/4 (1.5T); Syngo MR E11 and Siemens Aera, NUMARIS/4 machines at Kuopio University Hospital (KUH).

Biopsies of liver, skeletal muscle, and adipose tissue

Percutaneous ultrasound-guided liver biopsies were performed in the exercise group by an interventional radiologist at the Amsterdam UMC according to standard procedures after an overnight fast. One of the participants declined follow-up liver biopsy. For each biopsy, liver samples were distributed to the clinical pathology laboratory at AMC for histology (see details in the Supplementary material). Ultrasound-guided muscle biopsies of the vastus lateralis were performed in the exercise group using a 14G biopsy needle under local anesthesia (10–20 ml of 20 mg/ml lidocaine) by an interventional radiologist according to local standard procedures after an overnight fast. Percutaneous subcutaneous adipose tissue biopsies were taken from the abdominal region by vacuum-liposuction. All tissue samples were snap-frozen in liquid nitrogen and stored at -80 °C.

Statistical analysis

Clinical outcomes

Clinical outcomes and the outcomes of the cardiopulmonary exercise test were analyzed in R version 3.6.1 (R Foundation for Statistical Computing, Vienna, Austria) and IBM SPSS, version 28 (IBM, Chicago, IL, USA). See details in the Supplementary material.

Bioinformatic analyses

All analyses were performed in R version 4.1.3. FDR adjusted *p* values <0.05 were considered significant. See details in the Supplementary material.

Results

Patient baseline characteristics

Fifteen adult patients with MASLD, of whom seven had MASH, were included in the exercise intervention, and a group of 22 control patients with MASLD without exercise intervention completed the study protocol (see Table 1 for baseline characteristics). Compared with the control group, patients in the exercise group were younger (44.4 ± 13.1 *vs.* 56.7 ± 10.7 years of age, *p* <0.01) and had higher BMI (35.0 ± 4.2 *vs.* 29.5 ± 4.3 kg/m^2, *p* <0.01) and HOMA-IR (23.8 [14.8, 43.7] *vs.* 5.9 [2.7, 7.4], *p* <0.01). Weight-adjusted cardiorespiratory fitness (VO_{2max}, ml/min/kg) at baseline was not different between the groups (24.8 ± 4.9 *vs.* 25.1 ± 5.2 ml/min/kg, *p* = 0.62), nor was MRI-determined liver fat content (19.2 ± 7.0 *vs.* 15.1 ± 11.0, *p* = 0.13).

Table 1. Baseline characteristics of the exercise and the control group.

	Exercise group (n = 15)	Control group (n = 22)	*p* value
Age (years)	44.4 (13.1)	56.7 (10.7)	**<0.01**
Female (n, %)	10 (66.6)	12 (48.0)	0.33
T2DM (n, %)	2 (13.3)	4 (16.0)	1.00
VO_{2max} (L/min)	2.7 (0.8)	2.2 (0.5)	**0.01**
VO_{2max} (ml/min/kg)	24.8 (4.7)	25.1 (5.2)	0.62
Power (W)	218.0 (65.5)	165.6 (57.5)	**0.02**
Weight (kg)	110.7 (20.4)	86.5 (16.0)	**<0.01**
BMI (kg/m^2)	35.0 (4.2)	29.5 (4.3)	**<0.01**
Visceral fat (volume)	263 (108)		
Subcutaneous fat (volume)	375 (149)		
Waist circumference (cm)	112.9 (12.3)	101.6 (12.1)	**0.01**
WHR	0.99 (0.01)		
Fat mass (%)	38.5 (7.8)	30.0 (9.0)	0.10
Lean mass (kg)	64.3 (13.8)	31.8 (6.8)	**<0.01**
REE (kcal/day)	2,058 (456)	1,582 (270)	**<0.01**
Fasting glucose (mmol/L)	5.9 [5.4, 6.3]	6.0 [5.5, 6.6]	0.70
Insulin (mmol/L)	113.9 (67.0)	20.1 (10.4)	**<0.01**
HbA1c (mmol/mol)	44 [36, 46]	38 [35.5, 40.0]	0.11
HOMA-IR	23.8 [14.8, 43.7]	5.9 [2.7, 7.4]	**<0.01**
TC (mmol/L)	5.3 [4.4, 5.9]	4.8 [4.3, 5.4]	0.31
LDL-C (mmol/L)	3.5 [2.4, 2.9]	3.0 [2.7, 3.6]	0.58
HDL-C (mmol/L)	1.13 [1.0, 1.3]	1.3 [1.1, 1.6]	0.06
Triglycerides (mmol/L)	2.1 [1.1, 2.6]	1.4 [1.1, 2.0]	0.60
ALT (IU/L)	59.0 [39.5, 78.0]	49.0 [32.5, 68.5]	0.41
AST (IU/L)	38 [30.0, 65.0]	36.0 [30.5, 43.5]	0.32
γGT (IU/L)	42 [35.5, 76.5]	46.0 [25.5, 46.0]	0.93
**Elastography and MRI**			
CAP (dB/m)	341.5 (45.8)		
LSM (kPa)	9.6 [7.1, 13.4]		
Liver fat (MRI-PDFF, %)	19.2 (7.0)	15.1 (11.0)	0.13
Liver fat (MRS, %)	19.2 (6.6)		
Liver fat (3-point Dixon, %)	15.8 (7.0)		
Liver stiffness (MRE, kPa)	1.8 [1.6, 1.9]		
**Liver histology**			
Steatosis grade (0/1/2/3)	0/5/6/4		
MASLD activity score	3.7 (7.3)		
Lobular inflammation score (0/1/2/3)	1/13/1/0		
Hepatocyte ballooning score (0/1/2)	7/6/2		
Fibrosis stage (0/1/2/3/4)	1/1/9/4/0		
**Dietary intake**			
Energy intake (kcal/day)	1,895 (691)	2,272 (533)	**0.04**
Carbohydrates (g/day)	204.5 (78.5)	236.5 (57.6)	0.08
Carbohydrates (E-%)	43.4 (6.7)	41.8 (4.5)	0.20
Protein (g/day)	81.5 (32.0)	97.5 (28.3)	0.06
Protein (E-%)	17.2 (3.4)	17.1 (2.3)	0.50
Fat (g/day)	75.3 (31.0)	94.6 (26.9)	**0.03**
Fat (E-%)	36.0 (4.7)	37.3 (5.0)	0.14
Fiber (g/day)	18.3 (7.0)	26.4 (9.7)	**0.04**

Data presented for normally distributed variables are means ± standard deviation (SD) (independent *t* test), and for non-normally distributed variables are median with IQR (Mann–Whitney *U* test). Empty column entries indicate that the parameter was not collected. ALT, alanine aminotransferase; AST, aspartate-aminotransferase; BMI, body mass index; CAP, controlled attenuation parameter; dB/m, decibel per meter; E-%, energy percentage; γGT, gamma-glutamyltransferase; HbA1c, hemoglobin A1c; HDL-C, high-density lipoprotein cholesterol; HOMA-IR, homeostatic model for insulin resistance; IU/L, international unit per liter; LSM, liver stiffness measurement; MRE, magnetic resonance elastography; MRI-PDFF, multiparametric MRI; MRS, magnetic resonance spectroscopy; MASLD, non-alcoholic fatty liver disease; n, number of participants; REE, resting energy expenditure; TC, total cholesterol; T2DM, type 2 diabetes mellitus; VO_{2max}, maximal oxygen consumption; W, watts; WHR, waist-to-hip ratio.

Exercise improved cardiorespiratory fitness and affected skeletal muscle gene expression

Fifteen patients with MASLD completed the 12-week HIIT program. Table 2 shows within-group and between-group comparisons in respect to change before and after intervention. In response to the exercise intervention, the maximum oxygen consumption (VO_{2max}) increased significantly from 24.7 ± 4.7 to 27.2 ± 5.6 ml/min/kg (p <0.01) (Fig. 1A), indicating improved cardiorespiratory fitness and underscoring the efficacy of, and good compliance to the intervention. In line, power (218.0 ± 65.5 *vs.* 244.9 ± 72.7 W, p <0.01) increased significantly (Fig. 1B). In the control group, VO_{2max} did not increase (baseline 25.1 ± 5.2 *vs.* end 24.9 ± 4.8 ml/min/kg, p = 0.43), nor did power (baseline 86.5 ± 16.0 *vs.* end 86.6 ± 15.9 W, p = 0.41), rendering significant differences in VO_{2max} and power between the study groups, (p <0.01 for both) (Table 2, Fig. 1A and B). Body weight (as per study design), BMI, and waist-to-hip ratio did not change in the exercise or in the control group. However, in the exercise group MRI-determined visceral fat volume decreased by 5.8% despite unchanged dietary energy intake and macronutrient composition.

In line with improved cardiorespiratory fitness, exercise had strong effects on muscle mRNA expression with an upregulation of 468 and downregulation of 133 genes (Wald test, FDR adjusted p <0.05) (Fig. 1C–E, Table S1). Most upregulated genes relate to mitochondrial electron transport and Krebs cycle, for example mitochondrial encoded cytochrome C oxidase II (*MT-CO2*), malate dehydrogenase 2 (*MDHD2*), and succinate dehydrogenase complex iron sulfur subunit B (*SDHB*). Fibronectin type III domain containing 5 (*FNDC5*) encoding the myokine irisin,[18] was among the strongest upregulated genes, whereas *MSTN* encoding the myokine myostatin was downregulated. Double C2 domain (*DOCB2*), a gene related to skeletal muscle insulin sensitivity,[19] was significantly downregulated. The expression of the gene encoding insulin growth factor binding protein 2 (*IGFBP2*), a factor related to glucose metabolism,[20] was upregulated.

Delta VO_{2max} correlated with changes in muscle mRNA expression of genes such as those encoding capping actin protein of muscle Z-line subunit beta (*CAPZD*), branched-chain keto acid dehydrogenase kinase (*BCKDK*), fumarate hydratase (*FH*), and mitochondrially encoded NADH:Ubiquinone oxidoreductase core subunit 1 (*MT-ND1*) (Table S2).

The exercise program affected adipose tissue gene expression

Exercise in the intervention group resulted in the upregulation of the adipose tissue mRNA expression of 57 genes whereas it reduced mRNA expression of 24 genes (Fig. 2A; Table S1). Among the upregulated genes were those encoding carboxypeptidase A3 (CPA3), which is involved in the beiging of adipose tissue,[21] and C1QTNF3, an adiponectin homologue associated with healthier, less insulin resistant adipose tissue.[22] In line, HOMA-IR decreased in the exercise group and was significantly different between the exercise and control group (Table 2). Biological processes of upregulated genes were related to extracellular matrix organization, angiotensin regulation, and maturation, whereas the downregulated genes were part of amine and ethanol metabolic processes (Fig. 2B and C).

HIIT did not result in histological improvement of MASLD, but did affect fibrosis genes

On average, the 12-week HIIT in the exercise group did not affect histological steatosis (Z = -1.0, p = 0.32), inflammation (Z = -1.4, p = 0.16), hepatocyte ballooning (Z = -0.45, p = 0.66) or fibrosis (Z = 0.45, p = 0.66). In line, NAFLD activity score (NAS) did not change (3.7 ± 7.3 *vs.* 3.8 ± 9.0, p = 0.74). Large interindividual differences in the hepatic response to HIIT were observed (Fig. 3A–D). Steatosis assessed with MRI-proton density fat fraction (PDFF), 3-point Dixon, and magnetic resonance spectroscopy (MRS) also did not improve in the exercise group, or in the control group (Table 2). Interindividual variability in changes on MRI-PDFF were seen in both groups, ranging from 10% reductions to a 10% increase in the exercise group, and from 7% reductions to 6% increase in the control group (Fig. 3E). Although the FibroScan® (Echosens, Paris, France) controlled attenuation parameter for steatosis did not improve, liver stiffness measurement tended to be lower after exercise (Table 1).

HIIT increased the hepatic mRNA expression of 13 genes and decreased the expression of three genes (Fig. 3F and Table S1). Of interest, four of the altered genes relate to liver fibro-inflammation, *SKIL,* encoding SKI like proto-oncogene,[23] *SRSF3,* splicing factor 3b subunit 3, *FN,1* encoding fibronectin 1,[24] and *ARHGAP35,* Rho GTPase activating protein 35.[25] Among the upregulated biological processes that these upregulated genes relate to were L-amino acid import, nuclear factor of activated T-cells (NFAT) protein regulation, and integrin activation (Fig. 3G). Downregulated biological processes were related to cellular response to glucagon, signaling pathway of bone morphogenetic protein (BMP), and regulation of mRNA processing (Fig. 3H).

Highly variable individual fecal microbiota composition

Next, we used metagenomic analysis of stool samples of the exercise group at the baseline, midpoint, and endpoint of the intervention. There was no significant difference in α-diversity (Shannon index, and evenness), richness, and β-diversity (Bray-Curtis) between these time points (Fig. S4, Table S5). However, the abundance of specific microbial species was affected. Specifically, members of the phyla Euryarcheaota and Lentisphaera, the latter previously linked to steatosis grade in MASLD.[26] increased in relative abundance, whereas members of Proteobacteria, Bacteroidetes, Actinobacteria, and Firmicutes exhibited either an increase or decrease in abundance (Fig. 4A). Enterotype analysis revealed a great microbial heterogeneity among the patients already before the intervention (Fig. 4B). The abundance of microbial pathways upon exercise showed a nominally significant change in six pathways, but none passed adjustment for multiple testing (Table S6).

Metabolomics of stool, plasma, urine, and adipose tissue in response to the exercise program

Untargeted metabolomic profiling of plasma, stool, urine, and adipose tissue at baseline and the end in the exercise group revealed significantly altered abundance of five metabolites in urine, two in plasma, one in adipose tissue, and one in stool (Fig. 5A–D, Table S7). Exercise decreased the abundance glycerophospholipids in plasma but increased their abundance

Table 2. Change in clinical outcomes upon the exercise protocol in comparison with the control group.

	Exercise group estimates (n = 15)	Exercise group *p* value	Control group estimates (n = 22)	Control group *p* value	Exercise *vs.* control estimate	Exercise *vs.* control *p* value
VO_{2max} (L/min)	**0.32 (0.08)**	**1.01e-03**	-0.03 (0.03)	4.06e-01	**0.35 (0.08)**	**4.43e-05**
VO_{2max} (mL/min/kg)	**0.44 (0.11)**	**1.15e-03**	-0.04 (0.05)	4.29e-01	**0.49 (0.11)**	**1.00e-04**
Power (W)	**0.38 (0.06)**	**2.86e-05**	-0.03 (0.03)	3.32e-01	**0.41 (0.06)**	**1.59e-07**
Weight (kg)	0.00 (0.03)	9.06e-01	0 .00 (0.02)	8.36e-01	0.00 (0.04)	9.98e-01
BMI (kg/m^2)	0.01 (0.05)	9.06e-01	0.01 (0.03)	8.36e-01	0.00 (0.05)	9.98e-01
Visceral fat (volume)	**-0.14 (0.06)**	**3.76e-02**				
Subcutaneous fat (volume)	0 (0.09)	9.87e-01				
Waist circumference (cm)	0.06 (0.12)	6.09e-01	**0.70 (0.03)**	**1.99e-02**	-0.01 (0.10)	9.60e-01
WHR	0.16 (0.27)	5.46e-01				
Fat mass (%)	**0.12 (0.05)**	**3.81e-02**	0.06 (0.04)	2.20e-01	0.06 (0.07)	3.87e-01
Lean mass (kg)	-0.03 (0.02)	1.84e-01	-0.02 (0.01)	2.51e-01	-0.01 (0.03)	5.74e-01
REE (kcal/day)	0.06 (0.11)	5.74e-01	0.00 (0.05)	9.77e-01	0.06 (0.11)	5.54e-01
Fasting glucose (mmol/L)	0.03 (0.35)	9.23e-01	0.10 (0.05)	1.34e-01	-0.07 (0.30)	8.16e-01
Insulin (mmol/L)	-0.36 (0.22)	1.35e-01	0.06 (0.05)	2.51e-01	**-0.42 (0.19)**	**3.43e-02**
HbA1c (mmol/mol)	-0.07 (0.14)	6.24e-01	0.11 (0.07)	1.22e-01	-0.18 (0.14)	2.14e-01
HOMA-IR	-0.33 (0.23)	1.73e-01	0.08 (0.06)	1.87e-01	**-0.41 (0.20)**	**4.65e-02**
TC (mmol/L)	-0.06 (0.12)	5.90e-01	-0.11 (0.09)	2.24e-01	0.05 (0.14)	7.47e-01
LDL-C (mmol/L)	-0.36 (0.38)	3.62e-01	-0.13 (0.09)	1.47e-01	-0.23 (0.33)	4.95e-01
HDL-C (mmol/L)	0.14 (0.1)	2.02e-01	-0.1 (0.07)	1.90e-01	0.24 (0.12)	6.21e-02
Triglycerides (mmol/L)	0.05 (0.19)	8.11e-01	0.01 (0.1)	9.60e-01	0.04 (0.2)	8.41e-01
ALT (IU/L)	-0.21 (0.15)	1.76e-01	-0.05 (0.11)	6.67e-01	-0.17 (0.18)	3.62e-01
AST (IU/L)	-0.2 (0.18)	2.97e-01	-0.06 (0.14)	6.83e-01	-0.13 (0.23)	5.76e-01
γGT (IU/L)	-0.14 (0.07)	7.17e-02	-0.05 (0.06)	4.59e-01	-0.10 (0.10)	3.33e-01
**Elastography and MRI**	**Exercise group estimates (n = 15)**	**Exercise group *p* value**	**Control group estimates (n = 22)**	**Control group *p* value**	**Exercise *vs.* control estimate**	**Exercise *vs.* Control *p* value**
CAP (dB/m)	0.14 (0.27)	6.17e-01				
LSM (kPa)	-0.22 (0.11)	6.84e-02				
Liver fat (MRI-PDFF, %)	-0.09 (0.15)	5.69e-01	-0.07 (0.08)	4.36e-01	-0.02 (0.16)	8.86e-01
Liver fat (MRS, %)	-0.41 (0.27)	1.44e-01				
Liver fat (3-point Dixon, %)	-0.12 (0.25)	6.30e-01				
Liver stiffness (MRE, kPa)	-0.11 (0.12)	3.54e-01				
**Liver histology**	**Exercise group, before *vs.* after HIIT (n = 15)**	**Within group *p* value**				
MASLD activity score	-0.48 (0.91)	5.99e–01				
Steatosis grade (0/1/2/3)	0/5/6/4 *vs.* 0/6/4/4	0.32				
Lobular inflammation score (0/1/2/3)	1/13/1/0 *vs.* 0/12/2/0	0.16				
Hepatocyte ballooning score (0/1/2)	7/6/2 *vs.* 8/3/3	0.66				
Fibrosis state (0/1/2/3/4)	1/1/9/4/0 *vs.* 0/4/6/4/0	0.66				
**Dietary intake**	**Exercise group estimates (n = 15)**	**Exercise group *p* value**	**Control group estimates (n = 22)**	**Control group *p* value**	**Exercise *vs.* control estimate**	**Exercise *vs.* control *p* value**
Energy intake (kcal/day)	-0.13 (0.15)	3.96e-01	-0.28 (0.15)	7.54e-02	0.15 (0.22)	5.04e-01
Carbohydrates (g/day)	-0.11 (0.16)	4.91e-01	**-0.28 (0.12)**	**2.97e-02**	0.17 (0.2)	3.87e-01
Carbohydrates (E-%)	-0.28 (0.51)	5.86e-01	-0.02 (0.13)	8.95e-01	-0.26 (0.45)	5.66e-01
Protein (g/day)	0.29 (0.34)	4.08e-01	-0.22 (0.15)	1.53e-01	0.51 (0.33)	1.33e-01
Protein (E-%)	0.69 (0.46)	1.47e-01	-0.05 (0.17)	7.94e-01	0.73 (0.45)	1.12e-01
Fat (g/day)	-0.21 (0.18)	2.46e-01	-0.24 (0.19)	2.28e-01	0.03 (0.28)	9.24e-01
Fat (E-%)	-0.3 (0.28)	2.94e-01	-0.01 (0.21)	9.78e-01	-0.3 (0.34)	3.94e-01
Fiber (g/day)	0.17 (0.11)	1.55e-01	-0.19 (0.13)	1.56e-01	0.36 (0.18)	5.64e-02

Group data presented are mean difference with standard errors. For the within-group and between-group comparisons, a linear mixed-effects model was used. For ordinal outcomes a Wilcoxon test was used. Empty column entries indicate that the parameter was not collected. ALT, alanine aminotransferase; AST, aspartate-aminotransferase; BMI, body mass index; CAP, controlled attenuation parameter; dB/m, decibel per meter; E-%, energy percentage; γGT, gamma-glutamyltransferase; HbA1c, hemoglobin A1c; HDL-C, high-density lipoprotein cholesterol; HOMA-IR, homeostatic model for insulin resistance; IU/L, international unit per liter; LSM, liver stiffness measurement; MRE, magnetic resonance elastography; MRI-PDFF, multiparametric MRI; MRS, magnetic resonance spectroscopy; MASLD, non-alcoholic fatty liver disease; n, number of participants; REE, resting energy expenditure; TC, total cholesterol; VO_{2max}, maximal oxygen consumption; W, watts; WHR, waist-to-hip ratio.

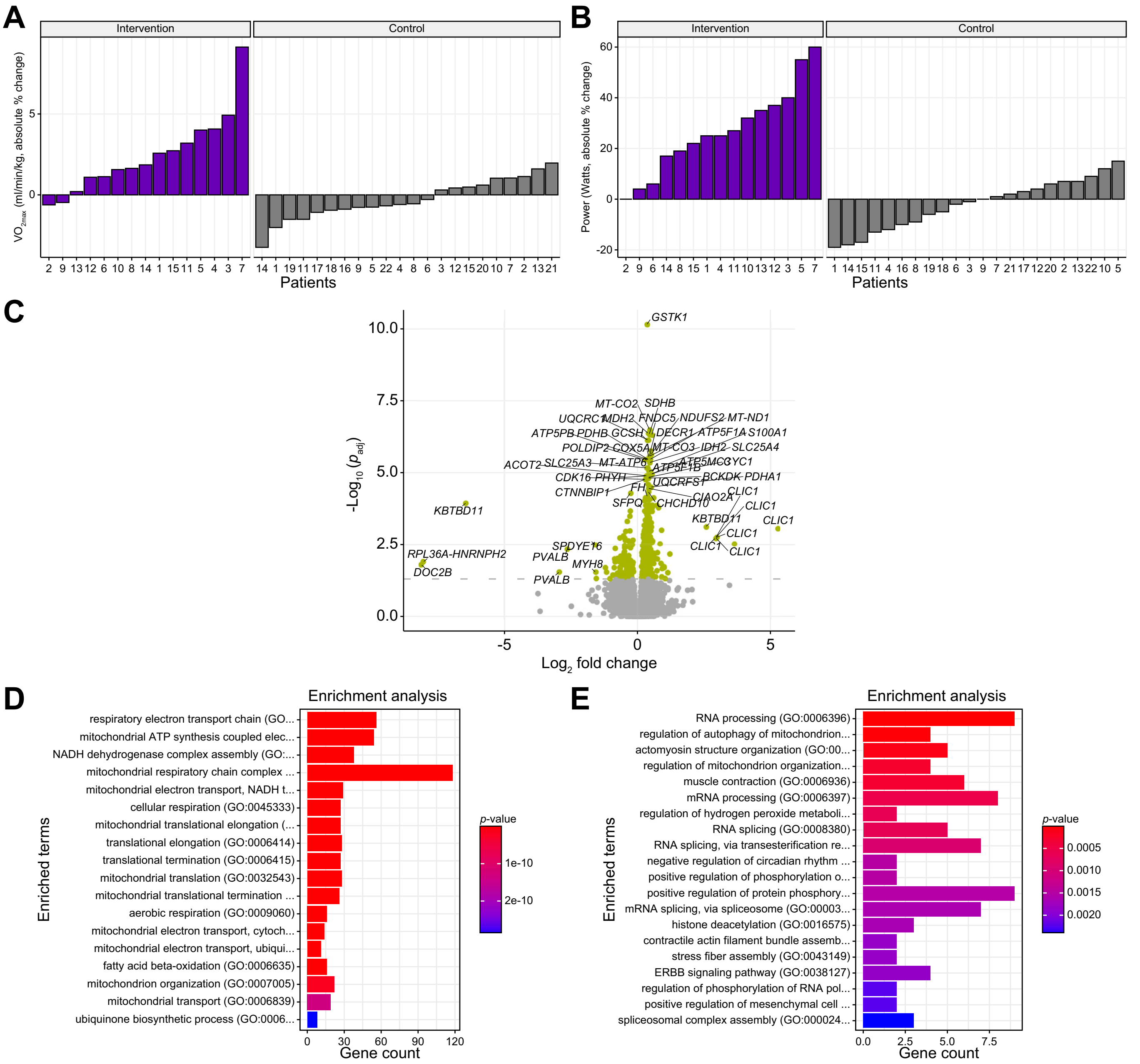

Fig. 1. Cardiorespiratory capacity, and muscle mitochondrial metabolism increase in response to the exercise intervention. (A) Relative individual changes in VO_{2max} (ml/min/kg) upon the exercise program (exercise group n = 15) and intervention (control group n = 22). (B) Relative individual changes in power (W) upon exercise program (n = 15) and intervention (control group n = 22). (C) Volcano plot of changes in skeletal muscle gene expression upon the end of the exercise program (n = 14). Levels of significance: FDR adjusted *p* value <0.05; (Wald test, parametric fit). (D) Enrichment plot of significantly upregulated skeletal muscle genes in Gene Ontology Biological processes (2018). (E) Enrichment plot of the significantly downregulated skeletal muscle genes in Gene Ontology Biological processes (2018). FDR, false discovery rate; VO_{2max}, maximal oxygen consumption.

in adipose tissue (Table S6, Fig. S5A–D). Taurine abundance increased upon exercise.

Crosstalk between liver, muscle, and adipose tissue upon exercise

Inter-relations of exercise-induced changes of clinical outcomes, tissue gene expression, and metabolomic features (Fig. 6, Table S9A and B) revealed that changes in hepatic *SRFS3* mRNA expression correlated positively with changes in adipose tissue *CPA3* mRNA expression in the exercise group. Moreover, changes in muscle *FNDC5* mRNA expression correlated with adipose tissue mRNA expression of 15-hydroxyprostaglandin dehydrogenase (*HPGD*) and histidine decarboxylase (*HDC).* The unknown urinary metabolite Rpneg_2.388@402.998 correlated with muscle mRNA expression of *FNDC5*, *SDHB, MT-CO2*, and glutathione S-transferase (*GST1),* but also with adipose tissue *HDC* mRNA expression. Finally, changes in adipose tissue *HPGD* mRNA expression

correlated with changes in plasma taurine abundance. Additionally, a Procrustes analysis demonstrated that the liver and adipose transcriptome were significantly correlated after the intervention (Table S11, p = 0.003, correlation coefficient = 0.76) and that the adipose and stool metabolome were significantly correlated with each other after the intervention (Table S10, p = 0.038, correlation coefficient = 0.55).

Discussion

This study provides a comprehensive characterization of the effects of a 12-week HIIT intervention without weight loss in patients with MASLD-MASH. The intervention improved cardiorespiratory fitness induced expression of genes involved in beiging of subcutaneous adipose tissue, altered expression of genes involved in liver fibrosis, and tended to lower liver stiffness. However, despite the improved cardiorespiratory fitness and beneficial changes at the gene expression level, liver histology and MRI outcomes did not show improvement of MASLD in this study. The modest reduction in visceral fat in absence of a reduction of MASLD corroborates another study.[27] This may indicate that visceral fat is more sensitive to exercise-induced metabolic improvement than hepatic fat.

Large interindividual differences in the hepatic response to HIIT were observed, as exemplified by the MRI-PDFF data assessing liver steatosis. A responder/non-responder pattern is also commonly observed in pharmacological interventions.[28] Of note, we also observed interindividual differences in MRI-PDFF change in the control group, potentially reflecting the waxing- and waning nature of MASLD.[29] The fact that the changes in VO_{2max} in the exercise group did not translate into an effect on MASLD suggests that increased cardiorespiratory fitness alone is not sufficient to induce a histological effect on MASLD in the timeframe studied.

With respect to the magnitude of cardiorespiratory improvement, it is interesting to compare the patients with MASLD in our study with a recent study in 48 healthy individuals with an average BMI of 23 kg/m^2.[30] This study reports an increase in VO_{2max} of almost 16% upon comparable HIIT exercise intervention, whereas we only observed an increase of 10.1%. Of note, exercise effort was comparable between the studies, with a power increase of 13% in Rodriguez-Garcia *et al.*[30] and 12% in our study. This may raise the hypothesis

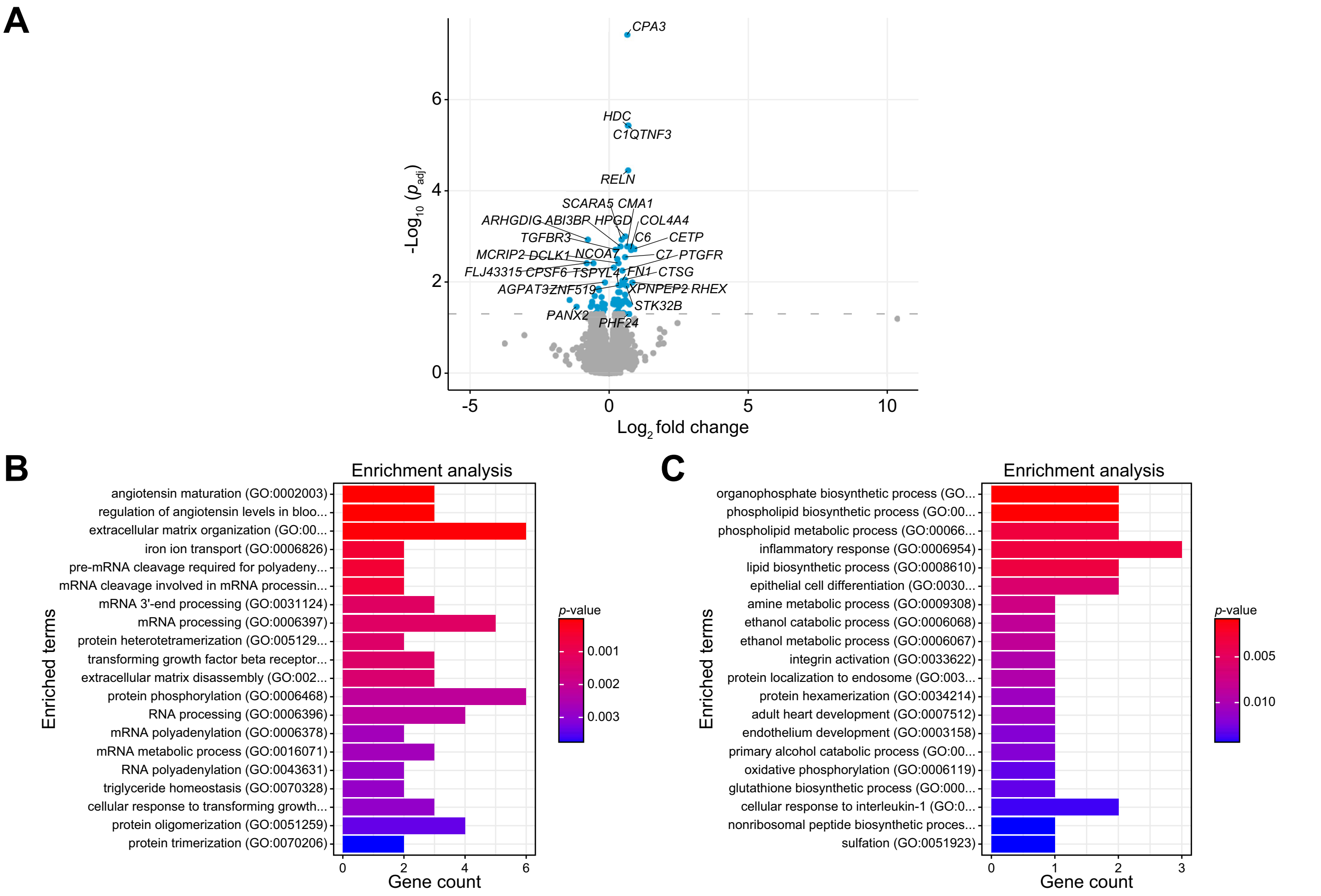

Fig. 2. Adipose tissue transcriptomics in response to the exercise intervention. (A) Volcano plot of changes in adipose tissue gene expression upon the exercise program (n = 15). Levels of significance: FDR adjusted-value p <0.05; (Wald test, parametric fit). (B) Enrichment plot of the significantly upregulated adipose tissue genes in Gene Ontology Biological processes (2018). (C) Enrichment plot of the significantly downregulated adipose tissue genes in Gene Ontology Biological processes (2018). FDR, false discovery rate.

that patients with MASLD and their related cardiometabolic comorbidities may have reduced capability of training effect compared with patients who were non-MASLD, which could bear relevance for clinical practice.

When comparing clinical parameters upon exercise intervention with the multi-omics data, we did observe significant inter-tissue correlations in the patients with MASLD, indicative of modulation of various metabolic tissues by the exercise. The improved cardiorespiratory fitness upon the exercise program was reflected by changes in muscle mRNA expression. Most of the affected genes encode proteins involved in energy metabolism, indicative of an enhanced aerobic capacity of skeletal muscle.[31] More specifically, muscle fat oxidation may have increased relatively more strongly than carbohydrate oxidation, as

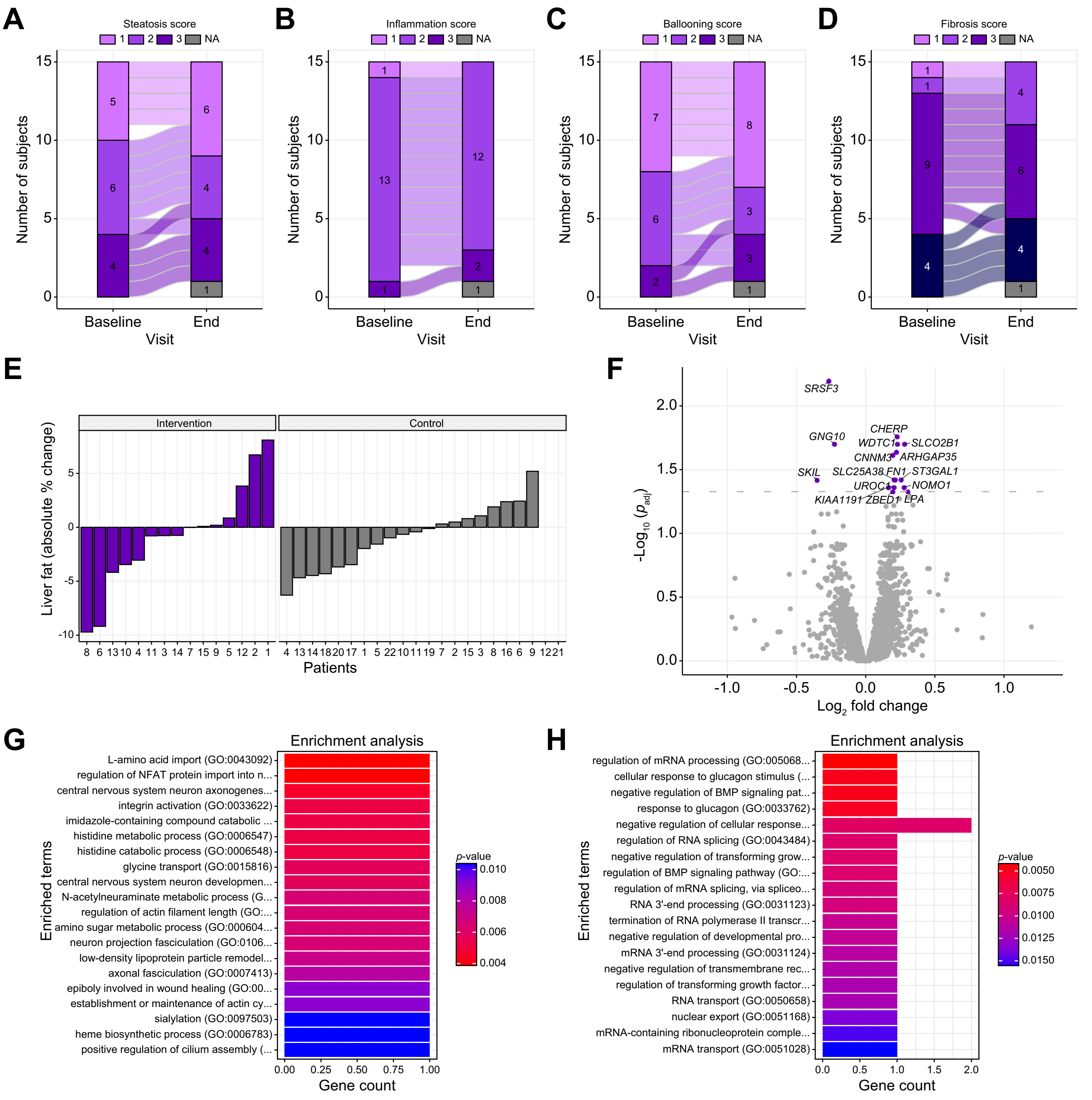

Fig. 3. Exercise has no effect on steatosis, inflammation, ballooning or fibrosis. (A) Steatosis score, (B) inflammation score, (C) ballooning score, and (D) fibrosis score of livers at baseline and the endpoint of the exercise program. (E) Relative individual changes in MRI-PDFF-determined liver fat upon the exercise program (exercise group n = 15) and intervention (control group n = 22). (F) Volcano plot of changes in hepatic gene expression upon the exercise program (n = 13). Levels of significance: FDR adjusted p value <0.05; (Wald test, parametric fit). (G) Enrichment plot of the significantly upregulated liver genes from Gene Ontology Biological processes (2018). (H) Enrichment plot of the significantly downregulated liver genes in Gene Ontology Biological processes (2018). FDR, false discovery rate.

reflected by increased mRNA expression of *ACOT2* encoding acyl-CoA thioesterase 2 that hydrolyzes CoA esters, and of *DECR1* encoding a protein that participates in β-oxidation.[32,33] This might indicate that the HIIT improved the efficiency of muscular energy substrates usage. Exercise also upregulated muscle mRNA expression of *FNDC5* that encodes a precursor of irisin, a myokine secreted in response to exercise.[34] Irisin might promote browning of white adipose tissue and thereby increase adipocyte fatty acid oxidation and lipolysis[35] resulting in increased energy expenditure.[36] We also observed upregulation of *CPA3* in adipose tissue, as a potential sign of browning. Irisin-induced browning of adipose tissue[36] may reduce hepatopetal lipid fluxes and inflammation, important steps in the induction of MASLD.[18] Interestingly, changes in *FNDC5* mRNA expression correlated with changes in the adipose tissue mRNA expression of, for example *HPGD* and *HDC*. Exercise reduced the muscle

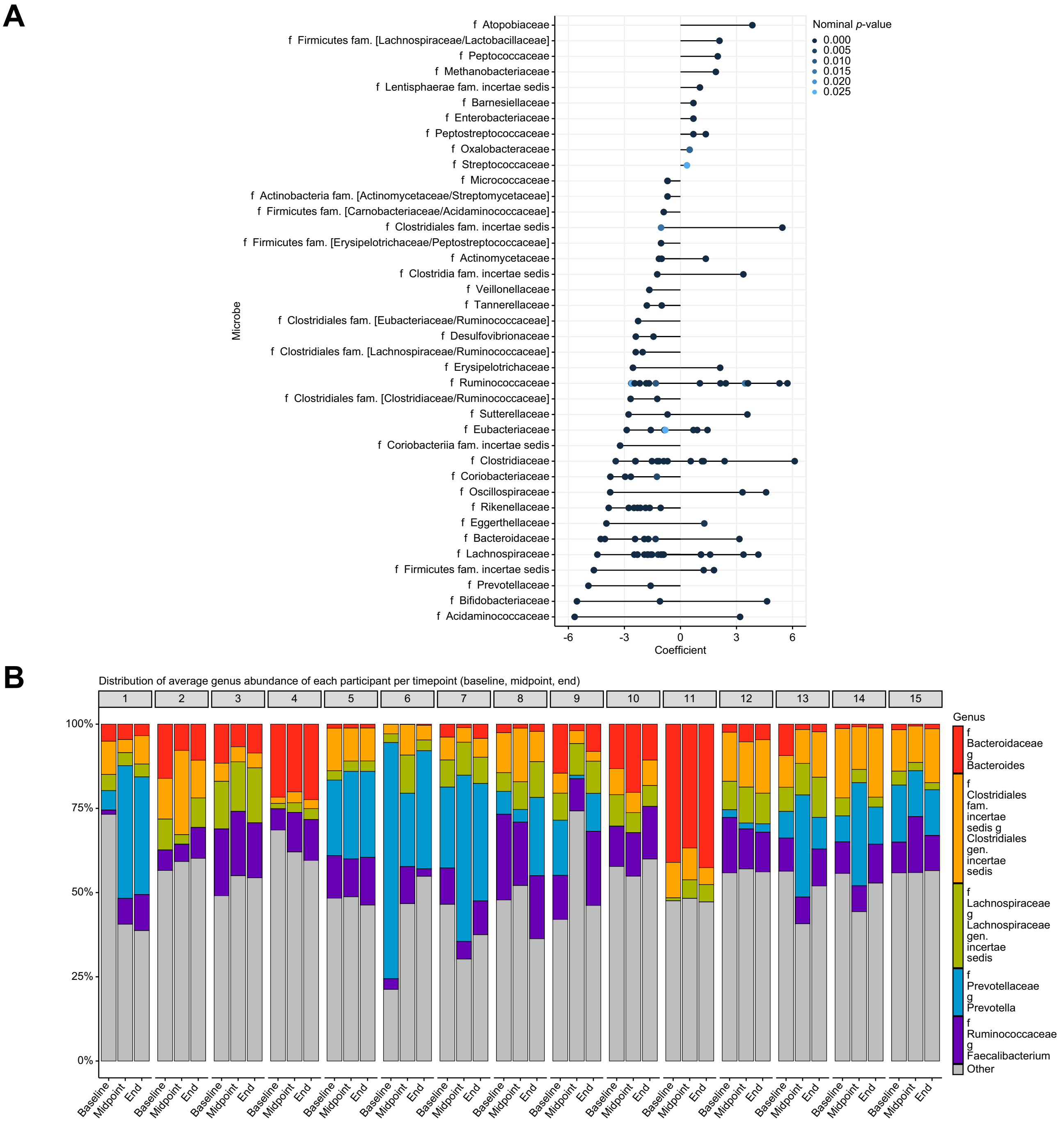

Fig. 4. Gut microbiota composition upon exercise intervention. (A) Linear mixed model regression coefficient of the fecal microbial species in response to the exercise intervention, visualized on family level, the color represents the nominal *p* value >0.05 (n = 15). (B) Enterotypes of patients (n = 15) in different time points of the exercise intervention.

mRNA expression of *MSTN* encoding the myokine myostatin that is associated with muscular atrophy and insulin resistance.[37] This is in line with another study in which exercise decreased plasma myostatin concentrations in MASLD.[38]

Current guidelines endorse exercise for all patients with MASLD, regardless of the severity of MASLD.[9] These guidelines are based on studies in which the steatotic component of MASLD on imaging data. The few exercise studies that do use histological analysis to score MASH and fibrosis show contradictory results. While O'Gorman *et al.*[39] report that HIIT regressed hepatocyte ballooning and fibrosis, Eckhard *et al.*[40] found no histological improvements upon HIIT. These contradictory effects might be attributable to established limitations of using liver biopsies, such as sampling variability.[41] We therefore assessed MASLD non-invasively with multiparametric MRI and transient elastography and found concordant results of MRI with histological outcomes.

The lack of improvement of MASLD assessed by MRI is discordant with a previous exercise study.[10] Our study has comparable intervention and duration as the previous study but was without weight loss and included advanced MASLD stages. Together, this suggests that although our study demonstrates significant improvements of respiratory fitness and a clear change in metabolically relevant genes in muscle and adipose tissue, weight loss,[42] or a longer intervention duration[40] are likely needed to achieve improvement in MASLD.

Most studies exploring the effects of exercise on gut microbiomes are conducted in athletes rather than in patients.[43] We are among the first to report the effect of exercise on gut microbes in MASLD and show that the relative abundance of members of the phyla Euryarcheaota and Lentisphaera increased with exercise.

Our multi-omics analyses showed that the exercise-induced changes in plasma taurine correlated negatively with adipose tissue mRNA expression of *HPGD* encoding a protein involved in hydroxylated fatty acid oxidation.[44] Taurine has been shown to alleviate MASH,[45] and a recent study showed provoked mitochondrial metabolism in the adipose tissue of patients with obesity when exercise was combined with taurine supplementation,[46] underscoring the potential clinical relevance of taurine in correcting metabolic diseases.

Our study has several strengths. Firstly, this study is among the few that investigate the effect of exercise on MASLD with histological endpoints alongside MRI and transient elastography. Hence, our study can serve as a blueprint to design controlled exercise intervention studies in MASLD. Secondly, our study is unique as it focused on the effect of exercise on MASLD, and patients therefore continued their habitual diet to maintain their

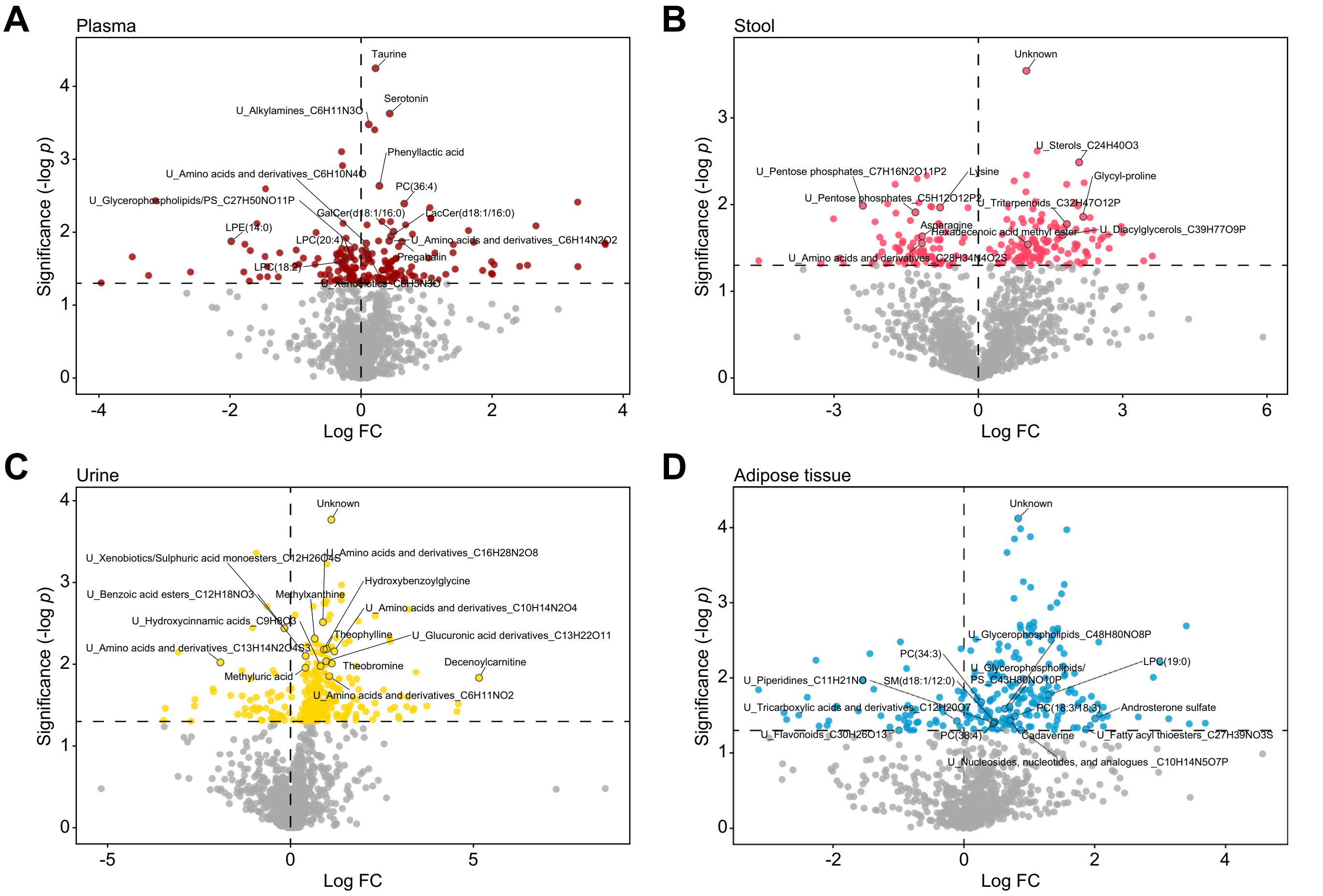

Fig. 5. Effect of the exercise intervention on the metabolome of plasma, stool, urine, and adipose tissue. Volcano plots of results of a linear mixed effect model, the x-axis represents the $\log_2$ fold change of metabolite abundance between baseline and endpoint, the y-axis represents the negative $\log_2$ of the nominal *p* value >0.05. U refers to unknown feature, with known compound class, and formula (A) of plasma metabolome changes upon the intervention (n = 14); (B) stool metabolome (n = 15); (C) urine metabolome (n = 15); (D) adipose tissue metabolome (n = 14).

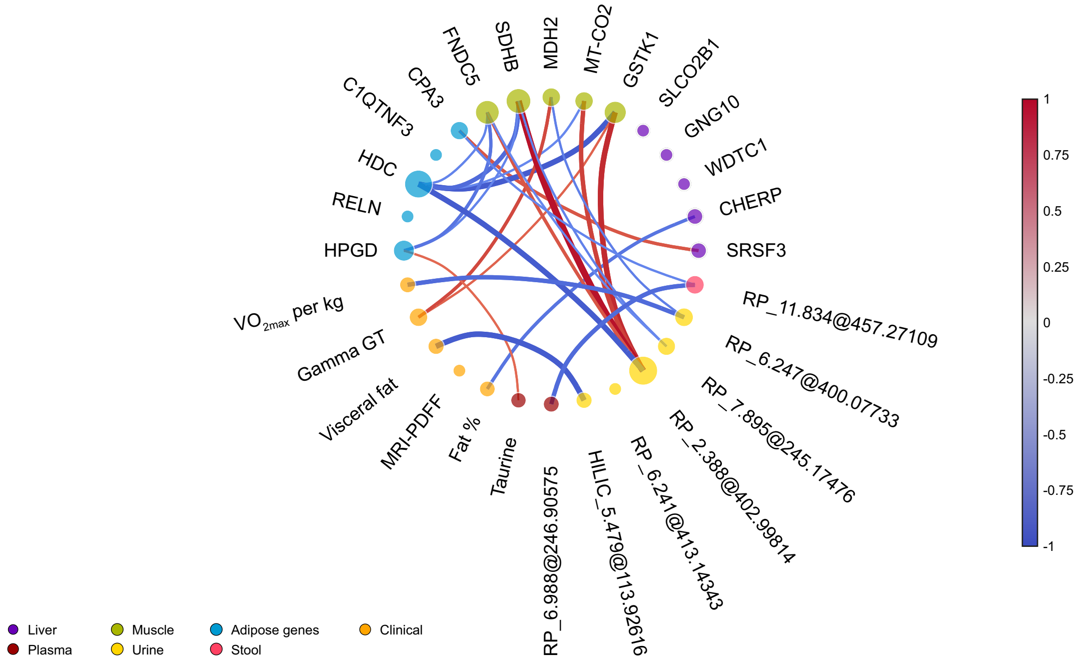

Fig. 6. Associations of relative changes across different tissues and clinical outcomes upon the exercise intervention. The top five of the most significant FDR-corrected variables are included per data type. The significantly altered clinical outcomes, as well as the primary outcome (MRI-determined liver fat, MRI-PDFF) are included. Spearman correlations (Rho >0.6, p <0.05) are illustrated by red (positive) or blue (negative) lines. Circle size reflects the number of correlations, and line size reflects correlation coefficient. For transcriptomics, purple color represents liver genes, green skeletal muscle, blue adipose tissue; for metabolomics red represents plasma, pink stool, yellow urine, and blue adipose tissue; orange color represents clinical outcomes. CPA3, Carboxypeptidase A3; FDR, false discovery rate; FNDC5, Fibronectin type III domain containing 5; GSTK1, glutathione S-transferase kappa 1; HDC, Histidine decarboxylase; HPGD, 15-hydroxyprostaglandin dehydrogenase; MDH2, malate dehydrogenase 2; MRI, magnetic resonance imaging; MT-CO2, Mitochondrial encoded cytochrome C oxidase II; PDFF, proton density fat fraction; SDHB, Succinate dehydrogenase complex iron sulfur subunit B; SRSF3, Splicing factor 3b subunit 3; VO_{2max}, maximal oxygen consumption.

body weight. This exemplifies that the present results are a result of the direct effect of exercise rather than calorie restriction and/or weight loss. Finally, we conducted the most deeply phenotyped study of exercise in MASLD to date using multiple omics layers across several biologically relevant compartments, raising new mechanistic hypotheses that warrant further research, for example the potential rapid response to exercise of fibrogenic gene transcription. These strengths counterbalance the main limitation of our study, the modest sample size owing to COVID-19 pandemic restrictions on inclusions.

In conclusion, in patients with MASLD, an exercise intervention without effect on body weight improved cardiorespiratory fitness, however to a lesser degree than reported for those without liver disease,[30] and did not ameliorate MASLD. This may indicate that MASLD is best treated with combined lifestyle interventions resulting in weight loss, potentially even combined with weight loss medication as published recently.[47] This most deeply characterized multi-omics HIIT intervention uniquely unravels the crosstalk between multiple metabolic tissues and contributes greatly to the biological understanding of the mechanistic effects of exercise in MASLD. Further exercise interventions in severe stages of MASLD are needed to validate guideline recommendations for exercise as a cornerstone treatment.

Affiliations

[1]Department of Vascular Medicine, Amsterdam UMC, University of Amsterdam, Amsterdam, The Netherlands; [2]Experimental Vascular Medicine, Amsterdam UMC, University of Amsterdam, Amsterdam, The Netherlands; [3]Amsterdam Cardiovascular Sciences Institute, Amsterdam UMC, University of Amsterdam, Amsterdam, The Netherlands; [4]Amsterdam Gastroenterology Endocrinology Metabolism Institute, Amsterdam UMC, University of Amsterdam, Amsterdam, The Netherlands; [5]Department of Radiology, and Nuclear Medicine, Amsterdam UMC, University of Amsterdam, Amsterdam, The Netherlands; [6]School of Medicine, Institute of Public Health, and Clinical Nutrition, University of Eastern Finland, Kuopio, Finland; [7]Polifysiek, Amsterdam University of Applied Science, Amsterdam, The Netherlands; [8]Department of Cardiology, Amsterdam Movement Sciences, Amsterdam Cardiovascular Sciences, Amsterdam UMC, University of Amsterdam, Amsterdam, The Netherlands; [9]Department of Medicine, University of Eastern Finland, and Kuopio University Hospital, Kuopio, Finland; [10]Kuopio Research Institute of Exercise Medicine, Kuopio, Finland; [11]Afekta Technologies Ltd., Kuopio, Finland; [12]Department of Life Technologies, Food Chemistry, and Food Development Unit, University of Turku, Turku, Finland; [13]Department of Microbiome Dynamics, Leibniz Institute for Natural Product Research, and Infection Biology, Hans Knöll Institute (HKI), Jena, Germany; [14]Faculty of Medicine, The University of Hong Kong, Hong Kong, China; [15]Faculty of Biological Sciences, Friedrich Schiller University, Jena, Germany; [16]Jena University Hospital, Friedrich Schiller University, Jena, Germany; [17]Department of Interventional Radiology, Amsterdam UMC, Amsterdam, The Netherlands; [18]Department of Pathology, Amsterdam UMC, Amsterdam, The Netherlands; [19]Department of Pathology, Erasmus MC Cancer Institute, University Medical Center Rotterdam, Rotterdam, The Netherlands

Abbreviations

ARHGAP35, ARHGAP35 rho GTPase activating protein 35; BCKDK, Branched-chain keto acid dehydrogenase kinase; BNP, Bone morphogenetic protein; CAPZD, Capping actin protein of muscle Z-line subunit beta; CPA3, Carboxy-peptidase A3; DOCB2, Double C2 domain; FH, Fumarate hydratase; FNDC5, Fibronectin type III domain containing 5; FN1, Fibronectin 1; GST1, Glutathione S-transferase; HDC, Histidine decarboxylase; HIIT, High intensity interval training; HPGD, 15-hydroxyprostaglandin dehydrogenase; IGFBP2, Insulin growth factor binding protein 2; MASH, Metabolic dysfunction-associated steatohepatitis; MASLD, Metabolic dysfunction-associated steatotic liver disease; MDHD2, malate dehydrogenase 2; MRI, magnetic resonance imaging; MRE, magnetic resonance elastography; MRS, Magnetic resonance spectroscopy; MT-CO2, Mitochondrial encoded cytochrome C oxidase II; MT-ND1, Mitochondrially encoded NADH:Ubiquinone oxidoreductase core subunit 1; MSTN, Myostatin; NFAT, Nuclear factor of activated T-cells; PDFF, Proton density fat fraction; SDHB, Succinate dehydrogenase complex iron sulfur subunit B; SKIL, SKI like proto oncogene; SRSF3, Splicing factor 3b subunit 3; VO_{2max}, Maximal oxygen consumption.

Financial support

This project has received funding from the European Union's Horizon 2020 research, and innovation program under the Marie Skłodowska-Curie grant agreement No 813781. MN is supported by a personal ZONMW-VICI grant 2020 (09150182010020) and an ERC-Advanced grant 2023 (101141346). AGH is supported by the Amsterdam UMC Fellowship grant, the Amsterdam UMC Innovation grant, the Dutch Gastroenterology Foundation MLDS, Holland∼Health TKI-PPP and Horizon Europe GRIP on MASH. KH is supported by ERA-Net NEURON (grant no 334814), and Academy of Finland (grant no 321716).

Conflicts of interest

MN is scientific advisor of Caelus Health, however this is not relevant for the content of the current paper.

Please refer to the accompanying ICMJE disclosure forms for further details.

Authors' contributions

Study concept and design: VH, UB, SC, VM, GP, US, AG, MN, AGH. Acquisition of data: VH, SC, DS-G, JB, DL, TR, FDH, US. Statistical analysis: VH, UB. Computational analysis: UB. Interpretation of data: VH, UB, AG, AGH. Technical and material support: MT, DS-G, ALM, A-MD, MW, XV, SB-F, DL, TR, HJ, KS, HP, OD, AN, MN. Technical and data analytical support on metabolomics analysis: KH, AFB, AGH. Imaging data analysis: MT, SB-F, AN. Liver histology analysis: JV, MD. Administrative support: MN, AGH. Study supervision MN, AGH. Obtained funding: GP, MN. Drafting of the manuscript: VH. Contributed to the intellectual content of the manuscript, edited, and approved the final draft of the manuscript: all authors.

Data availability statement

Data are available upon request.

Acknowledgements

We thank all participants for their contribution to the study and Atte Lihtamo for his help with metabolomics pre-processing and statistical analyses. Created in BioRender. Houttu, V. (2024) https://BioRender.com/v72q218.

Supplementary data

Supplementary data to this article can be found online at https://doi.org/10.1016/j.jhepr.2024.101289.

References

Author names in bold designate shared co-first authorship.

[1] Rinella ME, Lazarus JV, Ratziu V, et al. A multi-society Delphi consensus statement on new fatty liver disease nomenclature. J Hepatol 2023;79:1542–1556.

[2] Younossi Z, Tacke F, Arrese M, et al. Global perspectives on nonalcoholic fatty liver disease and nonalcoholic steatohepatitis. Hepatology 2019;69:2672–2682.

[3] Stefan N, Cusi K. A global view of the interplay between non-alcoholic fatty liver disease and diabetes. Lancet Diabetes Endocrinol 2022;10:284–296.

[4] Mantovani A, Csermely A, Petracca G, et al. Non-alcoholic fatty liver disease and risk of fatal and non-fatal cardiovascular events: an updated systematic review and meta-analysis. Lancet Gastroenterol Hepatol 2021;6:903–913.

[5] **Taylor RS**, **Taylor RJ**, Bayliss S, et al. Association between fibrosis stage and outcomes of patients with nonalcoholic fatty liver disease: a systematic review and meta-analysis. Gastroenterology 2020;158:1611. 25.e12.

[6] Kim D, Vazquez-Montesino LM, Li AA, et al. Inadequate physical activity and sedentary behavior are independent predictors of nonalcoholic fatty liver disease. Hepatology 2020;72:1556–1568.

[7] Ivancovsky-Wajcman D, Fliss-Isakov N, Webb M, et al. Ultra-processed food is associated with features of metabolic syndrome and non-alcoholic fatty liver disease. Liver Int 2021;41:2635–2645.

[8] Dufour JF, Anstee QM, Bugianesi E, et al. Current therapies and new developments in NASH. Gut 2022;71:2123–2134.

[9] European Association for the Study of the Liver (EASL), European Association for the Study of Diabetes (EASD), European Association for the Study of Obesity (EASO). EASL-EASD-EASO Clinical Practice Guidelines for the management of non-alcoholic fatty liver disease. J Hepatol 2016;64:1388–1402.

[10] **Houttu V**, **Bouts J**, Vali Y, et al. Does aerobic exercise reduce NASH and liver fibrosis in patients with non-alcoholic fatty liver disease? A systematic literature review and meta-analysis. Front Endocrinol (Lausanne) 2022;13:1032164.

[11] Meijnikman AS, Davids M, Herrema H, et al. Microbiome-derived ethanol in nonalcoholic fatty liver disease. Nat Med 2022;28:2100–2106.

[12] Loomba R, Seguritan V, Li W, et al. Gut microbiome-based metagenomic signature for non-invasive detection of advanced fibrosis in human nonalcoholic fatty liver disease. Cel Metab 2017;25:1054. 62.e5.

[13] Shi H, Mao L, Wang L, et al. Small intestinal bacterial overgrowth and orocecal transit time in patients of nonalcoholic fatty liver disease. Eur J Gastroenterol Hepatol 2021;33(1S Suppl 1):e535–e539.

[14] Cheng R, Wang L, Le S, et al. A randomized controlled trial for response of microbiome network to exercise and diet intervention in patients with nonalcoholic fatty liver disease. Nat Commun 2022;13:2555.

[15] **Babu AF**, **Csader S**, Männistö V, et al. Effects of exercise on NAFLD using non-targeted metabolomics in adipose tissue, plasma, urine, and stool. Sci Rep 2022;12:6485.

[16] Eldridge SM, Chan CL, Campbell MJ, et al. CONSORT 2010 statement: extension to randomised pilot and feasibility trials. BMJ 2016;355:i5239.

[17] US Department of Health and Human Services. Physical activity guidelines for Americans. 2nd ed. Washington, DC: USDHHS; 2018.

[18] Zhu W, Sahar NE, Javaid HMA, et al. Exercise-induced irisin decreases inflammation and improves NAFLD by competitive binding with MD2. Cells 2021;10:3306.

[19] Ramalingam L, Oh E, Thurmond DC. Doc2b enrichment enhances glucose homeostasis in mice via potentiation of insulin secretion and peripheral insulin sensitivity. Diabetologia 2014;57:1476–1484.

[20] Haywood NJ, Slater TA, Matthews CJ, et al. The insulin like growth factor and binding protein family: novel therapeutic targets in obesity & diabetes. Mol Metab 2019;19:86–96.

[21] Finlin BS, Zhu B, Confides AL, et al. Mast cells promote seasonal white adipose beiging in humans. Diabetes 2017;66:1237–1246.

[22] **Micallef P**, **Vujičić M**, Wu Y, et al. C1QTNF3 is upregulated during subcutaneous adipose tissue remodeling and stimulates macrophage chemotaxis and M1-like polarization. Front Immunol 2022;13:914956.

[23] Tecalco-Cruz AC, Sosa-Garrocho M, Vázquez-Victorio G, et al. Transforming growth factor-β/SMAD target gene SKIL is negatively regulated by the transcriptional cofactor complex SNON-SMAD4. J Biol Chem 2012;287:26764–26776.

[24] Sen S, Jumaa H, Webster NJG. Splicing factor SRSF3 is crucial for hepatocyte differentiation and metabolic function. Nat Commun 2013;4:1336.

[25] Salloum S, Jeyarajan AJ, Kruger AJ, et al. Fatty acids activate the transcriptional coactivator YAP1 to promote liver fibrosis via p38 mitogen-activated protein kinase. Cell Mol Gastroenterol Hepatol 2021;12:1297–1310.

[26] Zeybel M, Arif M, Li X, Altay O, et al. Multiomics analysis reveals the impact of microbiota on host metabolism in hepatic steatosis. Adv Sci 2022;9:2104373.

[27] Slentz CA, Bateman LA, Willis LH, et al. Effects of aerobic vs. resistance training on visceral and liver fat stores, liver enzymes, and insulin resistance by HOMA in overweight adults from STRRIDE AT/RT. Am J Physiol Endocrinol Metab 2011;301:E1033–E1039.

[28] Newsome PN, Buchholtz K, Cusi K, et al. A placebo-controlled trial of subcutaneous semaglutide in nonalcoholic steatohepatitis. New Engl J Med 2021;384:1113–1124.

[29] Kleiner DE, Brunt EM, Wilson LA, et al. Association of histologic disease activity with progression of nonalcoholic fatty liver disease. JAMA Netw Open 2019;2:e1912565. 4.
[30] Rodríguez-García L, Ceylan HI, Silva RM, et al. Effects of 10-week online moderate- to high-intensity interval training on body composition, and aerobic and anaerobic performance during the COVID-19 lockdown. Healthcare 2023;12:37.
[31] Perry CGR, Hawley JA. Molecular basis of exercise-induced skeletal muscle mitochondrial biogenesis: historical advances, current knowledge, and future challenges. Cold Spring Harb Perspect Med 2018;8:a029686.
[32] **Hua T**, **Wu D**, Ding W, et al. Studies of human 2,4-dienoyl CoA reductase shed new light on peroxisomal β-oxidation of unsaturated fatty acids. J Biol Chem 2012;287:28956–28965.
[33] Alphey MS, Yu W, Byres E, et al. Structure and reactivity of human mitochondrial 2,4-dienoyl-CoA reductase. J Biol Chem 2005;280:3068–3077.
[34] Miyamoto-Mikami E, Sato K, Kurihara T, et al. Endurance training-induced increase in circulating irisin levels is associated with reduction of abdominal visceral fat in middle-aged and older adults. PLoS One 2015;10:e0120354.
[35] Feraco A, Gorini S, Armani A, et al. Exploring the role of skeletal muscle in insulin resistance: lessons from cultured cells to animal models. Int J Mol Sci 2021;22:9327.
[36] Boström P, Wu J, Jedrychowski MP, et al. A PGC1-α-dependent myokine that drives brown-fat-like development of white fat and thermogenesis. Nature 2012;481:463–468.
[37] Hittel DS, Axelson M, Sarna N, et al. Myostatin decreases with aerobic exercise and associates with insulin resistance. Med Sci Sports Exerc 2010;42:2023–2029.
[38] Oh S, Tsujimoto T, Kim B, et al. Weight-loss-independent benefits of exercise on liver steatosis and stiffness in Japanese men with NAFLD. JHEP Rep 2021;3:100253.
[39] O'Gorman P, Naimimohasses S, Monaghan A, et al. Improvement in histological endpoints of MAFLD following a 12-week aerobic exercise intervention. Aliment Pharmacol Therapeut 2020;52:1387–1398.
[40] Eckard C, Cole R, Lockwood J, et al. Prospective histopathologic evaluation of lifestyle modification in nonalcoholic fatty liver disease: a randomized trial. Therapeut Adv Gastroenterol 2013;6:249–259.
[41] Ratziu V, Charlotte F, Heurtier A, et al. Sampling variability of liver biopsy in nonalcoholic fatty liver disease. Gastroenterology 2005;128 :1898–1906.
[42] Ezpeleta M, Gabel K, Cienfuegos S, et al. Effect of alternate day fasting combined with aerobic exercise on non-alcoholic fatty liver disease: a randomized controlled trial. Cell Metab 2022;35:56–70.e3.
[43] **Houttu V**, **Boulund U**, Grefhorst A, et al. The role of the gut microbiome and exercise in non-alcoholic fatty liver disease. Therapeut Adv Gastroenterol 2020;13:1756284820941745.
[44] Wendell SG, Golin-Bisello F, Wenzel S, et al. 15-Hydroxyprostaglandin dehydrogenase generation of electrophilic lipid signaling mediators from hydroxy ω-3 fatty acids. J Biol Chem 2015;290:5868–5880.
[45] Murakami S, Ono A, Kawasaki A, et al. Taurine attenuates the development of hepatic steatosis through the inhibition of oxidative stress in a model of nonalcoholic fatty liver disease in vivo and in vitro. Amino Acids 2018;50:1279–1288.
[46] De Carvalho FG, Brandao CFC, Batitucci G, et al. Taurine supplementation associated with exercise increases mitochondrial activity and fatty acid oxidation gene expression in the subcutaneous white adipose tissue of obese women. Clin Nutr 2021;40:2180–2187.
[47] Verrastro O, Panunzi S, Castagneto-Gissey L, et al. Bariatric–metabolic surgery versus lifestyle intervention plus best medical care in non-alcoholic steatohepatitis (BRAVES): a multicentre, open-label, randomised trial. Lancet 2023;401:1786–1797.

Keywords: Multi-omic; Gut microbiota; Metabolome; Transcriptomics; Fibrosis; Steatohepatitis; Steatosis; Histology.

Received 26 February 2024; received in revised form 17 November 2024; accepted 22 November 2024; Available online 16 December 2024

Journal of Hepatology, Volume *7*

Supplemental information

Deep phenotyping of patients with MASLD upon high-intensity interval training

Veera Houttu, Ulrika Boulund, Marian Troelstra, Susanne Csader, Daniela Stols-Gonçalves, Anne Linde Mak, Anne-Marieke van Dijk, Julia Bouts, Maaike Winkelmeijer, Xanthe Verdoes, Sandra van den Berg-Faay, Donne Lek, Ted Ronteltap, Ferdinand de Haan, Harald Jorstad, Ville Männistö, Kai Savonen, Heikki Pentikäinen, Kati Hanhineva, Ambrin Farizah Babu, Gianni Panagiotou, Otto van Delden, Joanne Verheij, Michial Doukas, Aart Nederveen, Ursula Schwab, Aldo Grefhorst, Max Nieuwdorp, and Adriaan Georgius Holleboom

Deep phenotyping of patients with MASLD upon high-intensity interval training

Veera Houttu, Ulrika Boulund, Marian Troelstra, Susanne Csader, Daniela Stols-Gonçalves, Anne Linde Mak, Anne-Marieke van Dijk, Julia Bouts, Maaike Winkelmeijer, Xanthe Verdoes, Sandra van den Berg-Faay, Donne Lek, Ted Ronteltap, Ferdinand de Haan, Harald Jorstad, Ville Männistö, Kai Savonen, Heikki Pentikäinen, Kati Hanhineva, Ambrin Farizah Babu, Gianni Panagiotou, Otto van Delden, Joanne Verheij, Michial Doukas, Aart Nederveen, Ursula Schwab, Aldo Grefhorst, Max Nieuwdorp, Adriaan Georgius Holleboom

Table of contents

Supplementary methods 2

Supplementary statistical analysis 7

Supplementary figures 11

Supplementary table legends 16

Supplementary references 17

Supplementary methods

High-intensity interval training program

Each HIIT session involved 10-minute warming-up (at 30 % of intensity), repeated bouts of 2–4 minutes high intensity intervals (85% of intensity) interspersed by three minutes of active recovery intervals (10 % of intensity), and followed by 5-minute cooling-down (at 20 % of intensity) where the intensities were based on the baseline CPET. Intensity was determined as Wmax4 referring to workload that is sustainable for four minutes(1). The session was repeated twice per week on non-consecutive days for 12 weeks during which each high intensity interval of each session was increased by two seconds. At the end of the intervention period, sessions were approximately 50-minute long with 4-minute high intervals. Sessions were conducted in a group of 1-3 participants closely supervised by the researchers and/or the exercise physiologist (V.H., J.B., D.L.) at the Polifysiek, Hogeschool van Amsterdam, Amsterdam, the Netherlands.

Physical activity, and dietary intake monitoring

An objective physical activity monitoring device (Polar Active, Kempele, Finland) was used to monitor the compliance. Leisure time physical activity was assessed by the modified Minnesota Leisure-Time Physical Activity Questionnaire (2,3). This was monitored on 4–5 consecutive days (at least 1 weekend day) at the baseline, midway and at the end of the intervention using online dietary intake lists (exercise group: dietary record application https://mijn.voedingscentrum.nl/nl/eetmeter/; control group: 4-day food diaries from nutrient intakes were calculated using AivoDiet sofware (version 2.2.0.0, Aivo Finland Oy, Turku, Finland) at the baseline and week 11). Both dietary intake and physical activity diaries were checked and assessed at the time of the collection by the researchers and a clinical nutritionist.

Magnetic resonance imaging of the liver and abdomen

Magnetic resonance spectroscopy (MRS), magnitude-based MRI (MRI-M) proton density fat fraction (PDFF), and three-point Dixon were performed to assess liver fat content. The images of MRS, MRI-PDFF, and three-point Dixon were analyzed in a blinded fashion by a single analyst. Magnetic resonance elastography for liver fibro-inflammation and volumes of visceral, and subcutaneous abdominal fat were quantified blinded by a single analyst. Liver fat content of the control patients analysis was performed blinded at Amsterdam UMC as described (4).

Vibration controlled transient elastography

Vibration controlled transient elastography (VCTE, FibroScan) was performed in the exercise group under fasted conditions using a FibroScan® 530 Compact (Echosens, France) with either M- or XL-probe per protocol to assess steatosis with controlled attenuation parameter and liver fibrosis with liver stiffness.

Liver histology

Hematoxylin & eosin and Sirius Red staining of the liver slices were performed according to standard protocols after which they were scored blinded by two pathologists in tandem. The NASH Clinical Research Network was applied to score fibrosis stages (5). The steatosis, activity, and fibrosis score was used to score steatosis; lobular inflammation; and hepatocellular ballooning (6). This score classified the cases into MASH if ballooning with lobular inflammation was present, and when scored fibrosis stage ≥ F2, cases were classified fibrotic MASH.

Body composition, and resting energy expenditure

Waist circumference was determined at the midpoint between the lateral iliac and the lowest rib. Hip circumference was determined at the widest position of the buttocks. Calf circumference was determined at the widest position of the calf when knee kept in 90°. Body composition was determined by bioelectrical impedance analysis (exercise group: Tanita DC-430 MA, Tokyo, Japan; control group: Inbody 720 body composition analyzer, Inbody, USA) in standing position. Resting energy expenditure via measuring oxygen consumption and carbon dioxide production was determined by indirect calorimetry using a computerized flow-through canopy gas-analyzer system (exercise group: Vmax Encore 29; SensorMedics, Anaheim, CA, USA; control group: Cosmed Quark, RMR, Italy).

Biochemistry

Plasma was isolated from overnight fasted blood samples and stored at –80 until biomarker concentrations were measured with routine clinical analytical biochemistry.

Urine samples

Participants collected twenty-four-hour urine using a plastic 3L container. Participants were asked to keep the container refrigerated during the collection period. Samples from the container were stored at –80°C.

Fecal sample collection

Participants collected morning fecal samples using collection tubes with screw-in spoon lid at baseline, mid-, and endpoint, and were stored at –80 °C. If morning fecal sampling was not possible, participants provided a sample from the previous evening.

Tissue RNA extraction, and sequencing

The RNA extraction of liver, muscle, and adipose tissue was done using an RNA isolation protocol optimized for small tissue biopsies. Samples were mixed with 300 µl of TriPure Isolation Reagent (Roche, Basel, Switzerland) and homogenized on ice using a sterile RNA free pestle. After 60 µl of chloroform was added, the samples were placed in Heavy Phase Lock gel tubes (Quanta Bio, Beverly, USA) and centrifuged for 15 minutes, at 4˚C, 12000xg. The aqueous phase was mixed with 70% ethanol and placed on RNeasy MinElute spin columns (QIAgen, Tegelen, the Netherlands). RNA was washed according to the protocol of the manufacturer and eluted in 14 µl of RNAse free water. The concentration of RNA was determined using Biotek Synergy H1 (Agilent Tech, Santa Clara, USA). One liver sample had low RNA yield and was therefore discarded (in total 28 liver samples were further processed). Furthermore, the RIN scores were obtained using TapeStation (Agilent Tech, Santa Clara, USA). T-oligo attached magnetic beads were used to purify messenger RNA from total RNA. The mRNA was fragmented and cDNA was synthesized using random hexamer primers. Thereafter end repair, A-tailing, adapter ligation, size selection amplification and purification was performed. The RNA sequencing of 28 liver samples was conducted using HiSeq 4000 (Illumina) with 150-bp paired-end reads, and 7.5 G raw data per sample at Novogene Co., Ltd. Sequence data has been deposited at the European Genome-phenome Archive under accession number EGAS00001006991.

Fecal DNA extraction and sequencing

Fecal DNA was extracted from 45 fecal samples using a modification of a previously described protocol(7). In short, 250 mg fecal material was lysed using repeated bead beating in STAR buffer (Roche Diagnostics). Total genomic DNA was isolated from the lysates using a Maxwell device (RSC Blood Kit, Qiagen) and DNA was eluted in ~60ul nuclease free water. The library preparation and sequencing for shotgun metagenomics were performed using a HiSeq Illumina instrument at Novogene Co., Ltd 150-bp paired-end reads and 7.5 G raw data per sample.

Sample preparation, and liquid chromatography-mass spectrometry (LC-MS) analysis

Plasma, urine, stool, and adipose tissue samples for the untargeted metabolite profiling were prepared as previously described(4). Plasma, stool, urine, and adipose tissue samples from 15 subjects except one end point sample in plasma (missing), and adipose tissue were analysed using liquid chromatography (reverse-phase, and hydrophilic interaction liquid chromatography), and mass spectrometry with an ultra-high performance liquid chromatography system (Vanquish Flex UHPLC system, Thermo Scientific, Bremen, Germany) coupled to a high-resolution mass spectrometer (Q Exactive Focus, Thermo Scientific, Bremen, Germany). The data was acquired in positive (ESI+), and negative (ESI−) electrospray ionization modes. Data dependent MS2 were acquired for each mode. The technical details of the chromatographic methods, and LC-MS instrument configurations have been described previously(8).

Data matrix generation of metabolomics

Automated peak picking and alignment was done by MS-DIAL (Version 4.90)(9) after the conversion of the raw instrumental data (*.d files) to an ABF format using Reifycs Abf Converter (https://www.reifycs.com/AbfConverter), as previously described(4). A total of 236,666 features were obtained from the peak-picking from the four analytical modes.

Data analysis of metabolomics

Data pre-processing was done separately for each sample matrices and analytical modes using R version 3.6.2. Low-quality features were flagged and discarded from the main results of statistical analyses. Molecular features were only considered high-quality if they met all the following quality metrics: low number of missing values (present in more than 70% of the QC samples, present in at least 50% of samples in at least one study group). Missing values were imputed using simple imputation with value of 0 for all features.

Metabolite identification

For the metabolite identification, only those features with a MS/MS spectrum, average peak area of at least 10,000 per sample type, and raw p-value <0.05 fromthe feature-wise paired t-tests were selected. These chosen signals were annotated using MS-DIAL Version 4.90(9) by

comparing the exact m/z, retention time, and MS/MS fragmentation patterns against our in-house standard library (ca. 1000 metabolites). Further, additional searches in online MS spectral databases were also performed(10–13). Additionally, MS-FINDER Version 3.52(14) was used to characterize the unknowns. Moreover, the vendor software FreeStyle 1.3 was used for the exploration of raw data extracted ion chromatograms (EICs) and MS/MS fragmentation spectra.

Additionally, fragment similarity searches in METLIN database(11) were performed for the top 10 compounds resulting from the analysis (feature-wise) with Maaslin2 version 1.8.0(15) with the formula feature ~ visit with Participant ID as random effect.

Supplementary statistical analysis

Clinical outcomes

Differences in baseline clinical characteristics between the groups were tested using paired T-test if the data were normally distributed. Skewed data were tested by Mann Whitney's U-test. The distribution of the data was assessed by Kolmogorov–Smirnov test as well as by visual inspection of histograms, and Q-Q plots. To test differences between the time points, for continuous outcomes two types of linear mixed models were fit. For the within group comparisons, the data was subset per intervention group and a linear mixed effects model (lmer in R package lme4 version 1.1-34) was fit using the following formula: outcome ~ visit + (1 | ID). For the between group comparisons the entire dataset was used and a linear mixed effects model (lmer in R package lme4 version 1.1-34) was fit using the following formula: outcome ~ visit * intervention + (1 | ID). All outcomes were scaled before fitting the model. The following variables were log transformed before scaling for the linear model analyses: VO_{2max} (L/min), $VO_{2max/}$ (ml/kg/min), weight, BMI, waist circumference, lean mass, fasting glucose, insulin, HbA1c, HOMA-IR, TC, LDL-C, triglycerides, ALT, AST, GGT, liver fat (MRI-PDFF), liver stiffness (MRE), carbohydrates (E-%) and protein (E-%). This selection was based on visual inspection of QQ-plots and Shapiro-Wilk test for normality, to ensure the necessary assumptions were met.

Statistical analysis of tissue RNA expression

The average read count per sample was 49,7 +- 6,6 million. The reads were quality controlled with Trimmomatic version 0.38 with the following parameters: minimum length after trimming = 36, sliding window width = 4, sliding window threshold q score = 15, headcrop = 5. The quality-controlled reads were then pseudo-mapped with Kallisto version 0.45 to *Homo sapiens* transcriptome GRCh38 release 106 with the following parameters: -b 100 –bias. A quality control analysis was performed, where one baseline muscle sample was removed due to close clustering with all adipose tissue samples based on a principal coordinate analysis of the Bray-Curtis distance of all samples (see Supplementary Fig. 3).Only participants with paired samples were kept (liver: 13, muscle: 14, adipose tissue: 15). Differential gene expression was tested per sample type (liver: 29,443 genes, adipose tissue: 32,468 genes, muscle: 29,516 genes) (DESeq2 (16) version 1.34.0, formula: Gene ~ participant ID + visit, with a Wald test and parametric fit). Gene set enrichment was estimated with enrichR (17) version 3.1 with GO Biological Process (2018). Details of deposited data in Supplementary CTAT Table.

Statistical analysis of gut microbiota composition

The average read count per sample was 47,1 +- 6,4 million. Shotgun sequencing reads were quality controlled, and adapters were trimmed with fastp(18) version 0.20.0, on average 46,7 +- 6,5 million reads passed filtering (99%). The trimmed reads were mapped to *Homo sapiens* genome GRCh37 hg19 using Bowtie 2(19) version 2.3.5 (with parameters—very-sensitive, and –dovetail), on average 0.13% +- 0.21% of reads mapped to the human genome. Reads that did not map to the human genome were profiled with mOTUs(20) version 3.0.1. The relative abundance of 1,417 microbial taxa was quantified using mOTUs. In total 244 species present in >75% of participants were tested for differential abundance (ANCOM-BC version 1.4.0 (21), formula: taxon ~ ID + visit, based on the raw count data). Alpha diversity was calculated using the R package vegan version 2.6-4 (function diversity, the parameter index was set to shannon), richness was calculated using the function specnumber, and eveness was calculated as Shannon index / log(richness). Beta diversity was calculated using the vegdist function, with method set to bray or jaccard. Beta diversity was also calculated with the Aitchison distance (chemometrics package version 1.4.2 function clr with a pseudocount of 1 added to the count data, then vegdist with method set to euclidean). Difference in alpha diversity between the visits was tested with a paired Wilcoxon rank sum test (two sided) or Kruskal-Wallis test (two-sided). Difference in beta diversity between the visits was calculated with a permutation multivariate analysis of variance (PERMANOVA), function adonis2 (distance ~ visit, permutations = 9999) with permutation strata by participant ID. Multivariate dispersions of the beta diversity was calculated with the betadisper function with a pairwise permutation test. Enterotypes were calculated based on unfiltered counts at genus level, using Bray-Curtis distance (calculated as explained above) with partitioning around medoids clustering using the clustering R package version 2.1.4 . The number of clusters were determined based on the Calinski-Harabasz index from the fpc R package version 2.2-99, and the most abundant taxa per cluster was used to characterize the clusters.

Microbial pathway analysis

The metagenome reads that passed filtering and did not map to the human genome were processed with HUMANn3 with the search-mode uniref90 to generate microbial functional profiles. The data was normalized to counts per million, and nonstratified data was extracted. The unintegrated, and unmapped pathways were removed, which yielded 414 pathways. The

pathway data was then filtered by first selecting pathways with a median abundance in the top 75%, and then by keeping pathways with a variance in the top 50%. This resulted in 156 pathways that was analyzed with paired Wilcoxon test.

Statistical analysis of metabolites

After the preprocessing, and data clean-up, 11,306 molecular features in plasma samples, 8,393 in adipose tissue samples, 24,171 in urine samples, and 25,166 in stool samples were considered of high quality. The high number of molecular features before data clean-up is due to the high sensitivity of the instrument, collecting several signals from each actual metabolite, but also from the solvent background, and detector noise. For the purpose of metabolite identification, feature-wise paired t-tests were run for all baseline-endpoint pairs in each sample type, and considered significant if the raw p-values were <0.05. Thereafter, all features were filtered to have an average retention time between 1-15 minutes, have MS/MS spectra available, features with zero variance were excluded, and per sample type, features that were present in <50% of any study group were also removed, and for highly correlated pairs of features, only one was kept (using the findCorrelation function from the caret package version 6.0-93, with Spearman R>0.75 as cut-off). The fold change was calculated for each feature to measure the effect size as log2(baseline/end). Only participants with paired samples were kept for analysis (plasma: 14, stool: 15, urine: 15, adipose tissue: 14). In total 1,838 features in plasma, 1,117 features in adipose tissue, 2,158 features in urine, and 2,637 features in stool were analyzed with a linear model (Maaslin2 version 1.8.0(15), formula: feature ~ visit, with Participant ID as random effect).

Multiomics correlation analysis

The baseline, and endpoint omics data (transcriptome, metabolome, metagenome) in the exercise group were compared using Procrustes analysis (in R version 4.1.3 vegan package version 2.6-4). This required paired samples, thus across all datasets 12 samples were used, transcriptomics: 12, and metabolomics: 15. The data was Hellinger transformed (decostand from the vegan package). PCA was performed (rda function from the vegan package), which was rotated, and rescaled using the Procrustes function with the parameter symmetric set to TRUE. Finally, the rotations were tested with a permutational test using the protest function, with 9999 permutations. Additionally, a cross-tissue correlation analysis was performed in Python version 3.7.3 with Conda version 4.7.10. Delta changes (calculated as (End-

Baseline)/(Baseline+End)) of the 5 most FDR p-value significant features from each omic datasets were correlated (Spearman's rho) across datasets. Samples from 8 participants that overlapped across all omics dataset were used. Correlations with rho>0.6 and p-value <0.05 are plotted.

Supplementary figures

Enrollment flow diagram

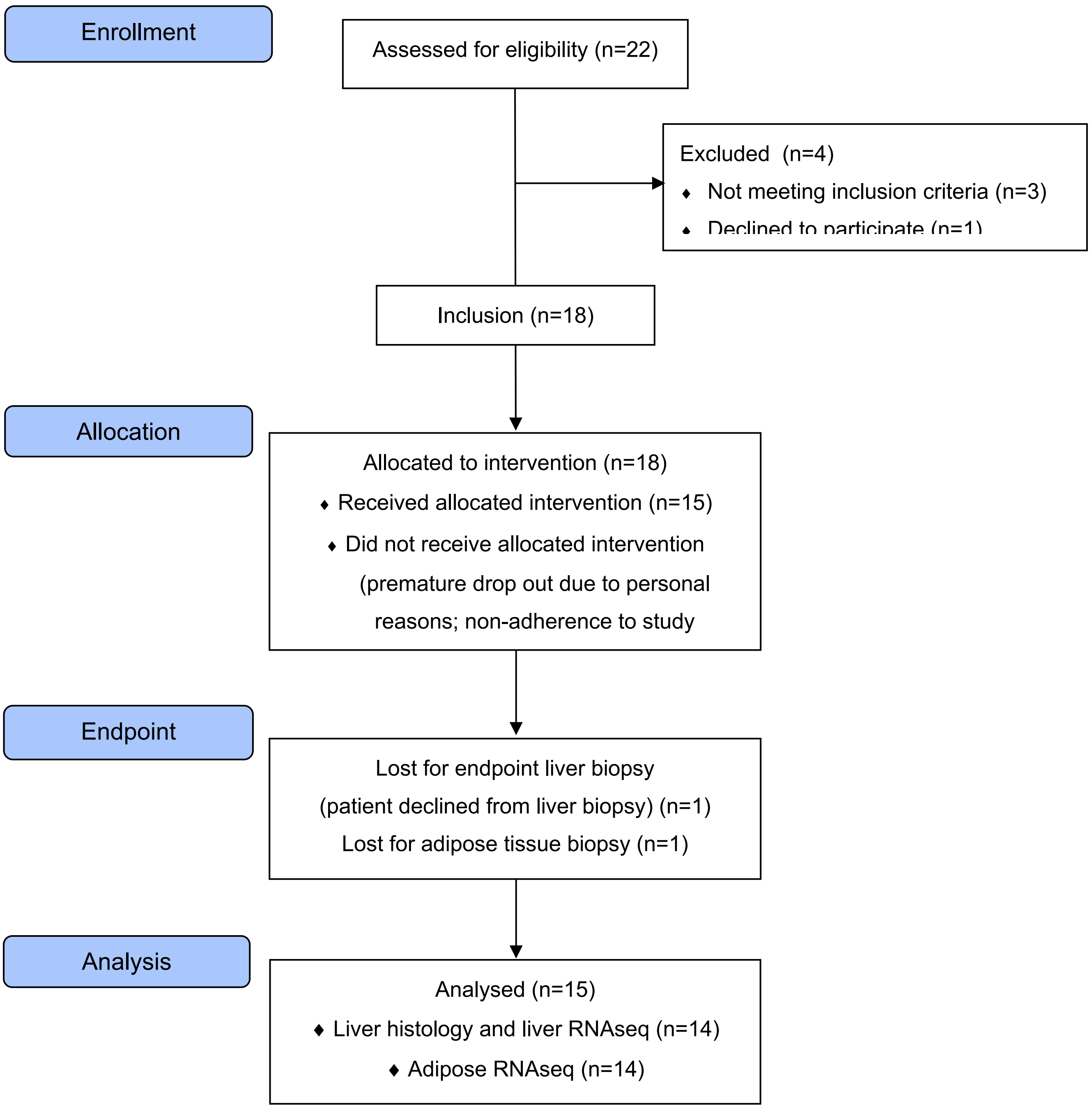

Fig. S1. Enrollment flow diagram adjusted CONSORT 2010 Flow Diagram (22).

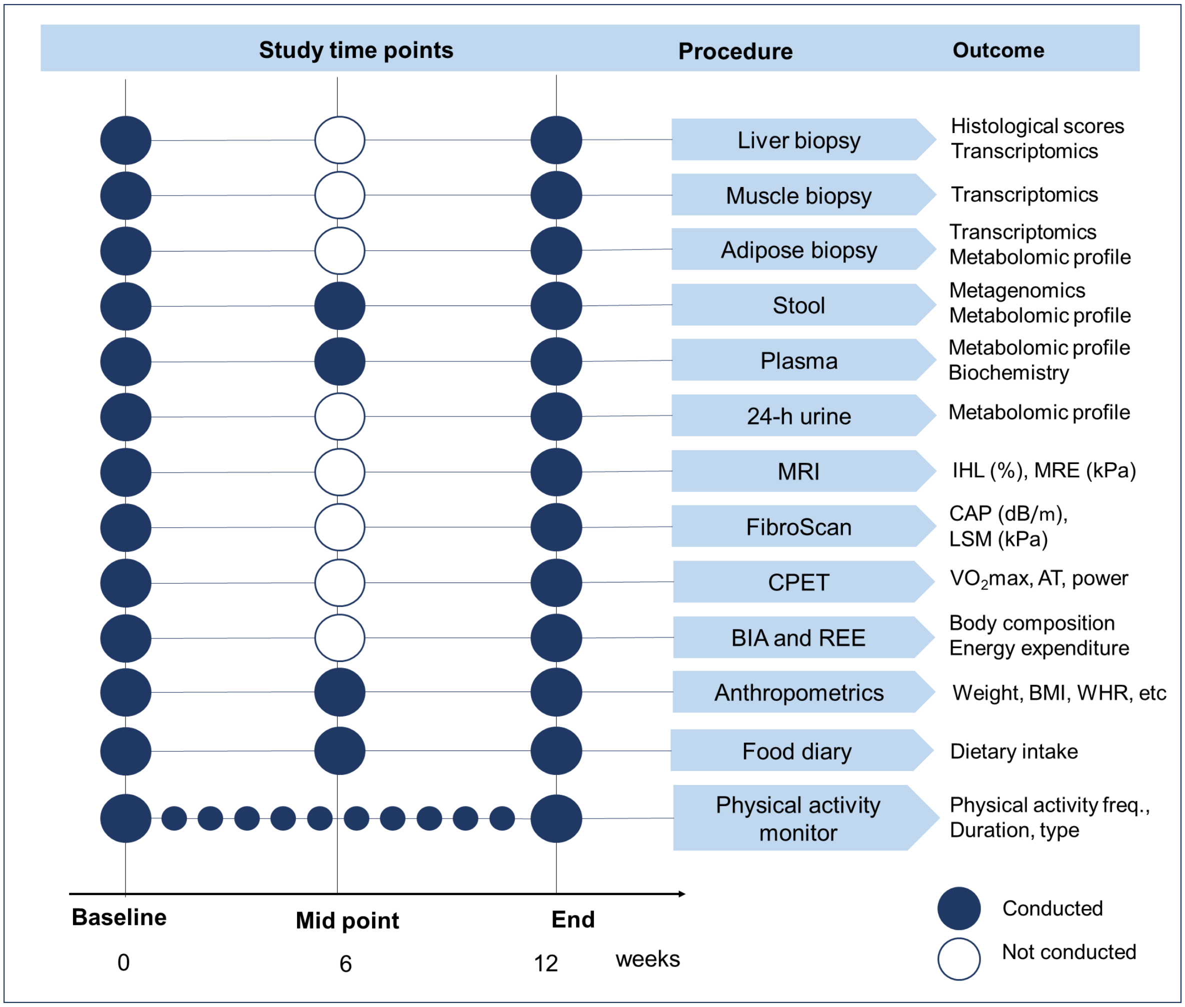

Fig. S2. Study design, procedures and outcomes during the 12-week exercise program. Dark blue circle presents on which time point (baseline, midpoint and endpoint) a procedure was conducted, white circular when a procedure was not conducted. BMI, body mass index; CAP, controlled attenuation parameter; CPET, cardiopulmonary exercise test; dB/m, decibel per meter; LSM, liver stiffness measurement; MRE, magnetic resonance elastrography; MRI, magnetic resonance imaging; kPa, kilopascals; REE, resting energy expenditure; WHR, waist-hip-ratio.

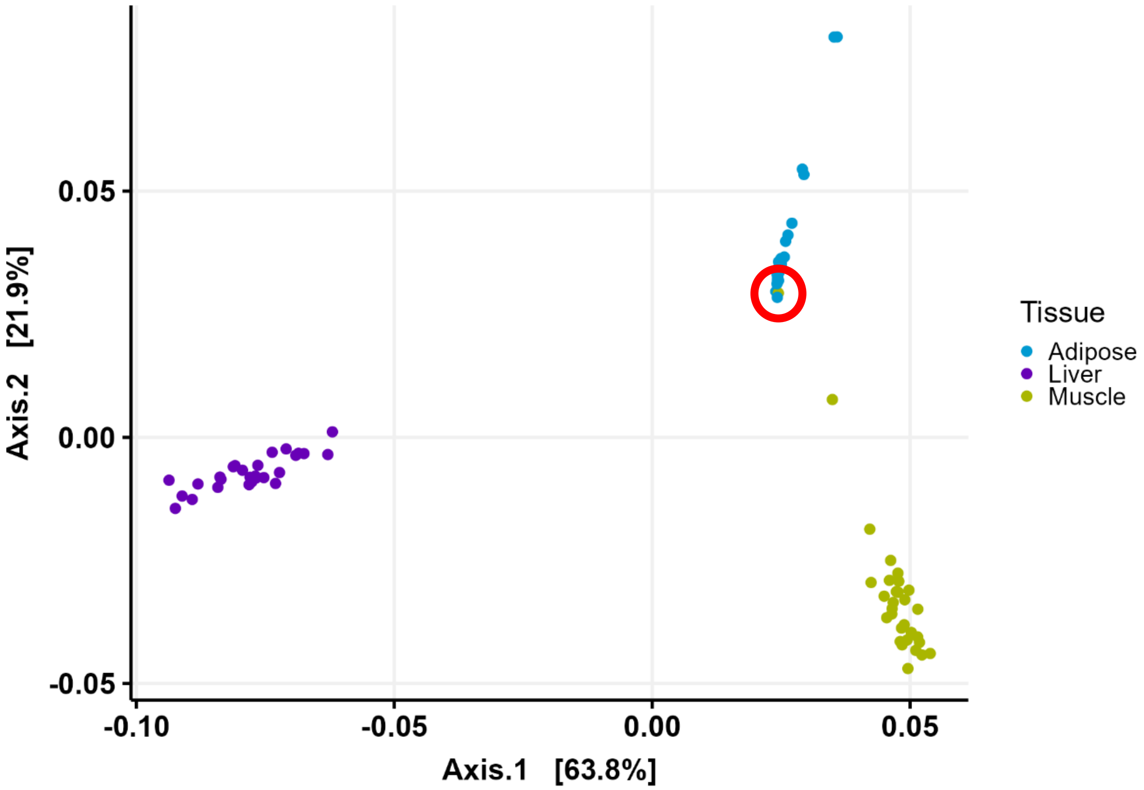

Fig. S3. Quality control of the tissue mRNA sequencing. Liver, adipose and muscle biopsy samples in principal coordinate analysis of the Bray-Curtis distance. Note one muscle (green) sample is clustered within the adipose tissue samples (blue), highlighted in the red circle.

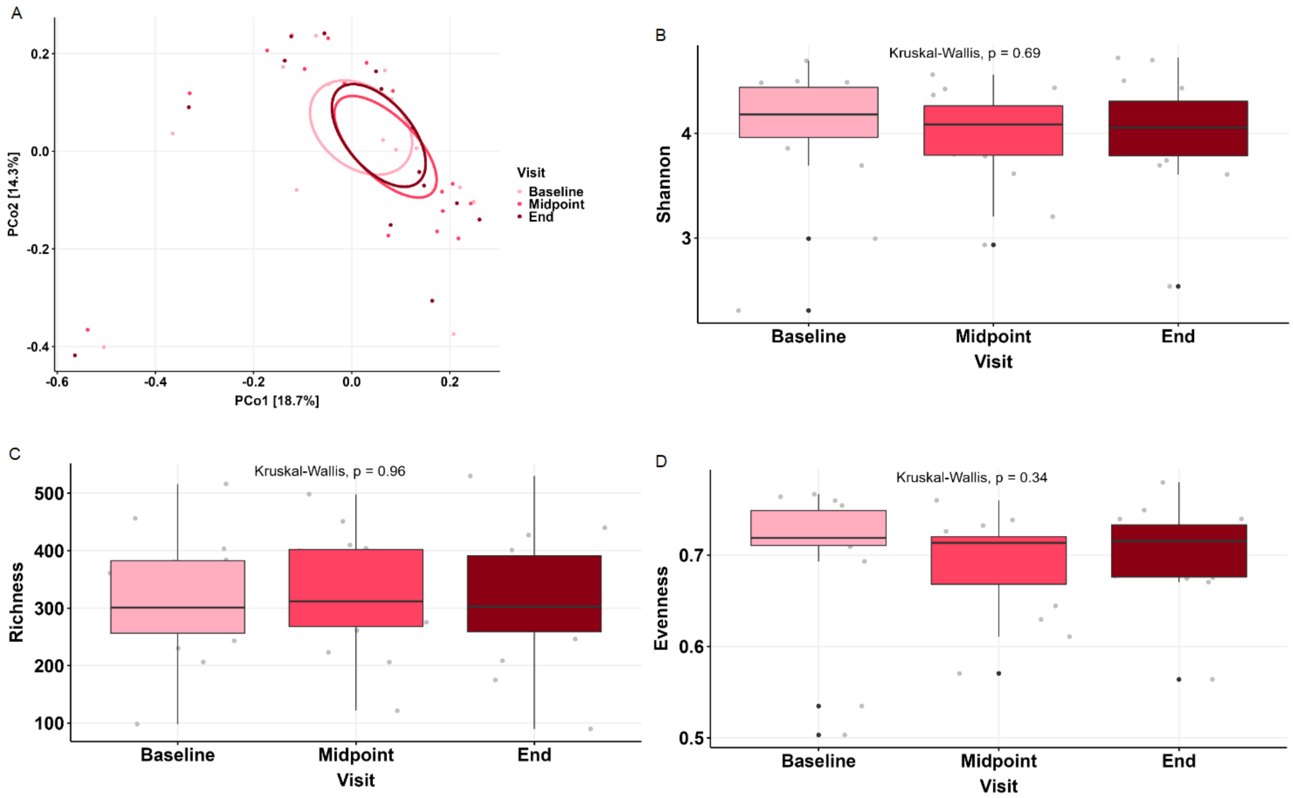

Fig. S4. The composition of the fecal gut microbiota at different time points upon the exercise program. (A) Bray-Curtis β-diversity; (B) Shannon α-diversity; (C) Richness; (D) Evenness of the gut microbiota.

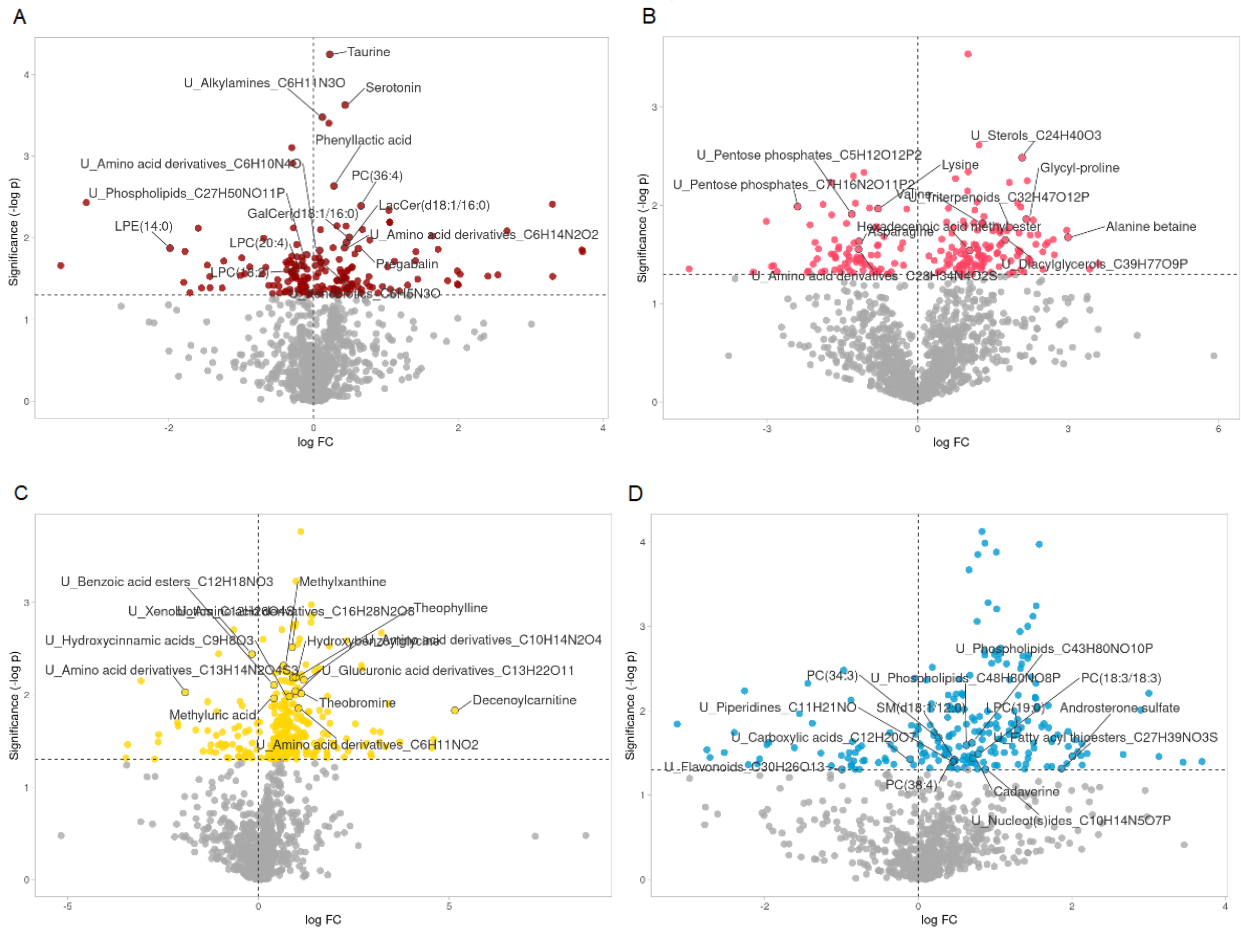

Fig. S5. Results from the paired T-test of metabolomics data per sample type. The x-axis represents the log fold change and the y-axis represents the negative log of the nominal p-value). (A) plasma; (B) stool; (C) urine; (D) adipose tissue.

Supplementary tables

Table S1. See excel file. Results from differential gene expression analysis on muscle, liver, and adipose tissue.

Table S2. See excel file. Spearman correlation results between the relative change of significantly differentially expressed genes (muscle, liver and adipose tissue) with the relative change of VO_2max/kg.

Table S3. See excel file. Spearman correlation results between the relative change significantly differentially expressed liver genes with the relative change of liver fat calculated as MRI-PDFF.

Table S4. See excel file. Spearman correlation results between the relative change significantly differentially expressed adipose tissue genes with the relative change of visceral fat volume.

Table S5. See excel file. Results from the differential abundance analysis of fecal microbial species between baseline and end of the intervention.

Table S6. See excel file. Results from paired Wilcoxon test of fecal microbial pathways.

Table S7. See excel file. Liquid chromatography-mass spectrometry (LC-MS) characteristics and statistical results from the paired t-test and linear mixed model of metabolites from plasma, stool, urine and adipose tissue. For the paired t-test, only features that were nominally significant are included in the table. For the linear mixed model, all features tested are included in the table. The sample type and average abundance based on linear mixed model results.

Table S8. See excel file. Spearman correlation results between the relative change of nominally significant metabolites from any sample type with the relative change of VO_2max/kg.

Table S9A. See excel file. Spearman correlation coefficients between the relative change of multi-omics outcomes.

Table S9B. See excel file. Spearman p-values between the relative change of multi-omics outcomes.

Table S10. See excel file. Procrustes results from baseline and end omics datasets.

Supplementary references

1. Tornvall G. Assessment of Physical Capabilities. Blackwell Scientific Publ. 1963;
2. Taylor HL, Jacobs DR, Schucker B, et al. A questionnaire for the assessment of leisure time physical activities. Journal of Chronic Diseases. 1978 Jan;31(12):741–55.
3. Hakola L, Savonen K, Komulainen P, et al. Moderators of Maintained Increase in Aerobic Exercise Among Aging Men and Women in a 4-Year Randomized Controlled Trial: The DR's EXTRA Study. Journal of physical activity & health. 2015 Nov;12(11):1477–84.
4. Babu AF, Csader S, Männistö V, et al. Effects of exercise on NAFLD using non-targeted metabolomics in adipose tissue, plasma, urine, and stool. Scientific reports. 2022;12(1):6485.
5. Kleiner DE, Brunt EM, Van Natta M, et al. Design and validation of a histological scoring system for nonalcoholic fatty liver disease. Hepatology. 2005 Jun;41(6):1313–21.
6. Bedossa P. Utility and appropriateness of the fatty liver inhibition of progression (FLIP) algorithm and steatosis, activity, and fibrosis (SAF) score in the evaluation of biopsies of nonalcoholic fatty liver disease. Hepatology. 2014 Aug;60(2):565–75.
7. Meijnikman AS, Davids M, Herrema H, et al. Microbiome-derived ethanol in nonalcoholic fatty liver disease. Nature medicine. 2022 Oct;28(10):2100–6.
8. Noerman S, Kokla M, Koistinen VM, et al. Associations of the serum metabolite profile with a healthy Nordic diet and risk of coronary artery disease. Clinical Nutrition. 2021 May;40(5):3250–62.
9. Tsugawa H, Cajka T, Kind T, et al. MS-DIAL: data-independent MS/MS deconvolution for comprehensive metabolome analysis. Nature methods. 2015 Jun;12(6):523–6.
10. Wishart DS, Tzur D, Knox C, et al. HMDB: the Human Metabolome Database. Nucleic acids research. 2007 Jan;35(Database issue):D521-6.
11. Smith CA, O'Maille G, Want EJ, et al. METLIN: a metabolite mass spectral database. Therapeutic drug monitoring. 2005 Dec;27(6):747–51.
12. Kim S, Thiessen PA, Bolton EE, et al. PubChem Substance and Compound databases. Nucleic acids research. 2016 Jan;44(D1):D1202-13.
13. Lipid Maps. Index [Internet]. Available from: https://www.lipidmaps.org/
14. Tsugawa H, Kind T, Nakabayashi R, et al. Hydrogen Rearrangement Rules: Computational MS/MS Fragmentation and Structure Elucidation Using MS-FINDER Software. Analytical chemistry. 2016;88(16):7946–58.
15. Mallick H, Rahnavard A, McIver LJ, et al. Multivariable association discovery in population-scale meta-omics studies. Coelho LP, editor. PLOS Computational Biology. 2021 Nov;17(11):e1009442.

16. Love MI, Huber W, Anders S. Moderated estimation of fold change and dispersion for RNA-seq data with DESeq2. Genome Biology. 2014 Dec;15(12):550.

17. Kuleshov M V., Jones MR, Rouillard AD, et al. Enrichr: a comprehensive gene set enrichment analysis web server 2016 update. Nucleic Acids Research. 2016 Jul;44(W1):W90–7.

18. Chen S, Zhou Y, Chen Y, et al. fastp: an ultra-fast all-in-one FASTQ preprocessor. Bioinformatics. 2018 Sep;34(17):i884–90.

19. Langmead B, Salzberg SL. Fast gapped-read alignment with Bowtie 2. Nature Methods. 2012 Apr;9(4):357–9.

20. Milanese A, Mende DR, Paoli L, et al. Microbial abundance, activity and population genomic profiling with mOTUs2. Nature Communications. 2019 Dec;10(1):1014.

21. Lin H, Peddada S Das. Analysis of compositions of microbiomes with bias correction. Nature Communications. 2020 Dec;11(1):3514.

22. Eldridge SM, Chan CL, Campbell MJ, et al. CONSORT 2010 statement: extension to randomised pilot and feasibility trials. BMJ (Clinical research ed). 2016 Oct;355:i5239.
